# Synthesis, Fungicidal Activity and Plant Protective Properties of 1,2,3-Thiadiazole and Isothiazole-Based *N*-acyl-*N*-arylalaninates

**DOI:** 10.3390/molecules28010419

**Published:** 2023-01-03

**Authors:** Tatiana A. Kalinina, Valeriya I. Balandina, Konstantin L. Obydennov, Pavel A. Slepukhin, Zhijin Fan, Vasiliy A. Bakulev, Tatiana V. Glukhareva

**Affiliations:** 1 Institute of Chemical Engineering, Ural Federal University Named after the First President of Russia B.N. Yeltsin, 19 Mira Street, 620002 Ekaterinburg, Russia; 2 Postovsky Institute of Organic Synthesis, Ural Branch of Russian Academy of Sciences, 22/20 S. Kovalevskaya Street, 620108 Ekaterinburg, Russia; 3 State Key Laboratory of Elemento-Organic Chemistry, College of Chemistry, Nankai University, No. 94, Weijin Road, Tianjin 300071, China

**Keywords:** *N*-acyl-*N*-arylalanines, 1,2,3-thiadiazoles, isothiazoles, antifungal activity, tiadinil, isotianil, plant protection, acylalanine fungicides

## Abstract

The addition of active groups of known fungicides, or systemic acquired resistance inducers, into novel compound molecules to search for potential antifungal compounds is a popular and effective strategy. In this work, a new series of *N*-acyl-*N*-arylalanines was developed and synthesized, in which 1,2,3-thiadiazol-5-ylcarbonyl or 3,4-dichloroisothiazol-5-ylcarbonyl (fragments from synthetic plant resistance activators tiadinil and isotianil, respectively) and a fragment of *N*-arylalanine, the toxophoric group of acylalanine fungicides. Several new synthesized compounds have shown moderate antifungal activity against fungi in vitro, such as *B. cinerea*, *R. solani* and *S. sclerotiorum*. In vivo tests against *A. brassicicola* showed that compound **1d** was 92% effective at a concentration of 200 µg/mL, similar to level of tiadinil, a known inducer of systemic resistance. Thus, **1d** could be considered a new candidate fungicide for further detailed study. The present results will advance research and influence the search for more promising fungicides for disease control in agriculture.

## 1. Introduction

Phytopathogenic fungi are the key pathogens of plant diseases and lead to significant losses in agricultural crops [1,2]. The search for new molecules showing fungicidal activity for the development of plant protection products is a topical area of organic chemistry.

Acylalanine derivatives are a promising class of compounds in the search for new molecules with high fungicidal activity. When such systematic fungicides as furalaxyl, metalaxyl and benalaxyl appeared on the agricultural market in the late 1970s, they showed very good protective properties against oomycetes, especially *Peronosporales*, such as *Phytophthora* spp., *Pseudoperonospora* spp., *Peronospora* spp., *Plasmopara* spp., *Pythium* spp. It was established, on the example of fungi belonging to Peronosporales, that the main mechanism of action of this class of fungicides was specific inhibition of RNA polymerase-1 and blocking of rRNA synthesis [3]. Fungicides of the acylalanine class contain a chiral carbon atom in their alkyl moiety, and the activity of the enantiomers is often different. Accordingly, metalaxyl is a mixture of two enantiomers; however, the fungicidal activity of metalaxyl is almost completely due to its *R*-enantiomer [3]. Many countries use metalaxyl-M instead of racemic metalaxyl to protect agricultural plants, which consists of 97.5% active (*R*)-enantiomer and 2.5% (*S*)-enantiomer as an impurity [4]. The fungicide benalaxyl is a racemic mixture of enantiomers, but both enantiomers are responsible for its fungicidal activity, although *R*-benalaxyl has a higher fungicidal activity and is more toxic to the environment [5].

The uncontrolled use of acylalanine fungicides without alternation with other antifungal agents has caused the spread of resistant strains of phytopathogens [6]. Currently, acylalanine fungicides are used in combination with other pesticides [7]. Of late, this approach in plant protection has seen use everywhere, with the use of combined pesticides becoming increasingly frequent in agriculture.

One modern direction in the protection of plants from microbial infections is the use of compounds capable of inducing the natural defense mechanisms of plants against virulent pathogens [8]. Induced resistance is a transient phenotypic resistance based on the expression of multiple defense genes and is therefore non-specific. It allows plants to be protected from various types of pathogens, including fungi, bacteria and viruses. However, the effectiveness of induced plant resistance may not be the same in relation to different types of pathogenic microorganisms, since some pathogens do not respond to the use of certain resistance inducers. In some cases, increased plant damage was observed. A similar phenomenon was noted, for example, in relation to fungi from the genera *Fusarium* (*F. oxysporum*, *F. solani*), *Rhizoctonia*, *Botritys cinerea* and *Phaeoisariopsis personata* [9,10].

One approaches in the search for promising new molecules for plant protection is the combination, in one molecule, of structural fragments from lead compounds with different biological mechanisms of action [11]. The introduction of such fragments into the structure of the molecule increases the chances of discovering substances with potent biological activity. Additionally, this approach has been implemented to search for compounds with a double mode of action. Thus, combining fragments of antifungal compounds and inducers of plant defense systems in one compound is a potentially valuable strategy for seeking pesticides for complex plant protection.

**Scheme 1:**
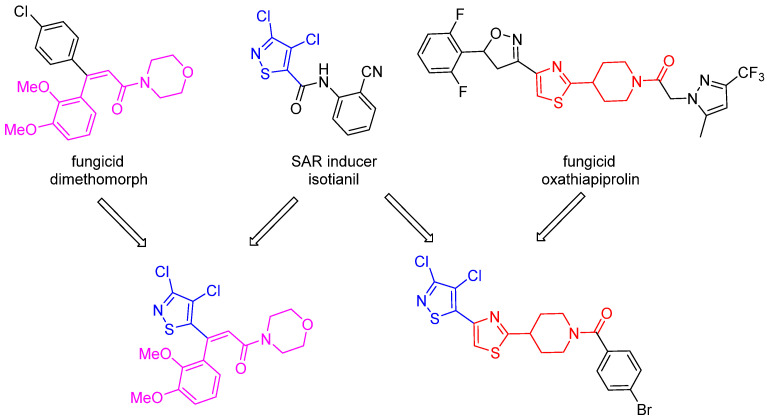
Design strategy of previously synthesized fungicidal compounds.

This strategy was successful when fragments of the known synthetic plant resistance inducer isotianil and fungicides with sinnamic acid derivatives, such as dimethomorph, flumorph and pyrimorph, were combined in new substances [12]. The combinations led to disruption of cell wall biosynthesis and oxathiapiprolin—a piperidinyl thiazole isoxazoline fungicide targeting at oxysterol binding protein [13] (Scheme 1).

**Scheme 2:**
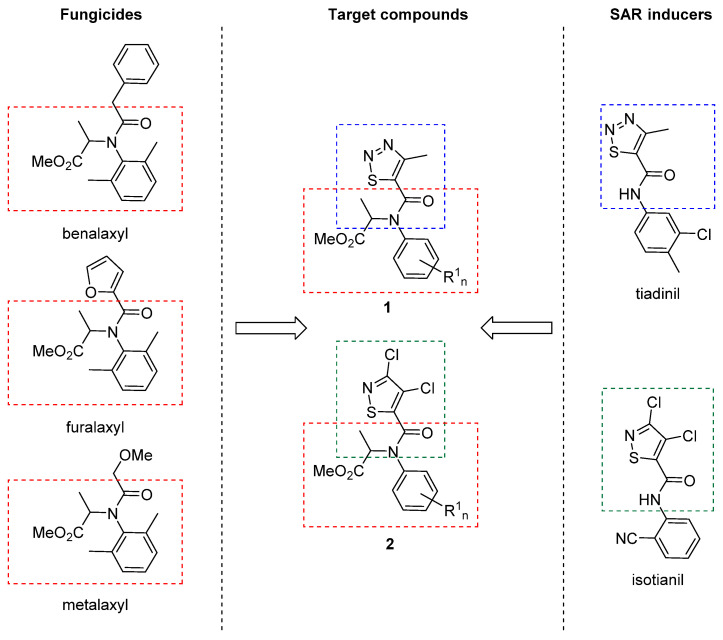
Design strategy of the target compounds.

The aim of this study was the synthesis of combined molecules **1** and **2** (Scheme 2) containing 1,2,3-thiadiazol-5-ylcarbonyl or 3,4-dichloroisothiazol-5-ylcarbonyl fragments, respectively (residues of synthetic plant resistance activators tiadinil and isotianil), as well as a fragment of *N*-arylalanine (the toxophoric group of acylalanine fungicides) (Scheme 2).

The design of the target molecules was realized on the basis of the common acylanilide moiety, which is present both in the acylalanine fungicides and in the SAR inducers tiadinil and isotianil.

## 2. Results and Discussion

### 2.1. Synthesis of Target Compounds

Starting aromatic amines **3a**–**l** were obtained by *N*-alkylation of various anilines **4a**–**l** with methyl-(*RS*)-2-chloropropionate **5** in ethanol in addition of sodium acetate [14,15,16], but the yields were very low. The reaction of aniline with chloropropionate was used as a model reaction to select conditions to increase the reaction yield. The reactions were carried out under previously published conditions for the *N*-alkylation of aromatic amines with alkyl chlorides: in acetonitrile in the presence of potassium carbonate or sodium carbonate [17,18,19,20] and in DMF with potassium carbonate [21,22]. It was found that the highest yield was achieved when the reaction was carried out in DMF in the presence of potassium carbonate. Furthermore, Finkelstein reaction conditions [23] were used, in which the alkylating agent was activated by in situ formation of alkyl iodide when potassium iodide was added to the reaction mixture. The addition of 1.0 equivalents of potassium iodide increased the yield from 50 to 71% and the addition of 2 equivalents of potassium iodide to the reaction mixture increased the reaction yield to 92% (Table 1).

Target compounds **1a**–**l** and **2a**–**l** were synthesized by *N*-acylation of disubstituted amines **3a**–**l** with 4-methyl-1,2,3-thiadiazol-5-ylcarboxylic acid chloride **6a** or 3,4-dichloroisothiazol-5-ylcarboxylic acid chloride **6b**, respectively, in the presence of triethylamine in dry benzene (Table 2). Dry tetrahydrofuran was also used as a solvent, but the reaction yields were much lower.

### 2.2. Structural Analysis

All the corresponding signals of protons and carbons were recorded in the ^1^H and ^13^C NMR spectra of the obtained compounds. However, for compounds **1i** and **2i**, the ^1^H NMR spectra contained a double set of signals for all protons except for the protons of methyl groups in the ortho-positions of the aryl substituent and the methyl group in position 4 of the 1,2,3-thiadiazole ring for compound **1i**. It was observed, from the integrated intensity, that the signals of the protons of the minor isomer of methyl-*N*-acyl-*N*-arylalaninates **1i** and **2i** coincided with the signals of the major isomer. The ratio of isomers, according to ^1^H NMR spectra, was approximately 1:3–1:5. In the ^13^C NMR spectra of compounds **1i** and **2i**, a double set of signals of all carbons as also observed, except for the signal of the carbon of the aryl substituent bound to the nitrogen atom of the amide group at 138.8 ppm for 1,2,3-thiadiazole derivative **1i**. The likelihood was that the formation of diastereoisomer pairs was associated with the formation of the second asymmetric center due to hindered rotation of the ortho-substituted asymmetric aryl fragment of the molecule relative to the C–N σ-bond.

**Figure 1:**
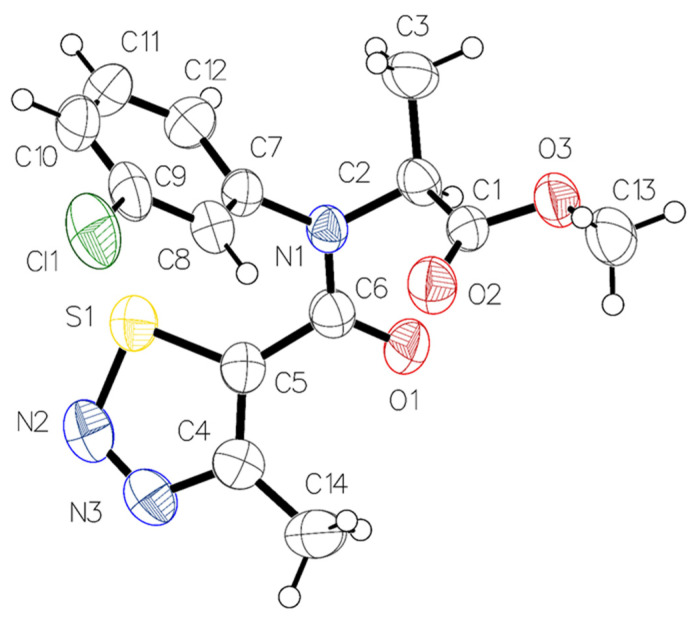
The molecular structure of the compound **1f**, showing the atom numbering scheme for the non-H atoms. Displacement ellipsoids are drawn at the 50% probability level.

The compound **1f** crystallized in the orthorhombic space group *Pbca*. In this case, the N(4) atom had a trigonal pyramidal configuration. The tertiary amine nitrogen atom N(1) had a planar configuration, as the sum of valency angles was 359.4° (Figure 1). The C-N bond lengths of the trisubstituted amine moiety were 1.483(5) Å, 1.434(5) Å, and 1.362(5) Å for C(2)-N(1), C(7)-N(1) and C(6)-N(1), respectively, and corresponded to the lengths of C-Alk, C-Ar and C-N (amide) bonds, respectively, in published crystal structures [24,25,26,27,28,29,30,31,32,33,34,35,36,37]. The N(1), C(2), C(1), and O(2) atoms were in the same plane, with an RMSD of 0.038 Å. The angle between the RMS planes of N(1)C(2)C(1)O(2) and the thiadiazole ring was 51.93°, while the angle between the RMS planes of N(1)C(2)C(1)O(2) and the benzene ring was 122.98°.

**Figure 2:**
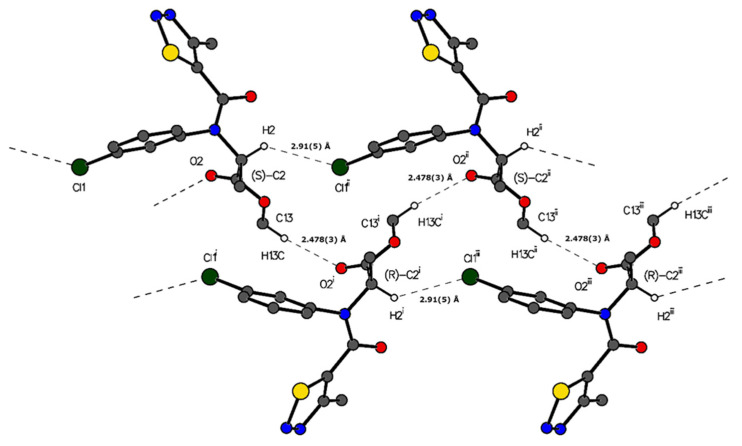
Part of the crystal lattice of compound **1f**, showing the formation of a double chain along the *a*-axis with C(2)H(2)…Cl(1) and C(13)H(13C)…O(2) bonds. For clarity, only those hydrogen atoms which are engaged in the intermolecular interactions are indicated. Symmetry codes: (i) 1/2 + x, +y, 3/2 − z (ii) 1 + x, 2 + y, +z (iii) 3/2 + x, +y, 3/2 − z.

The crystal structure contained molecules of two enantiomers. In enantiomeric pairs, the formation of a C(13)H(13)C…O(2) contacts with the following interaction parameters were observed: *d*(H(13C)…O(2)) = 2.478(3) Å, ∠C(13)H(13C)…O(2) = 157.7(3)° and symmetry operation i) 1/2 + x, +y, 3/2 − z. In the crystal structure between the molecules of the (*S*-) or (*R*-) configuration, there also occurred C(2)H(2)…Cl(1) contacts with the following interaction parameters: *d*(H(2)…Cl(1)) = 2.91(5) Å, ∠C(2)H(2)…Cl(1) = 133(3)° and symmetry operation i) 1 + x, 2 + y, +z. Contacts C(13)H(13C)…O(2) and C(2)H(2)…Cl(1) held enantiomeric pairs together in a double chain in the direction of the *a*-axis (Figure 2).

### 2.3. Fungicidal Activity In Vitro

The fungicidal activity of the target compounds was studied in vitro, according to a previously published method [38], against the oomycete *Phytophthora infestans* (P.i.) and the following phytopathogenic fungi: *Alternaria solani* (A.s.), *Alternaria brassicicola* (A.b.), *Botrytis cinerea* (B.c.), *Colletotrichum coccodes* (C.c.), *Fusarium solani* (F.s.), *Plenodomus lingam* (P.l.), *Rhizoctonia solani* (R.s.) and *Sclerotinia sclerotiorum* (S.s.). The fungicidal properties of *N*-acyl-*N*-arylalaninate derivatives were studied at a concentration of 100 μg/mL. The commercial fungicide carbendazim was used as a positive control. The obtained data of fungicidal activity for the studied compounds are shown in Table 3.

**Figure 3:**
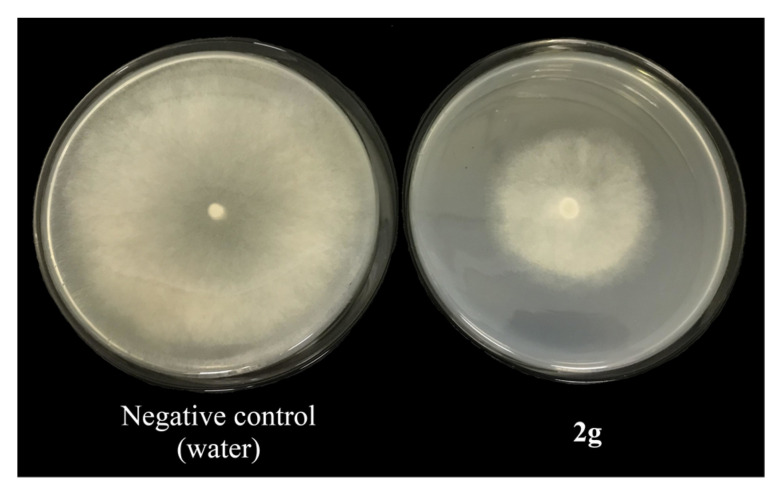
Growth inhibition of *S. sclerotiorum* in addition of compound **2g** to the medium at a concentration of 0.1 mg/mL.

Compounds with moderate fungicidal activity were identified in the series of 3,4-dichloroisothiazole derivatives. Thus, compounds **2a** and **2g** inhibited the radial growth of *B. cinerea*, *R. solani* and *S. sclerotiorum* by more than 60% (Figure 3). Compounds **1h** and **2h**, containing a 3,5-dichloroaryl substituent in their structure, also exhibited moderate fungicidal activity against *S. sclerotiorum*. It should be noted that 3,4-dichloroisothiazole derivatives were more effective compared to 1,2,3-thiadiazole derivatives containing similar aryl substituents.

### 2.4. Fungicidal and Protective Properties In Vivo

**Figure 4:**
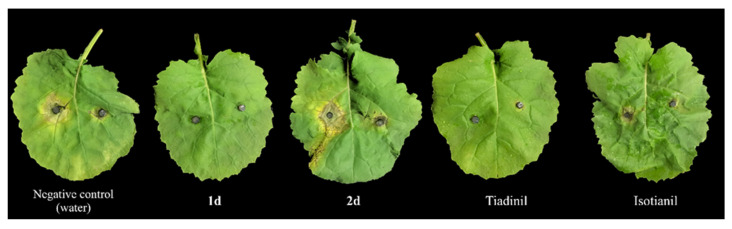
Effects of compound **1d** and **2d** against *A. brassicicola*-infected rape leaves.

The fungicidal and protective properties of two derivatives of *N*-acyl-*N*-arylalaninates, at a concentration of 200 μg/mL, were studied in vivo on rapeseed leaves. Compounds **1d** and **2d** were chosen for the experiment. They contained, in their structure, a 2,6-dimethylphenyl substituent, which is also contained in the structure of fungicides such as metalaxyl and furalaxyl. Two synthetic systemic resistance inducers in plants, tiadinil and isotianil, were used as reference substances. Compound **1d**, containing a 1,2,3-thiadiazole ring in its structure, was shown to completely inhibit leaf damage and prevent the formation of necrotic spots (Table 4, Figure 4). At the same time, the protective properties of compound **1d** turned out to be higher than those of tiadinil. Compound **2d**, at the studied concentration, almost entirely failed to inhibit leaf damage, just like isotianil, another commercial inducer of plant SAR.

Since it was shown in vitro that the 1,2,3-thiadiazole derivative **1d** exhibited very weak fungicidal activity against *A. brassicicola* (I = 12.05 ± 0.08%), it could be assumed that compound **1d** either stimulated the protective properties of rapeseed plants or became more active in vivo.

Thus, we showed the study of the obtained substances **1a**–**l**, **2a**–**l** in vivo, as well as the expansion of their range, to be a promising direction for the search for new substances for plant protection, despite the low fungicidal activity in vitro. The study of the mechanism and spectrum of biological action of compound **1d** would be equally enticing.

## 3. Materials and Methods

### 3.1. Chemical Synthesis

^1^H and ^13^C NMR spectra were recorded with a Bruker Avance II (Karlsruhe, Germany) spectrometer (400 MHz for 1H, 100 MHz for ^13^C). The NMR spectra of all compounds are demonstrated in the Supporting Information (Figures S1–S53). Mass spectra were recorded with a Shimadzu GCMS-QP 2010 “Ultra” (Kyoto, Japan) in electron ionization (EI) mode (electron energy 70 eV). The Fourier transform infrared (FT-IR) spectra were obtained using a Bruker Alpha (ATR, ZnSe) spectrometer (Ettlingen, Germany). Elemental analyses were performed with a Perkin-Elmer 2400Series II CHNS/O analyzer (Shelton, CT USA). Melting points were determined using a Stuart SMP 3 apparatus (Staffordshire, ST15 OSA, UK). The progress of the reactions and the purity of the compounds were monitored by TLC on TLC Silica gel 60 F245 aluminum sheets (Merck KGaA) in an EtOAc-hexane system (1:9 or 1:5).

#### 3.1.1. General Procedure for Preparation of Methyl *N*-aryl-(*RS*)-alaninates (**3a**–**l**)

Aniline derivatives (10.0 mmol) were dissolved in 15 mL of dry DMF. Then, 1.0 eq. of potassium carbonate and 2.0 eq. of potassium iodide were added and 12.0 mmol of methyl-(*RS*)-2-chloropropionate was added dropwise. The reaction mixture was heated with stirring to 80 °C for 16–20 h. The precipitate was filtered off and washed with DMF. The filtrate was then poured into 100 mL of water and extracted with ethyl acetate (3 × 100 mL). The combined organic fractions were washed with water (3 × 100 mL) and 1N HCl (2 × 100 mL), and dried over anhydrous Na_2_SO_4_. Thereafter, the solvent was distilled off. Purification was carried out using column chromatography; eluent was ethyl acetate/hexane 1:5 or 1:9. The solvent was distilled off from the combined fractions after elution without pre-drying.

Methyl *N*-phenyl-(*RS*)-alaninate (**3a**) [39]. Yellow oil, yield 1.649 g (92%). ^1^H NMR (400 MHz, DMSO-d_6_) *δ* 7.07 (dd, *J* = 7.6, 8.1 Hz, 2H, CH Ar), 6.58–6.52 (m, 3H, CH Ar), 5.95 (d, *J* = 8.2 Hz, 1H, NH), 4.10–4.02 (m, 1H, CH), 3.62 (s, 3H, OCH_3_), 1.36 (d, *J* = 7.0 Hz, 3H, CH_3_); EI-MS *m*/*z* (%): 179 [M]^+^ (11), 120 (100), 104 (5), 91 (4), 77 (15).

Methyl *N*-(*m*-tolyl)-(*RS*)-alaninate (**3b**). Yellow oil, yield 1.469 g (76%). ^1^H NMR (400 MHz, DMSO-d_6_) *δ* 6.94 (dd, *J* = 7.7, 7.7 Hz, 1H, CH Ar), 6.39–6.31 (m, 3H, CH Ar), 5.85 (d, *J* = 7.7 Hz, 1H, NH), 4.08–4.01 (m, 1H, CH), 3.62 (s, 3H, OCH_3_), 2.17 (s, 3H, CH_3_), 1.36 (d, *J* = 7.0 Hz, 3H, CH_3_); EI-MS *m*/*z* (%): 194 [M+1]^+^ (2), 193 [M]^+^ (14), 134 (100), 132 (5), 119 (12), 118 (11), 91 (18), 77 (5).

Methyl *N*-(*p*-tolyl)-(*RS*)-alaninate (**3c**). Yellow oil, yield 1.527 g (79%). ^1^H NMR (400 MHz, DMSO-d_6_) *δ* 6.88 (d, *J* = 8.2 Hz, 2H, CH Ar), 6.45 (d, *J* = 8.2 Hz, 2H, CH Ar), 5.73 (d, *J* = 8.5 Hz, 1H, NH), 4.06–3.98 (m, 1H, CH), 3.61 (s, 3H, OCH_3_), 2.14 (s, 3H, CH_3_), 1.36 (d, *J* = 7.0 Hz, 3H, CH_3_); EI-MS *m*/*z* (%): 193 [M]^+^ (13), 134 (100), 118 (11), 91 (15), 77 (5).

Methyl *N*-(2,6-dimethylphenyl)-(*RS*)-alaninate (**3d**) [39]. Yellow oil, yield 1.824 g (88%). ^1^H NMR (400 MHz, DMSO-d_6_) *δ* 6.92 (d, *J* = 7.4 Hz, 2H, CH Ar), 6.73 (t, *J* = 7.4 Hz, 1H, CH Ar), 4.11 (d, *J* = 11 Hz, 1H, NH), 3.91–3.83 (m, 1H, CH), 3.57 (s, 3H, OCH_3_), 2.22 (s, 6H, 2CH_3_), 1.34 (d, *J* = 6.9 Hz, 3H, CH_3_); EI-MS *m*/*z* (%): 207 [M]^+^ (16), 148 (100), 132 (10), 105 (10), 77 (13).

Methyl *N*-(2,4,6-trimethylphenyl)-(*RS*)-alaninate (**3e**) [40]. Orange oil, yield 1.284 g (58%). ^1^H NMR (400 MHz, DMSO-d_6_) *δ* 6.72 (s, 2H, CH Ar), 3.97 (d, *J* = 6.9 Hz, 1H, NH), 3.83–3.74 (m, 1H, CH), 3.58 (s, 3H, OCH_3_), 2.18 (s, 6H, 2CH_3_), 2.13 (s, 3H, CH_3_), 1.31 (d, *J* = 6.9 Hz, 3H, CH_3_); EI-MS *m*/*z* (%): 221 [M]^+^ (3), 176 (2), 162 (100), 146 (7), 132 (8), 119 (7), 103 (3), 91 (13), 77 (7).

Methyl *N*-(3-chlorolphenyl)-(*RS*)-alaninate (**3f**) [41]. Dark yellow oil, yield 1.624 g (76%). ^1^H NMR (400 MHz, DMSO-d_6_) *δ* 7.07 (dd, *J* = 8.2, 8.3 Hz, 1H, CH Ar), 6.57–6.55 (m, 2H, CH Ar), 6.49 (d, *J* = 7.7 Hz, 1H, CH Ar), 6.32 (d, *J* = 8.1 Hz, 1H, NH), 4.14–4.07 (m, 1H, CH), 3.63 (s, 3H, OCH_3_), 1.37 (d, *J* = 6.9 Hz, 3H, CH_3_); EI-MS *m*/*z* (%): 215 [M+2]^+^ (5), 213 [M]^+^ (15), 156 (32), 154 (100), 153 (5), 138 (5), 119 (13), 118 (17), 111 (8), 99 (2), 91 (6), 77 (5), 75 (10).

Methyl *N*-(4-chlorolphenyl)-(*RS*)-alaninate (**3g**) [39]. Light yellow oil, yield 1.667 g (78%). ^1^H NMR (400 MHz, DMSO-d_6_) *δ* 7.09 (d, *J* = 8.8 Hz, 2H, CH Ar), 6.55 (d, *J* = 8.8 Hz, 2H, CH Ar), 6.17 (d, *J* = 8.2 Hz, 1H, NH), 4.10–4.03 (m, 1H, CH), 3.62 (s, 3H, OCH_3_), 1.37 (d, *J* = 7.0 Hz, 3H, CH_3_). EI-MS *m*/*z* (%): 215 [M+2]^+^ (4), 213 [M]^+^ (13), 156 (32), 154 (100), 138 (6), 119 (29), 118 (22), 111 (9), 99 (4), 91 (7).

Methyl *N*-(3,5-dichlorolphenyl)-(*RS*)-alaninate (**3h**). Light brown oil, yield 1.319 g (53%). ^1^H NMR (400 MHz, DMSO-d_6_) *δ* 6.65 (d, *J* = 1.7 Hz, 1H, CH Ar), 6.62 (d, *J* = 8.2 Hz, 1H, NH), 6.56 (d, *J* = 1.7 Hz, 2H, CH Ar), 4.18 (p, *J* = 7.0 Hz, 1H, CH), 3.65 (s, 3H, OCH_3_), 1.36 (d, *J* = 7.0 Hz, 3H, CH_3_); EI-MS *m*/*z* (%): 251 [M+4]^+^ (2), 249 [M+2]^+^ (10), 247 [M]^+^ (17), 192 (10), 190 (64), 188 (100), 155 (6), 153 (19), 152 (11), 138 (3), 117 (12), 91 (4).

Methyl *N*-(2-methyl-3-chlorolphenyl)-(*RS*)-alaninate (**3i**). Yellow oil, yield 1.662 g (73%). ^1^H NMR (400 MHz, DMSO-d_6_) *δ* 6.98 (dd, *J* = 7.9, 8.2 Hz, 1H, CH Ar), 6.70 (d, *J* = 7.9 Hz, 1H, CH Ar), 6.37 (d, *J* = 8.2 Hz, 1H, CH Ar), 5.27 (d, *J* = 8.1 Hz, 1H, NH), 4.20–4.10 (m, 1H, CH), 3.64 (s, 3H, OCH_3_), 2.21 (s, 3H, CH_3_), 1.46 (d, *J* = 7.0 Hz, 3H, CH_3_); EI-MS *m*/*z* (%): 229 [M+2]^+^ (6), 227 [M]^+^ (18), 170 (33), 168 (100), 152 (6), 132 (8), 118 (16), 117 (15), 104 (3), 89 (14), 77 (8).

Methyl *N*-(3-methoxyphenyl)-(*RS*)-alaninate (**3j**) [42]. Orange oil, yield 1.548 g (74%). ^1^H NMR (400 MHz, DMSO-d_6_) *δ* 6.97 (dd, *J* = 8.1, 8.1 Hz, 1H, CH Ar), 6.17–6.10 (m, 3H, CH Ar), 5.99 (d, *J* = 8.2 Hz, 1H, NH), 4.08–4.01 (m, 1H, CH), 3.66 (s, 3H, OCH_3_), 3.63 (s, 3H, OCH_3_), 1.36 (d, *J* = 7.0 Hz, 3H, CH_3_); EI-MS *m*/*z* (%): 209 [M]^+^ (15), 150 (100), 135 (12), 134 (6), 118 (4), 107 (11), 92 (9), 77 (11).

Methyl *N*-(4-methoxyphenyl)-(*RS*)-alaninate (**3k**) [43]. Yellow oil, yield 1.276 g (61%). ^1^H NMR (400 MHz, DMSO-d_6_) *δ* 6.70 (d, *J* = 8.8 Hz, 2H, CH Ar), 6.50 (d, *J* = 8.8 Hz, 2H, CH Ar), 5.54 (d, *J* = 8.8. Hz, 1H, NH), 4.03–3.95 (m, 1H, CH), 3.63 (s, 3H, OCH_3_), 3.60 (s, 3H, OCH_3_), 1.34 (d, *J* = 7.0 Hz, 3H, CH_3_); EI-MS *m*/*z* (%): 209 [M]^+^ (18), 150 (100), 135 (12), 134 (12), 122 (4), 119 (5), 118 (4), 108 (5), 107 (7), 106 (6), 92 (5), 80 (5), 77 (7).

Methyl 3-trifluoromethylphenyl-(*RS*)-alaninate (**3l**) [39]. Light brown oil, yield 2.200 g (89%). ^1^H NMR (400 MHz, DMSO-d_6_) *δ* 7.28 (dd, *J* = 7.7, 7.8 Hz, 1H, CH Ar), 6.86–6.77 (m, 3H, CH Ar), 6.50 (d, *J* = 8.1 Hz, 1H, NH), 4.21–4.14 (m, 1H, CH), 3.63 (s, 3H, CH_3_), 1.39 (d, *J* = 7.0 Hz, 3H, CH_3_); EI-MS *m*/*z* (%): 248 [M+1]^+^ (2), 247 [M]^+^ (14), 228 (4), 189 (11), 188 (100), 172 (4), 148 (3), 145 (11), 119 (8), 118 (6), 95 (4).

#### 3.1.2. General Procedure for Preparation Methyl *N*-(Heteryl-5-carbonyl)-*N*-aryl-(*RS*)-alaninates (**1a**–**l** and **2a**–**l**)

Briefly, 4-Methyl-1,2,3-thiadiazol-5-ylcarboxylic acid chloride, or 3,4-dichloroisothiazolylcarboxylic acid chloride (1.5 mmol), was added dropwise to methyl aryl-(*RS*)-alaninate (1.0 mmol) in 10 mL of dry tetrahydrofuran. While cooling, triethylamine 1.5 mmol was added. The reaction mixture was stirred at 50 °C for 4 h. Water was added and the resultant extracted with ethyl acetate (3 × 25 mL). The combined organic layers were washed with brine, 1N hydrochloric acid, saturated soda and water, then dried under anhydrous sodium sulfate. Ethyl acetate was distilled off under reduced pressure. Purification of the resulting product was carried out using column chromatography with ethyl acetate–hexane 1:9 or 1:5 as eluents. The solvent was distilled off from the combined fractions after elution without pre-drying.

Methyl-*N*-(4-methyl-1,2,3-thiadiazole-5-carbonyl)-*N*-phenyl-(*RS*)-alaninate (**1a**): yellow oil, yield 0.223 g (73%); IR, ν, cm^−1^: 2998, 2951, 1745 (C=O), 1635 (C=O), 1486, 1455, 1455, 1382, 1347, 1260, 1200, 1112, 1049,1021, 981; ^1^H NMR (400 MHz, DMSO-d_6_) *δ* 7.38 (s, 5H, H Ar), 5.03 (q, *J* = 7.2 Hz, 1H, CH), 3.74 (s, 3H, OCH_3_), 2.67 (s, 3H, HetCH_3_), 1.33 (d, *J* = 7.2 Hz, 3H, CH_3_); ^13^C NMR (100 MHz, DMSO-d_6_) *δ* 171.2 (C=O), 160.4 (C=O), 159.3 (C Het), 142.3 (C Het), 138.8 (C Ar), 129.6 (CH Ar), 129.5 (CH Ar), 129.4 (CH Ar), 56.4 (CH), 52.3 (OCH_3_), 14.8 (CH_3_), 13.1 (CH_3_ Het); EI-MS *m*/*z* (%): 305 [M]^+^ (1), 274 [M–CH_3_O]^+^ (2), 245 [M–HCO_2_CH_3_]^+^ (12), 218 [M–CH(CH_3_)CO_2_CH_3_]^+^ (39), 178 [M–HetCO]^+^ (42), 99 [HetCO–N_2_]^+^ (100). Analysis calculated for C_14_H_15_N_3_O_3_S (Mr = 305.35): C 55.07, H 4.95, N 13.76, S 10.50; found: C 54.90, H 5.06, N 13.48, S 10.25%.

Methyl-*N*-(4-methyl-1,2,3-thiadiazole-5-carbonyl)-*N*-(*m*-tolyl)-(*RS*)-alaninate (**1b**): beige powder, yield 0.236 g (74%); m.p. 82–83 °C; IR, ν, cm^−1^: 2994, 2954, 1744 (C=O), 1636 (C=O), 1601, 1485, 1455, 1381, 1367, 1348, 1272, 1244, 1208, 1193, 1179, 1110, 1051, 1016, 977; ^1^H NMR (400 MHz, DMSO-d_6_) *δ* 7.29–7.13 (m, 4H, H Ar), 4.97 (q, *J* = 7.1 Hz, 1H, CH), 3.74 (s, 3H, OCH_3_), 2.67 (s, 3H, CH_3_), 2.27 (s, 3H, CH_3_), 1.33 (d, *J* = 7.1 Hz, 3H, CH_3_); ^13^C NMR (100 MHz, DMSO-d_6_) *δ* 171.2 (C=O), 160.3 (C=O), 159.6 (C Het), 142.2 (C Het), 139.5 (CH Ar), 138.8, 130.1, 129.8, 129.5 (CH Ar), 126.5 (C Ar), 56.6 (CH), 52.3 (OCH_3_), 20.7 (CH_3_), 14.8 (CH_3_), 13.2 (CH_3_); EI-MS *m*/*z* (%): 319 [M]^+^ (2), 290 (3), 288 [M–OCH_3_]^+^ (2), 259 [M–HCO_2_CH_3_]^+^ (10), 232 [M–CH(CH_3_)CO_2_CH_3_]^+^ (26), 192 [M–HetCO]^+^ (40), 148 (12), 132 (19), 118 (36), 99 [HetCO–N_2_]^+·^ (100). Analysis calculated for C_15_H_17_N_3_O_3_S (Mr = 319.38): C 56.41, H 5.37, N 13.16, S 10.04; found: C 56.26, H 5.25, N 12.87, S 10.15%.

Methyl-*N*-(4-methyl-1,2,3-thiadiazole-5-carbonyl)-*N*-(*p*-tolyl)-(*RS*)-alaninate (**1c**): light yellow oil, yield 0.217 g (68%); IR, ν, cm^−1^: 2992, 2951, 1743 (C=O), 1642 (C=O), 1605, 1578, 1510, 1452, 1382, 1331, 1275, 1207, 1151, 1110, 1088, 1046, 1017, 978; ^1^H NMR (400 MHz, DMSO-d_6_) *δ* 7.24 (d, 2H, *J* = 8.2 Hz, H Ar), 7.20 (d, 2H, *J* = 8.2 Hz, H Ar), 5.01 (q, 1H, *J* = 7.3 Hz, CH), 3.73 (s, 3H, OCH_3_), 2.67 (s, 3H, HetCH_3_), 2.28 (s, 3H, CH_3_), 1.30 (d, 3H, *J* = 7.3 Hz, CH_3_); ^13^C NMR (100 MHz, DMSO-d_6_) *δ* 171.3 (C=O), 160.5 (C=O), 159.5 (C Het), 142.3 (C Het), 139.2 (C Ar), 136.1 (C Ar), 130.1 (C Ar), 129.3 (C Ar), 56.2 (CH), 52.3 (OCH_3_), 20.6 (CH_3_), 14.8 (CH_3_), 13.1 (CH_3_); EI-MS *m*/*z* (%): 319 [M]^+^ (5), 288 [M–OCH_3_]^+^ (2), 259 [M–HCO_2_CH_3_]^+^ (9), 232 [M–CH(CH_3_)CO_2_CH_3_]^+^ (36), 192 [M–HetCO]^+^ (42), 99 [HetCO–N_2_]^+^ (100). Analysis calculated for C_15_H_17_N_3_O_3_S (Mr = 319.38): C 56.41, H 5.37, N 13.16, S 10.04; found: C 56.33, H 5.13, N 13.21, S 9.87%.

Methyl-*N*-(4-methyl-1,2,3-thiadiazole-5-carbonyl)-*N*-(2,6-dimethylphenyl)-(*RS*)-alaninate (**1d**): white powder, yield 0.230 g (69%); m.p. 127–128 °C; IR, ν, cm^−1^: 2996, 2948, 1743 (C=O), 1636 (C=O), 1462, 1394, 1355, 1322, 1269, 1243, 1201, 1151, 1121, 1099, 1012, 974; ^1^H NMR (400 MHz, DMSO-d_6_) *δ* 7.37 (t, *J* = 7.5 Hz, 1H, H Ar), 7.26–7.22 (m, 2H, H Ar), 4.66 (q, *J* = 7.3 Hz, 1H, CH), 3.73 (s, 3H, OCH_3_), 2.82 (s, 3H, CH_3_), 2.24 (s, 3H, CH_3_), 2.15 (s, 3H, CH_3_), 1.09 (d, *J* = 7.3 Hz, 3H, CH_3_); ^13^C NMR (100 MHz, DMSO-d_6_) *δ* 171.4 (C=O ester), 163.0 (C=O), 160.4 (C Het), 139.1 (C Het), 139.0 (C Ar), 138.6 (C Ar), 136.0 (C Ar), 130.4 (CH Ar), 129.7 (CH Ar), 129.5 (CH Ar), 56.0 (CH), 52.1 (OCH_3_), 18.0 (CH_3_), 17.8 (CH_3_), 14.7 (CH_3_), 14.1 (CH_3_); EI-MS *m*/*z* (%): 333 [M]^+^ (1), 302 [M–OCH_3_]^+^ (5), 290 (9), 272 [M–CO_2_CH_3_]^+^ (10), 246 [M–CH(CH_3_)CO_2_CH_3_]^+^ (28), 206 [M–HetCO]^+^ (92), 99 [HetCO–N_2_]^+·^ (100). Analysis calculated for C_16_H_19_N_3_O_3_S (Mr = 333.41): C 57.64, H 5.74, N 12.60, S 9.62; found C 57.39, H 5.54, N 12.39, S 9.48%.

Methyl-*N*-(4-methyl-1,2,3-thiadiazole-5-carbonyl)-*N*-(2,4,6-trimethylphenyl)-(*RS*)-alaninate (**1e**): light yellow powder, yield 0.285 g (82%); m.p. 120–121 °C; IR, ν, cm^−1^: 2989, 2918, 2849, 1753 (C=O), 1634 (C=O), 1474, 1455, 1375, 1350, 1306, 1263, 1209, 1166, 1141, 1112, 1084, 1033, 1010, 981; ^1^H NMR (400 MHz, DMSO-d_6_) *δ* 7.07 (s, 1H, H Ar), 7.05 (s, 1H, H Ar), 4.63 (q, *J* = 7.2, 1H, CH), 3.72 (s, 3H, OCH_3_), 2.82 (s, 3H, CH_3_), 2.31 (s, 3H, CH_3_), 2.19 (s, 3H, CH_3_), 2.09 (s, 3H, CH_3_), 1.08 (d, *J* = 7.3, 3H, CH_3_); ^13^C NMR (100 MHz, DMSO-d_6_) *δ* 171.5 (C=O), 163.0 (C=O), 160.5 (C Het), 140.1 (C Het), 139.0 (C Ar), 138.6 (C Ar), 138.2 (C Ar), 133.4 (C Ar), 130.3 (CH Ar), 130.1 (CH Ar), 56.1 (CH), 52.0 (OCH_3_), 20.6 (CH_3_ Ar), 17.9 (CH_3_ Ar), 17.7 (CH_3_ Ar), 14.7 (CH_3_), 14.2 (CH_3_); EI-MS *m*/*z* (%): 347 [M]^+^ (2), 319 [M–N_2_]^+^ (8), 304 (11), 286 [M–CO_2_CH_3_]^+^ (14), 272 (7), 260 [M–CH(CH_3_)CO_2_CH_3_]^+^ (32), 244 (23), 232 (21), 220 [M–HetCO]^+·^ (99), 146, 119, 99 [HetCO–N_2_]^+^ (100). Analysis calculated for C_17_H_21_N_3_O_3_S (Mr = 347.43): C 58.77, H 6.09, N 12.09, S 9.23; found C 58.65, H 6.12, N 11.83, S 9.01%.

Methyl-*N*-(4-methyl-1,2,3-thiadiazole-5-carbonyl)-*N*-(3-chlorophenyl)-(*RS*)-alaninate (**1f**): white powder, yield 0.330 g (97%); m.p. 107–109 °C; IR, ν, cm^−1^: 2957, 1740 (C=O), 1636 (C=O), 1584, 1472, 1418, 1384, 1347, 1261, 1203, 1121, 1017, 976; ^1^H NMR (400 MHz, DMSO-d_6_) *δ* 7.54 (br. s, 1H, CH Ar), 7.47 (d, *J* = 7.8 Hz, 1H, CH Ar), 7.41 (dd, *J* = 7.8, 7.6 Hz, 1H, CH Ar), 7.36 (d, *J* = 7.6 Hz, 1H, CH Ar), 5.02 (q, *J* = 7.1 Hz, 1H, CH), 3.75 (s, 3H, OCH_3_), 2.65 (s, 3H, CH_3_), 1.35 (d, *J* = 7.1 Hz, 3H, CH_3_); ^13^C NMR (100 MHz, DMSO-d_6_) *δ* 171.1 (C=O ester), 160.4 (C=O), 159.0 (C Het), 142.2 (C Het), 140.2 (C Ar), 133.5 (C Ar), 131.1 (CH Ar), 129.4 (CH Ar), 129.3 (CH Ar), 128.2 (CH Ar), 56.5 (CH), 52.4 (OCH_3_), 14.7 (CH_3_), 12.9 (CH_3_); EI-MS *m*/*z* (%): 341 [M+2]^+^ (1), 339 [M]^+^ (1), 308 [M–OCH_3_]^+^ (2), 279 [M–HCO_2_CH_3_]^+^ (8), 254 (8), 252 (21), 214 [M+2–HetCO]^+^ (9), 212 [M–HetCO]^+^ (27), 192 (2), 168 (7), 152 (5), 138 (14), 113 (8), 111 (24), 99 [HetCO–N_2_]^+^ (100). Analysis calculated for C_14_H_14_ClN_3_O_3_S (Mr = 339.79): C 49.49, H 4.15, N 12.37, S 9.44; found C 49.58, H 4.28, N 12.09, S 9.12%.

Methyl-*N*-(4-methyl-1,2,3-thiadiazole-5-carbonyl)-*N*-(4-chlorophenyl)-(*RS*)-alaninate (**1g**): light yellow oil, yield 0.296 g (87%); IR, ν, cm^−1^: 2994, 2951, 1742 (C=O), 1644 (C=O), 1483, 1454, 1383, 1332, 1284, 1208, 1151, 1115, 1085, 1047, 1014, 978; ^1^H NMR (400 MHz, DMSO-d_6_) *δ* 7.46 (d, *J* = 8.6 Hz, 2H, H Ar), 7.41 (d, *J* = 8.6 Hz, 2H, H Ar), 5.03 (q, *J* = 7.2 Hz, 1H, CH), 3.75 (s, 3H, OCH_3_), 2.65 (s, 3H, HetCH_3_), 1.32 (d, *J* = 7.2 Hz, 3H, CH_3_); ^13^C NMR (100 MHz, DMSO-d_6_) *δ* 171.2 (C=O), 160.5 (C=O), 159.0 (C Het), 142.3 (C Het), 137.7 (C Ar), 133.9 (C Ar), 131.3 (CH Ar), 129.6 (CH Ar), 56.3 (CH), 52.4 (OCH_3_), 14.8 (CH_3_), 12.9 (CH_3_ Het); EI-MS *m*/*z* (%): 341 [M+2]^+^ (1), 339 [M]^+^ (2), 308 [M–OCH_3_]^+^ (2), 279 [M–HCO_2_CH_3_]^+^ (6), 212 [M–HetCO]^+^ (34), 252 [M–CH(CH_3_)CO_2_CH_3_]^+^ (33), 99 [HetCO–N_2_]^+^ (100). Analysis calculated for C_14_H_14_ClN_3_O_3_S (Mr = 339.79): C 49.49, H 4.15, N 12.37, S 9.44; found C 49.35, H 4.48, N 12.11, S 9.30%.

Methyl-*N*-(4-methyl-1,2,3-thiadiazole-5-carbonyl)-*N*-(3,5-dichlorophenyl)-(*RS*)-alaninate (**1h**): colorless oil, yield 0.322 g (86%); IR, ν, cm^−1^: 3084, 2950, 2950, 1741 (C=O), 1651 (C=O), 1566, 1494, 1458, 1434, 1401, 1380, 1346, 1285, 1211, 1134, 1106, 1089, 1054, 1013, 976; ^1^H NMR (400 MHz, DMSO-d_6_) *δ* 7.67 (br.s., 1H, CH Ar), 7.54 (br.s., 2H, CH Ar), 5.00 (q, *J* = 7.2, 1H, CH), 3.75 (s, 3H, OCH_3_), 2.63 (s, 3H, CH_3_), 1.38 (d, *J* = 7.2 Hz, 3H, CH_3_); ^13^C NMR (100 MHz, DMSO-d_6_) *δ* 171.1 (C=O), 160.4 (C=O), 158.8 (C Het), 142.1 (C Het), 141.3, 134.5 (CH Ar), 129.1, 128.3 (CH Ar), 56.9 (CH), 52.5 (OCH_3_), 14.7 (CH_3_), 12.8 (CH_3_); EI-MS *m*/*z* (%): 376 [M+2]^+^ (1), 374 [M]^+^ (1), 342 [M–CH_3_OH]^+^ (2), 313 [M+1–HCO_2_CH_3_]^+^ (4), 288 [M+2–CH(CH_3_)CO_2_CH_3_]^+^ (14), 286 [M–CH(CH_3_)CO_2_CH_3_]^+^ (14), 274 (4), 252 (7), 250 [M+4–HetCO]^+^ (9), 248 [M+2–HetCO]^+^ (14), 246 [M–HetCO]^+^ (24), 224 (2), 202 (6), 188 (4), 172 (6), 167 (5), 152 (4), 147 (9), 145 (14), 124 (3), 109 (7), 101 (5), 100 (6), 99 [HetCO–N_2_]^+^ (100). Analysis calculated for C_14_H_13_Cl_2_N_3_O_3_S (Mr = 374.24): C 44.93, H 3.50, N 11.23, S 8.57; found C 44.75, H 3.58, N 11.02, S 8.36%.

Methyl-*N*-(4-methyl-1,2,3-thiadiazole-5-carbonyl)-*N*-(3-methyl-4-chlorophenyl)-(*RS*)-alaninate (**1i**) (isomers ration = 3:1): white powder, yield 0.265 g (75%); m.p. 98–100 °C; IR, ν, cm^−1^: 2985, 1739 (C=O), 1650 (C=O), 1572, 1490, 1448, 1353, 1266, 1209, 1195, 1113, 1013, 967; ^1^H NMR (400 MHz, DMSO-d_6_) *δ* 7.60–7.55 (m, 1H, CH Ar minor+major), 7.48–7.77 (m, 1H, CH Ar min+maj), 7.35–7.31 (m, 1H, CH Ar min+maj), 4.87 (q, *J* = 7.3 Hz, 1H, CH maj), 4.65 (q, *J* = 7.0 Hz, 1H, CH min), 3.75 (s, 3H, OCH_3_ maj), 3.73 (s, 3H, OCH_3_ min), 2.76 (3H, s, CH_3_ maj), 2.25 (3H, s, CH_3_), 1.47 (d, *J* = 7.1 Hz, 3H, CH_3_ min), 1.25 (d, *J* = 7.3 Hz, 3H, CH_3_ maj); ^13^C NMR (100 MHz, DMSO-d_6_) *δ* 171.1 (C=O maj), 170.88 (C=O min), 161.3 (C=O min), 161.2 (C=O maj), 160.2 (C Het min), 160.1 (C Het maj), 140.7 (C Het maj), 140.6 (C Het min), 138.9 (C Ar maj+min), 136.1 (C Ar maj), 135.6 (C Ar min), 135.2 (C Ar maj), 134.9 (C Ar min), 130.9 (CH Ar maj), 130.6 (CH Ar min), 129.5 (CH Ar maj), 129.1 (CH Ar min), 128.4 (CH Ar min), 128.3 (CH Ar maj), 58.8 (CH min), 56.5 (CH maj), 52.4 (OCH_3_ min), 52.3 (OCH_3_ maj), 15.5(CH_3_ maj), 15.3 (CH_3_ min), 15.0 (CH_3_ min), 14.0 (CH_3_ maj), 13.5 (CH_3_ Het maj), 13.4 (CH_3_ Het min); EI-MS *m*/*z* (%): 355 [M+2]^+^ (1), 353 [M]^+^ (1), 322 [M–OCH_3_]^+^ (3), 310 (1), 295 [M+2–HCO_2_CH_3_]^+^ (3), 290 (8), 266 [M–CH(CH_3_)CO_2_CH_3_]^+^ (14), 254 (9), 238 (16), 230 (12), 226 [M–HetCO]^+^ (40), 196 (2), 182 (5), 168 (4), 154 (4), 152 (9), 132 (9), 117 (16), 99 [HetCO–N_2_]^+^ (100). Analysis calculated for C_15_H_16_ClN_3_O_3_S (Mr = 353.82): C 50.92, H 4.56, N 11.88, S 9.06; found C 50.69, H 4.63, N 11.73, S 8.86%.

Methyl-*N*-(4-methyl-1,2,3-thiadiazole-5-carbonyl)-*N*-(3-methoxylphenyl)-(*RS*)-alaninate (**1j**): dark yellow oil, yield 0.268 g (80%); IR, ν, cm^−1^: 2996, 2950, 2839, 1742 (C=O), 1645 (C=O), 1599, 1488, 1452, 1382, 1328, 1286, 1206, 1167, 1108, 1088, 1038, 975; ^1^H NMR (400 MHz, DMSO-d_6_) *δ* 7.30 (dd, *J* = 8.4, 8.0 Hz, 1H, CH Ar), 6.98–6.93 (m, 3H, CH Ar), 5.01 (q, *J* = 7.2, 1H, CH), 3.74 (s, 3H, OCH_3_), 3.72 (s, 3H, OCH_3_), 2.67 (s, 3H, CH_3_), 1.34 (d, *J* = 7.2 Hz, 3H, CH_3_); ^13^C NMR (100 MHz, DMSO-d_6_) *δ* 171.2 (C=O), 160.4 (C=O), 159.8, 159.4, 142.2, 139.8, 130.4 (CH Ar), 121.4, 115.3, 115.0, 56.4 (CH), 55.4 (OCH_3_), 52.3 (OCH_3_), 14.7 (CH_3_), 13.1 (CH_3_); EI-MS *m*/*z* (%): 335 [M]^+^ (1), 307 [M–N_2_]^+^ (26), 273 (13), 248 [M–CH(CH_3_)CO_2_CH_3_]^+^ (31), 214 (21), 208 [M–HetCO]^+^ (27), 149 (14), 134 (28), 122 (12), 107 (21), 99 [HetCO–N_2_]^+^ (100). Analysis calculated for C_15_H_17_N_3_O_4_S (Mr = 335.38): C 53.72, H 5.11, N 12.53, S 9.56; found C 53.79, H 4.96, N 12.38, S 9.34%.

Methyl-*N*-(4-methyl-1,2,3-thiadiazole-5-carbonyl)-*N*-(4-methoxylphenyl)-(*RS*)-alaninate (**1k**): yellow oil, yield 0.288 g (86%); IR, ν, cm^−1^: 2996, 2952, 2841, 1743 (C=O), 1657 (C=O), 1606, 1579, 1512, 1458, 1385, 1335, 1298, 1247, 1182, 1152, 1114, 1089, 1029, 979; ^1^H NMR (400 MHz, DMSO-d_6_) *δ* 7.29 (d, *J* = 8.8 Hz, 2H, CH Ar), 6.94 (d, *J* = 8.8 Hz, 2H, CH Ar), 5.02 (q, *J* = 7.2 Hz, 1H, CH), 3.75 (s, 3H, OCH_3_), 3.73 (s, 3H, OCH_3_), 2.70 (s, 3H, CH_3_), 1.29 (d, *J* = 7.2 Hz, 3H, CH_3_); ^13^C NMR (100 MHz, DMSO-d_6_) *δ* 171.4 (C=O), 160.7 (C=O), 159.9 (C Het), 159.7 (C Ar), 142.0 (C Het), 131.0 (CH Ar), 130.9 (C Ar), 114.8 (CH Ar), 56.1 (CH), 55.4 (OCH_3_), 52.3 (OCH_3_), 14.9 (CH_3_), 13.3 (CH_3_); EI-MS *m*/*z* (%): 337 [M+2]^+^ (4), 336 [M+1]^+^ (14), 335 [M]^+^ (49), 304 [M–OCH_3_]^+^ (6), 292 (5), 275 [M–HCO_2_CH_3_]^+^ (9), 264 (4), 248 [M–CH(CH_3_)CO_2_CH_3_]^+^ (99), 236 (8), 220 (16), 208 [M–HetCO]^+^ (96), 186 (10), 176 (8), 162 (17), 149 (49), 134 [M–Het]^+^ (99), 122 (24), 99 [HetCO–N_2_]^+^ (100). Analysis calculated for C_15_H_17_N_3_O_4_S (Mr = 335.38): C 53.72, H 5.11, N 12.53, S 9.56; found C 53.66, H 5.24, N 12.58, S 9.31%.

Methyl-*N*-(4-methyl-1,2,3-thiadiazole-5-carbonyl)-*N*-(3-trifluoromethylphenyl)-(*RS*)-alaninate (**1l**): white crystals, yield 0.358 g (96%); m.p. 71–72 °C; IR, ν, cm^−1^: 2960, 1741 (C=O), 1646 (C=O), 1587, 1487, 1435, 1391, 1353, 1327, 1301, 1255, 1212, 1168, 1140, 1117, 1092, 1069, 1050, 1001, 978; ^1^H NMR (400 MHz, DMSO-d_6_) *δ* 7.80 (br.s., 1H, CH Ar), 7.69–7.55 (m, 2H, CH Ar), 7.62 (dd, *J* = 7.8, 7.6 Hz, 1H, CH Ar), 5.08 (q, *J* = 7.1 Hz, 1H, CH), 3.76 (s, 3H, OCH_3_), 2.61 (s, 3H, CH_3_), 1.35 (d, *J* = 7.1 Hz, 3H, CH_3_); ^13^C NMR (100 MHz, DMSO-d_6_) *δ* 171.3 (C=O), 160.6 (C=O), 158.5 (C Het), 142.4 (C Het), 139.7 (C Ar), 133.4 (CH Ar), 130.9 (CH Ar), 130.0 (q, *J_C-F_* = 32.4 Hz, C Ar), 123.4 (q, *J_C-F_* = 272.4 Hz, CF_3_), 126.3 (d, *J_C-F_* = 3.4 Hz, CH Ar), 125.9 (d, *J_C-F_* = 2.8 Hz, CH Ar), 56.5 (CH), 52.5 (OCH_3_), 14.8 (CH_3_), 12.7 (CH_3_); EI-MS *m*/*z* (%): 374 [M+1]^+^ (1), 342 [M–CH_3_OH]^+^ (1), 313 [M–HCO_2_CH_3_]^+^ (4), 302 (1), 286 [M–CH(CH_3_)CO_2_CH_3_]^+^ (30), 274 (4), 252 (4), 246 [M–HetCO]^+^ (25), 224 (2), 216 (1), 202 (11), 186 (5), 172 (7), 159 (1), 145 (19), 125 (3), 99 [HetCO–N_2_]^+^ (100). Analysis calculated for C_15_H_14_F_3_N_3_O_3_S (Mr = 373.35): C 48.26, H 3.78, N 11.26, S 8.59; found C 48.15, H 3.60, N 10.98, S 8.43%.

Methyl-*N*-(3,4-dichloroisothiazole-5-carbonyl)-*N*-phenyl)-(*RS*)-alaninate (**2a**): light yellow oil, yield 0.255 g (71%); IR, ν, cm^−1^: 3063, 2994, 2952, 1745 (C=O), 1652 (C=O), 1594, 1493, 1454, 1397, 1294, 1208, 1113, 1085, 1026, 951; ^1^H NMR (400 MHz, DMSO-d_6_) *δ* 7.38–7.43 (m, 5H, CH Ar), 5.01 (q, *J* = 7.2, 1H, CH), 3.74 (s, 3H, OCH_3_), 1.31 (d, *J* = 7.2, 3H, CH_3_); ^13^C NMR (100 MHz, DMSO-d_6_) *δ* 170.9 (C=O), 158.7 (C=O), 155.5 (C Het), 146.2 (C Het), 138.1 (C Ar), 129.4 (CH Ar), 129.4 (CH Ar), 129.3 (CH Ar), 120.5 (C Het), 56.2 (CH), 52.3 (OCH_3_), 14.7 (CH_3_); EI-MS *m*/*z* (%): 362 [M+4]^+^ (3), 360 [M+2]^+^ (12), 358 [M]^+^ (17), 329 [M+2–OCH_3_]^+^ (1), 327 [M–OCH_3_]^+^ (2), 303 [M+4–CO_2_CH_3_]^+^ (13), 301 [M+2–CO_2_CH_3_]^+^ (68), 299 [M–CO_2_CH_3_]^+^ (95), 184 (14), 183 [HetCO+2]^+^ (5), 182 (70), 181 (9), 180 [HetCO]^+^ (100), 118 (16), 104 (33), 77 (62). Analysis calculated for C_14_H_12_Cl_2_N_2_O_3_S (Mr = 359.22): C 46.81, H 3.37, N 7.80, S 8.92; found C 46.59, H 3.52, N 7.73, S 8.90%.

Methyl-*N*-(3,4-dichloroisothiazole-5-carbonyl)-*N*-(3-methylphenyl)-(*RS*)-alaninate (**2b**): colorless oil, yield 0.336 g (90%). IR, ν, cm^−1^: 2992, 2951, 1743 (C=O), 1650 (C=O), 1603, 1586, 1488, 1455, 1385, 1293, 1212, 1192, 1140, 1110, 1085, 1050, 1050, 1002, 977; ^1^H NMR (400 MHz, DMSO-d_6_) *δ* 7.29–7.19 (4H, m, H Ar), 4.97 (q, *J* = 7.2 Hz, 1H, CH), 3.73 (s, 3H, OCH_3_), 2.27 (s, 3H, CH_3_), 1.33 (d, *J* = 7.2 Hz, 3H, CH_3_). ^13^C NMR (100 MHz, DMSO-d_6_) *δ* 170.9 (C=O), 158.6 (C=O), 155.3 (C Het), 146.3 (C Het), 139.1 (CH Ar), 138.1 (C Ar), 130.1, 129.7, 129.2, 126.3 (CH Ar), 120.9 (C Het), 56.4 (CH), 52.3 (OCH_3_), 20.7 (CH_3_), 14.7 (CH_3_); EI-MS *m*/*z* (%): 376 [M+4]^+^ (3), 374 [M+2]^+^ (15), 372 [M]^+^ (20), 341 [M–OCH_3_]^+^ (2), 317 [M+4–CO_2_CH_3_]^+^ (15), 315 [M+2–CO_2_CH_3_]^+^ (74), 313 [M–CO_2_CH_3_]^+^ (100), 305 (1), 277 (2), 265 (1), 251 (1), 237 (1), 184 [HetCO+4]^+^ (12), 182 [HetCO+2]^+^ (62), 180 [HetCO]^+^ (89), 164 (1), 148 (1), 133 (13), 132 (19), 118 (31), 104 (3); Analysis calculated for C_15_H_14_Cl_2_N_2_O_3_S (Mr = 373.25): C 48.27, H 3.78, N 7.51, S 8.59; found C 48.49, H 3.91, N 7.78, S 8.48%.

Methyl-*N*-(3,4-dichloroisothiazole-5-carbonyl)-*N*-(*p*-tolyl)-(*RS*)-alaninate (**2c**): light yellow oil, yield 0.280 g (75%). IR, ν, cm^−1^: 2993, 2951, 1744 (C=O), 1650 (C=O), 1510, 1391, 1293, 1206, 1110, 1084, 1045, 1022, 951; ^1^H NMR (400 MHz, DMSO-d_6_) *δ* 7.31 (d, *J* =8.1 Hz, 2H, CH Ar), 7.19 (d, *J* =8.1 Hz, 2H, CH Ar), 5.01 (1H, q, *J* = 7.1, CH), 3.73 (3H, s, OCH_3_), 2.28 (3H, s, CH_3_), 1.30 (3H, d, *J* = 7.2, CH_3_); ^13^C NMR (100 MHz, DMSO-d_6_) *δ* 171.0 (C=O), 158.7 (C=O), 155.5 (C Het), 146.3 (C Het), 139.2 (C Ar), 135.4 (C Ar), 129.9 (CH Ar), 129.2 (CH Ar), 120.7 (C Het), 56.1 (CH), 52.3 (OCH_3_), 20.6 (Ar CH_3_), 14.7 (CH_3_); EI-MS *m*/*z* (%): 376 [M+4]^+^ (4), 374 [M+2]^+^ (18), 372 [M]^+^ (27), 343 [M+2–OCH_3_]^+^ (1), 341 [M–OCH_3_]^+^ (2), 317 [M+4–CO_2_CH_3_]^+^ (15), 315 [M+2–CO_2_CH_3_]^+^ (70), 313 [M–CO_2_CH_3_]^+^ (97), 184 [HetCO+4]^+^ (13), 183 [HetCHO+2]^+^ (4), 182 [HetCO+2]^+^ (67), 181 [HetCHO]^+^ (8), 180 [HetCO]^+^ (100), 118 (35), 91 (55). Analysis calculated for C_15_H_14_Cl_2_N_2_O_3_S (Mr = 373.25): C 48.27, H 3.78, N 7.51, S 8.59; found C 48.38, H 3.61, N 7.44, S 8.78%.

Methyl-*N*-(3,4-dichloroisothiazole-5-carbonyl)-*N*-(2,6-dimethylphenyl)-(*RS*)-alaninate (**2d**): white powder, yield 0.279 g (72%); m.p. 120–121 °C; IR, ν, cm^−1^: 2987, 2957, 1749 (C=O), 1645 (C=O), 1453, 1385, 1336, 1308, 1267, 1207, 1180, 1121, 1085, 979; ^1^H NMR (400 MHz, DMSO-d_6_) *δ* 7.37 (t, *J* = 7.6 Hz, 1H, H Ar), 7.25 (dd, *J* = 7.2, 6.8 Hz, 2H, H Ar), 4.66 (q, *J* = 7.3 Hz, 1H, CH), 3.73 (s, 3H, OCH_3_), 2.31 (s, 3H, CH_3_), 2.21 (s, 3H, CH_3_), 1.10 (d, *J* = 7.3 Hz, 3H, CH_3_); ^13^C NMR (100 MHz, DMSO-d_6_) *δ* 171.2 (C=O), 158.1 (C=O), 151.1 (C Het), 147.8 (C Het), 139.3 (C Ar), 138.9 (C Ar), 135.0 (C Ar), 130.6 (CH Ar), 129.7 (CH Ar), 129.5 (CH Ar), 125.0 (C Het), 56.2 (CH), 52.1 (OCH_3_), 18.2 (CH_3_), 17.9 (CH_3_), 14.5 (CH_3_); EI-MS *m*/*z* (%): 390 [M+4]^+^ (3), 389 [M+3]^+^ (3), 388 [M+2]^+^ (15), 387 [M+1]^+^ (5), 386 [M]^+^ (23), 354 (4), 331 [M+4–CO_2_CH_3_]^+^ (14), 330 [M+4–HCO_2_CH_3_]^+^ (12), 329 [M+4–CO_2_CH_3_]^+^ (68), 328 [M+2–HCO_2_CH_3_]^+^ (19), 327 [M–CO_2_CH_3_]^+^ (100), 326 [M–HCO_2_CH_3_]^+^ (1), 267 (12), 265 (18), 239 (19), 237 (28), 206 [M–HetCO]^+^ (5), 184 [HetCO+4]^+^ (13), 182 [HetCO+2]^+^ (68), 180 [HetCO]^+^ (97), 147 (13), 146 (37), 132 (69), 131 (15), 117 (31), 105 (38). Analysis calculated for C_16_H_16_Cl_2_N_2_O_3_S (Mr = 387.28): C 49.62, H 4.16, N 7.23, S 8.28; found C 49.45, H 4.39, N 7.17, S 8.35%.

Methyl-*N*-(3,4-dichloroisothiazole-5-carbonyl)-*N*-(2,4,6-trimethylphenyl)-(*RS*)-alaninate (**2e**): white powder, yield 0.309 g (77%); m.p. 120–121 °C; IR, ν, cm^−1^: 2989, 2947, 1746 (C=O), 1644 (C=O), 1481, 1452, 1434, 1377, 1338, 1303, 1303, 1259, 119, 1167, 1110, 1082, 1035, 980; ^1^H NMR (400 MHz, DMSO-d_6_) *δ* 7.07 (d, *J* = 5.4 Hz, 2H, CH Ar), 4.62 (q, *J* = 7.3 Hz, 1H, CH), 3.72 (s, 3H, OCH_3_), 2.30 (s, 3H, CH_3_), 2.26 (s, 3H, CH_3_), 2.15 (s, 3H, CH_3_), 1.08 (d, *J* = 7.3 Hz, 3H, CH_3_); ^13^C NMR (100 MHz, DMSO-d_6_) *δ* 171.3 (C=O), 158.2 (C=O), 151.0 (C Het), 147.8 (C Het), 140.4 (C Ar), 138.9 (C Ar), 138.6 (C Ar), 132.4 (C Ar), 130.3 (CH Ar), 130.1 (CH Ar), 125.0 (C Het), 56.3 (CH), 52.1 (OCH_3_), 20.6 (CH_3_ Ar), 18.1 (CH_3_ Ar), 17.8 (CH_3_ Ar), 14.5 (CH_3_); EI-MS *m*/*z* (%): 404 [M+4]^+^ (9), 403 [M+3]^+^ (8), 402 [M+2]^+^ (39), 401 [M+1]^+^ (13), 400 [M]^+^ (54), 370 [M+2–CH_3_OH]^+^ (4), 369 [M–OCH_3_]^+^ (5), 368 [M–CH_3_OH]^+^ (5), 345 [M+4–CO_2_CH_3_]^+^ (14), 344 (14), 343 [M+2–CO_2_CH_3_]^+^ (67), 342 (20), 341 [M–CO_2_CH_3_]^+^ (100), 267 (43), 265 (62), 239 (42), 237 (60), 220 [M–HetCO]^+^ (6), 184 [HetCO+4]^+^ (9), 182 [HetCO+2]^+^ (44), 180 [HetCO]^+^ (64), 161 (40), 160 (37), 146 (79), 131 (37), 104 (7), 103 (8). Analysis calculated for C_17_H_18_Cl_2_N_2_O_3_S (Mr = 401.30): C 50.88, H 4.52, N 6.98, S 7.99; found C 50.96, H 4.75, N 7.06, S 8.09%.

Methyl-*N*-(3,4-dichloroisothiazole-5-carbonyl)-*N*-(3-chlorophenyl)-(*RS*)-alaninate (**2f**): colorless oil, yield 0.382 g (97%). IR, ν, cm^−1^: 2993, 2951, 1743 (C=O), 1653 (C=O), 1587, 1509, 1475, 1456, 1434, 1394, 1291, 1249, 1207, 1119, 1081, 1048, 1001, 975; ^1^H NMR (400 MHz, DMSO-d_6_) *δ* 7.60 (s, 1H, CH Ar), 7.46–7.39 (m, 3H, CH Ar), 5.02 (q, *J* = 7.2 Hz, 1H, CH), 3.74 (s, 3H, OCH_3_), 1.34 (d, *J* = 7.2 Hz, CH_3_); ^13^C NMR (100 MHz, DMSO-d_6_) *δ* 170.9 (C=O), 158.8 (C=O), 155.6 (C Het), 146.2 (C Het), 139.6, 133.2, 130.9, 129.4, 129.3, 128.0, 120.2 (C Het), 56.3 (CH), 52.4 (OCH_3_), 14.7 (CH_3_); EI-MS *m*/*z* (%): 396 [M+4]^+^ (5), 394 [M+2]^+^ (13), 392 [M]^+^ (13), 363 [M+2–OCH_3_]^+^ (2), 337 [M+4–CO_2_CH_3_]^+^ (24), 335 [M+2–CO_2_CH_3_]^+^ (66), 333 [M–CO_2_CH_3_]^+^ (64), 212 [M–HetCO]^+^ (6), 184 [HetCO+4]^+^ (14), 182 [HetCO+2]^+^ (69), 180 [HetCO]^+^ (100), 154 (3), 152 (7), 138 (14), 113 (7), 111 (21). Analysis calculated for C_14_H_11_Cl_3_N_2_O_3_S (Mr = 393.66): C 42.72, H 2.82, N 7.12, S 8.14; found C 42.63, H 2.68, N 7.31, S 8.28%.

Methyl-*N*-(3,4-dichloroisothiazole-5-carbonyl)-*N*-(p-chlorophenyl)-(*RS*)-alaninate (**2g**): light yellow oil, yield 0.358 g (91%). IR, ν, cm^−1^: 2993, 2952, 1723 (C=O), 1651 (C=O), 1489, 1455, 1394, 1290, 1207, 1113, 1085, 1046, 1016, 952; ^1^H NMR (400 MHz, DMSO-d_6_) *δ* 7.47 (s, 4H, H Ar), 5.01 (q, *J* = 7.2 Hz, 1H, CH), 3.73 (s, 3H, OCH_3_), 1.31 (d, *J* = 7.2 Hz, 3H, CH_3_); ^13^C NMR (100 MHz, DMSO-d_6_) *δ* 170.9 (C=O), 158.8 (C=O), 155.6 (C Het), 146.2 (C Het), 137.0 (C Ar), 133.9 (C Ar), 131.1 (CH Ar), 129.4 (CH Ar), 120.3 (C Het), 56.1 (CH), 52.3 (OCH_3_), 14.7 (CH_3_); EI-MS *m*/*z* (%): 396 [M+4]^+^ (5), 394 [M+2]^+^ (14), 392 [M]^+^ (13), 363 [M+2–OCH_3_]^+^ (1), 361 [M–OCH_3_]^+^ (1), 337 [M+4–CO_2_CH_3_]^+^ (19), 335 [M+2–CO_2_CH_3_]^+^ (54), 333 [M–CO_2_CH_3_]^+^ (54), 183 [HetCHO+2]^+^ (4), 182 [HetCO+2]^+^ (68), 181 [HetCHO]^+^ (8), 180 [HetCO]^+^ (100), 118 (2), 111 (24). Analysis calculated for C_14_H_11_Cl_3_N_2_O_3_S (Mr = 393.66): C 42.72, H 2.82, N 7.12, S 8.14; found C 42.55, H 2.94, N 7.22, S 7.95%.

Methyl-*N*-(3,4-dichloroisothiazole-5-carbonyl)-*N*-(3,5-dichlorophenyl)-(*RS*)-alaninate (**2h**): colorless oil, yield 0.253 g (59%). IR, ν, cm^−1^: 3077, 2993, 2952, 1743 (C=O), 1658 (C=O), 1580, 1567, 1508, 1435, 1393, 1288, 1209, 1131, 1104, 1084, 1054, 998; ^1^H NMR (400 MHz, DMSO-d_6_) *δ* 7.66 (br. s., 1H, CH Ar), 7.59 (br.s., 2H, H Ar), 5.00 (q, *J* = 7.1 Hz, 1H, CH), 3.74 (s, 3H, OCH_3_), 1.36 (d, *J* = 7.1 Hz, 3H, CH_3_); ^13^C NMR (100 MHz, DMSO-d_6_) *δ* 170.8 (C=O), 158.8 (C=O), 155.5 (C Het), 146.2 (C Het), 140.5 (C Ar), 134.1 (CH Ar), 129.1 (C Ar), 128.2 (C Ar), 120.0 (C Het), 56.6 (CH), 52.4 (OCH_3_), 14.6 (CH_3_); EI-MS *m*/*z* (%): 432 [M+4]^+^ (1), 431 [M+3]^+^ (1), 430 [M+2]^+^ (6), 429 [M+1]^+^ (2), 428 [M]^+^ (11), 426 (8), 397 (2), 246 (4), 184 [HetCO+4]^+^ (14), 183 [HetCHO+2]^+^ (5), 182 [HetCO+2]^+^ (69), 180 [HetCO]^+^ (100), 145 (12), 117 (4), 109 (6); Analysis calculated for C_14_H_10_Cl_4_N_2_O_3_S (Mr = 428.11): C 39.28, H 2.35, N 6.54, S 7.49; found C 39.39, H 2.58, N 6.46, S 7.33%.

Methyl-*N*-(2-methyl-3-chloroisothiazole-5-carbonyl)-*N*-(3,5-dichlorophenyl)-(*RS*)-alaninate (**2i**) (isomers ration 4:1): colorless oil, yield 0.273 g (67%). IR, ν, cm^−1^: 2980, 2960, 1736 (C=O), 16,501 (C=O), 1588, 1568, 1497, 1431, 1382, 1342, 1312, 1267, 1214, 1198, 1177, 1115, 1088, 1050, 1014, 978; ^1^H NMR (400 MHz, DMSO-d_6_) *δ* 7.58–7.49 (m, 2H, CH Ar min+maj), 7.34–7.30 (m, 1H, CH Ar min+maj), 4.84–4.79 (m, 1H, CH maj), 4.71–4.64 (m, 1H, CH min), 3.75 (s, 3H, OCH_3_ maj), 3.73 (s, 4H, OCH_3_ min), 2.33 (s, 3H, CH_3_ min+maj), 1.48 (d, *J* = 6.5 Hz, 3H, CH_3_ min), 1.28 (d, *J* = 6.8 Hz, 3H, CH_3_ maj); ^13^C NMR (100 MHz, DMSO-d_6_) *δ* 170.8 (C=O maj), 170.6 (C=O min), 158.4 (C=O min), 158.2 (C=O maj), 153.5 (C Het min), 153.4 (C Het maj), 147.2 (C Het min), 147.2 (C Het maj), 140.1 (min), 138.2 (maj), 136.1 (maj), 135.7 (min), 135.1 (maj), 134.9 (min), 130.9 (maj), 130.6 (min), 129.5 (maj), 129.2 (min), 128.1 (min), 128.0 (maj), 122.7 (C Het min), 122.6 (C Het maj), 58.9 (CH min), 56.8 (CH maj), 52.4 (OCH_3_ maj), 52.3 (OCH_3_ min), 15.8 (min), 15.5 (maj), 14.9 (min), 13.8 (maj); EI-MS *m*/*z* (%): 410 [M+4]^+^ (2), 408 [M+2]^+^ (7). 406 [M]^+^ (6), 374 [M–CH_3_OH]^+^ (2), 351 [M–CH_3_OH]^+^ (20), 349 [M+2–CO_2_CH_3_]^+^ (54), 347 [M–CO_2_CH_3_]^+^ (55), 226 [M–HetCO]^+^ (7), 184 [HetCO+4]^+^ (13), 182 [HetCO+2]^+^ (67), 180 [HetCO]^+^ (100), 166 (6), 152 (11), 131 (6), 117 (24), 99 (5), 89 (18); Analysis calculated for C_15_H_13_Cl_3_N_2_O_3_S (Mr = 407.69): C 44.19, H 3.21, N 6.87, S 7.86; found C 44.27, H 3.45, N 6.62, S 7.94%.

Methyl-*N*-(3,4-dichloroisothiazole-5-carbonyl)-*N*-(3-methoxyphenyl)-(*RS*)-alaninate (**2j**): colorless oil, yield 0.327 g (84%). IR, ν, cm^−1^: 2998, 2950, 2839, 1743 (C=O), 1653 (C=O), 1599, 1587, 1488, 1453, 1434, 1395, 1293, 1204, 1176, 1109, 1085, 1038, 996; ^1^H NMR (400 MHz, DMSO-d_6_) *δ* 7.29 (dd, *J* = 8.1, 8.1 Hz, 1H, CH Ar), 7.05 (s, 1H, CH Ar), 7.00 (d, *J* = 7.8, Hz, CH Ar), 6.95 (dd, *J* = 8.3, 2.1 Hz, 1H, CH Ar), 5.01 (q, *J* = 7.2 Hz, 1H, CH), 3.74 (s, 3H, OCH_3_), 3.72 (s, 3H, OCH_3_), 1.34 (d, *J* = 7.2 Hz, 3H, CH_3_); ^13^C NMR (100 MHz, DMSO-d_6_) *δ* 170.9 (C=O), 159.6 (C Ar), 158.7 (C=O), 155.5 (C Het), 146.2 (C Het), 139.2 (C Ar), 130.2 (CH Ar), 121.3 (CH Ar), 120.7 (C Het), 115.2 (CH Ar), 115.1 (CH Ar), 56.2 (CH), 55.3 (ArOCH_3_), 52.3 (OCH_3_), 14.7 (CH_3_); MS (EI, 70 eV), *m*/*z* (I_rel_,%): 392 (2), 391 (2), 390 (10), 389 (3), 388 (14), 353 (17), 331 (69), 330 (17), 329 (98), 295 (3), 293 (8), 208 (9), 184 (13), 182 (69), 180 (100), 148 (13), 134 (23), 107 (22); EI-MS *m*/*z* (%): 392 [M+4]^+^ (2), 391 [M+3]^+^ (1), 390 [M+2]^+^ (10), 389 [M+1]^+^ (3), 388 [M]^+^ (14), 353 (17), 329 [M–CO_2_CH_3_]^+^ (98), 321 (22), 293 (8), 208 [M–HetCO]^+^ (9), 184 [HetCO+4]^+^ (13), 183 (4), 182 [HetCO+2]^+^ (69), 181 (7), 180 [HetCO]^+^ (100), 148 (13), 134 (23), 117 (4), 107 (22). Analysis calculated for C_15_H_14_Cl_2_N_2_O_4_S (Mr = 389.25): C 46.29, H 3.63, N 7.20, S 8.24; found C 46.03, H 3.48, N 7.09, S 7.97%.

Methyl-*N*-(3,4-dichloroisothiazole-5-carbonyl)-*N*-(p-methoxyphenyl)-(*RS*)-alaninate (**2k**): colorless oil, yield 0.319 g (82%). IR, ν, cm^−1^: 2922, 2952, 2840, 1743 (C=O), 1649 (C=O), 1606, 1580, 1508, 1456, 1388, 1292, 1247, 1206, 1180, 1170, 1108, 1085, 1027, 981; ^1^H NMR (400 MHz, DMSO-d_6_) *δ* 7.36 (d, *J* = 8.7 Hz, 2H, CH Ar), 6.94 (d, *J* = 8.7 Hz, 2H, CH Ar), 5.02 (q, *J* = 7.2 Hz, 1H, CH), 3.74 (s, 3H, OCH_3_), 3.73 (s, 3H, OCH_3_), 1.29 (d, *J* = 7.2 Hz, 3H, CH_3_); ^13^C NMR (100 MHz, DMSO-d_6_) *δ* 171.0 (C=O), 159.7 (C=O), 158.9 (C Ar), 155.2 (C Het), 146.4 (C Het), 130.9 (CH Ar), 130.2 (C Ar), 120.9 (C Het), 114.5 (CH Ar), 55.9, 55.4, 52.2, 14.7 (CH_3_); EI-MS *m*/*z* (%): 392 [M+4]^+^ (8), 391 [M+3]^+^ (7), 390 [M+2]^+^ (37), 389 [M+1]^+^ (11), 388 [M]^+^ (53), 357 [M–OCH_3_]^+^ (3), 356 [M–CH_3_OH]^+^ (3), 331 [M+2–CO_2_CH_3_]^+^ (72), 330 [M+2–HCO_2_CH_3_]^+^ (18), 329 [M–CO_2_CH_3_]^+^ (100), 265 (9), 237 (9), 208 [M–HetCO]^+^ (18), 184 [HetCO+4]^+^ (10), 180 [HetC**^·^**O]^+^ (79), 149 (49), 134 (73), 122 (11), 107 (11); Analysis calculated for C_15_H_14_Cl_2_N_2_O_4_S (Mr = 389.25): C 46.29, H 3.63, N 7.20, S 8.24; found C 46.14, H 3.55, N 7.04, S 8.01%.

Methyl-*N*-(3,4-dichloroisothiazole-5-carbonyl)-*N*-(3-trifluoromethylphenyl)-(*RS*)-alaninate (**2l**): beige powder, yield 0.261 g (61%); m.p. 79–80 °C; IR, ν, cm^−1^: 3004, 2955, 1738 (C=O), 1651 (C=O), 1612, 1587, 1488, 1462, 1441, 1383, 1325, 1300, 1255, 1205, 1205, 1166, 1116, 1088, 1067, 1045, 983; ^1^H NMR (400 MHz, DMSO-d_6_) *δ* 7.87 (br. s, 1H, CH Ar), 7.77–7.73 (m, 2H, H Ar), 7.63 (t, *J* = 7.7, 7.7 Hz, 1H, H Ar), 5.08 (q, *J* = 7.0 Hz, 1H, CH), 3.75 (s, 3H, OCH_3_), 1.35 (d, *J* = 7.0 Hz, 3H, CH_3_); ^13^C NMR (100 MHz, DMSO-d_6_) *δ* 170.9 (C=O), 159.0 (C=O), 155.8 (C Het), 146.1 (C Het), 138.9 (C Ar), 133.3 (CH Ar), 130.6 (CH Ar), 129.7 (q, *J_C-F_* = 32.3 Hz, *p*-C Ar), 126.2 (q, *J_C-F_* = 3.8 Hz, CH Ar), 125.9 (q, *J_C-F_* = 3.3 Hz, CH Ar), 123.4 (q, *J_C-F_* = 272.8 Hz, CF_3_), 119.9 (C Het), 56.2 (CH), 52.4 (OCH_3_), 14.7 (CH_3_); EI-MS *m*/*z* (%): 430 [M+4]^+^ (1), 429 [M+3]^+^ (1), 428 [M+2]^+^ (6), 427 [M+1]^+^ (2), 426 [M]^+^ (7), 407 (1), 395 [M–OCH_3_]^+^ (1), 379 (2), 369 [M+2–CO_2_CH_3_]^+^ (38), 368 [M+2–HCO_2_CH_3_]^+^ (10), 367 [M–CO_2_CH_3_]^+^ (56), 246 (7), 182 [HetCO+2]^+^ (69), 181 [HetCHO]^+^ (7), 180 [HetCO]^+^ (100), 145 (31), 125 (4), 110 (2). Analysis calculated for C_15_H_11_Cl_2_F_3_N_2_O_3_S (Mr = 427.22): C 42.17, H 2.60, N 6.56, S 7.50; found C 41.91, H 2.78, N 6.32, S 7.37%.

### 3.2. X-ray Structure Determination of ***1f***

Crystal data for C_14_H_14_ClN_3_O_3_S (M = 339.79 g/mol): orthorhombic, space group Pbca, a = 9.5344(12) Å, b = 13.538(2) Å, c = 23.985(3) Å, V = 3095.9(8) Å3, Z = 8, μ(MoKα) = 0.397 mm^−1^, Dcalc = 1.458 g/cm^3^, 11,607 reflections measured (7.3° ≤ 2Θ ≤ 60.976°), 4160 unique (Rint = 0.0662, Rsigma = 0.0850) were used in all calculations. The final R1 = 0.0766, wR2 = 0.2065 (I > 2σ(I)) and R1 = 0.1513, wR2 = 0.2655 (all data). Largest diff. peak/hole 0.30/−0.35 ēÅ^−3^.

The experiment was accomplished on the automated X-ray diffractometer «Xcalibur 3» with CCD detector following standard procedures (MoKα-irradiation, graphite monochromator, ω-scans with 1o step at T = 295(2) K). Empirical absorption correction was applied. The structure was solved using the intrinsic phases in ShelXT program [44] and refined by ShelXL [45] using full-matrix least-squared method for non-hydrogen atoms. The H-atoms were placed in the calculated positions and were refined in isotropic approximation. The solution and refinement of the structures were accomplished with the Olex program package [46]. Detailed parameters are shown in Table 5 [47]. The XRD data were deposited in the Cambridge Structural Database with number CCDC 2212217. These data can be requested free of charge via www.ccdc.cam.ac.uk.

### 3.3. Fungicidal Activity In Vitro

The fungicidal activity of all the compounds was tested *in vitro* on *Alternaria brassicicola* (Schwein.) Wiltshire MFP240231, *Alternaria solani* Sorauer MFP601021, *Botrytis cinerea* Pers. MFG 60449, *Colletotrichum coccodes* JS 161-1, *Fusarium solani* (Mart.) Sacc. MFG 70523, *Phytophthora infestans* (Mont.) de Bary, *Plenodomus lingam* (Tode:Fr.) Höhn. MF Br17-044, *Rhizoctonia solani* RCAM01785 and *Sclerotinia sclerotiorum*, using the poisoned food technique. *P. infestans* was isolated in Nankai University (Tianjin, China). *C. coccodes* was purchased from the Russian National Collection of Industrial Microorganisms (Moscow, Russia). Other fungal strains were purchased from the Russian Collection of Agriculture Microorganisms (Saint-Petersburg, Russia).

The solutions of the compounds were prepared at a concentration of 1 mg/mL by dissolving 10 mg of the compound in 1 mL of DMSO, accompanied by an addition of 9 mL of water. A total of 1 mL of the tested solutions were added to Petri dishes containing 9 mL of the heated (60 °C) culture medium and homogeneously mixed in the laminar flow cabinet. Uniform fungal discs (4 mm diameter) were aseptically cut from a 7-day old culture of the test fungus using a sterile cork borer. Discs of mycelia were placed on the center of Petri dishes containing room temperature culture medium. The negative control was prepared with culture medium and DMSO only. Each compound and control treatments were in triplicate; the fungi were incubated at 25 °C (48 h for *R. solani*; 120 h for *A. solani*, *A. brassicicola*, *P. lingam*; 72 h for other fungi). The diameters of the fungal colonies were measured after incubation. Inhibition of the pathogen development was determined according to the formula of Royse and Ries [48]: I (%) = [(C − T)/(C − 4 mm)] × 100, where I (%) is the degree of inhibition of mycelial growth, T (mm) is the mean value of the diameter of the colonies in the presence of a given concentration of each compound and C (mm) is the mean diameter of the colonies in the absence of the compound under the same conditions. All the experiments were carried out in triplicate. The standard deviation was calculated.

### 3.4. Protective Activity of Compounds **1d** and **2d** on Rape Leaves

The in vivo protective activity of compounds **1d** and **2d** against *A. brassicicola* was evaluated at a concentration of 200 μg/mL, according to previously reported procedures [49]. Tiadinil and isotianil were chosen as positive controls. The negative control was sprayed with sterile water containing 0.1% of DMSO. Rape plants were grown in pots to the 5-leaf stage. Rape leaves were cut and sprayed with solution of test compound and placed in 10-mm sterile Petri dishes on 2 pieces of filter paper. Then, 5 mL of sterile water was added to maintain humidity. After 24 h, discs of mycelia were placed on each side of the leaf at an equal distance from the median vein. Inoculated leaves were placed at 25 °C with a daily 16-h light period and 80% humidity in the Binger climate chamber for disease development. After 5 days of inoculation, disease spot diameters were measured and protection efficacy was calculated according to the above formula [48]. All the experiments were carried out in triplicate. The standard deviation was calculated.

## 4. Conclusions

In total, 24 new heterocyclic derivatives of *N*-acyl-*N*-arylalaninates were synthesized. The structures of all target compounds were proven using ^1^H and ^13^C NMR spectroscopy, IR spectroscopy and mass spectrometry. In the series of obtained compounds, methyl *N*-acyl-*N*-arylalaninates exhibiting medium fungicidal activity against *S. sclerotiorum* were identified, two of which were also moderately active against *B. cinerea* and *R. solani*. Compound **1d** was shown to exhibit high protective properties in vivo against *A. brassicicola*, suppressing the development of the disease on rapeseed leaves at a level similar to tiadinil.

## Figures and Tables

**Table 1:** Optimization of reaction for synthesis of compound **3a**.

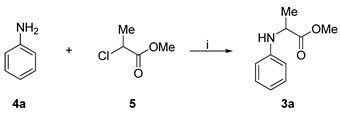					
**Entry**	**Solvent**	**Base**	**Additives**	**Temperature, °C**	**Yield, %**
1	acetonitrile	Na_2_CO_3_	–	80	<1
2	acetonitrile	K_2_CO_3_	–	80	4–5
3	methanol	MeCO_2_Na	–	60	16
4	ethanol	MeCO_2_Na	–	70	35
5	DMF	K_2_CO_3_	–	80	52
6	DMF	K_2_CO_3_	1.0 eq. KI	80	71
7	DMF	K_2_CO_3_	1.5 eq. KI	80	88
8	DMF	K_2_CO_3_	2.0 eq. KI	80	92

**Table 2:** Synthesis route of the target compounds **1a**–**l** and **2a**–**l**.

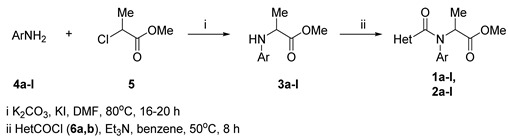						
**Ar**	**For Compounds 3**		**For Compounds 1, 2 and 6**			
			**Het—4-methyl-1,2,3-thiadiazolyl (1a–l, 6a)**		**Het—3,4-dichloro-isothiazolyl (2a–l, 6b)**	
	**Comp.**	**Yield, %**	**Comp.**	**Yield, %**	**Comp.**	**Yield, %**
C_6_H_5_	**3a**	92	**1a**	73	**2a**	71
3-MeC_6_H_4_	**3b**	76	**1b**	74	**2b**	90
4-MeC_6_H_4_	**3c**	79	**1c**	68	**2c**	75
2,6-(Me)_2_C_6_H_3_	**3d**	88	**1d**	69	**2d**	72
2,4,6-(Me)_3_C_6_H_2_	**3e**	58	**1e**	82	**2e**	77
3-ClC_6_H_4_	**3f**	76	**1f**	97	**2f**	97
4-ClC_6_H_4_	**3g**	78	**1g**	87	**2g**	91
3,5-Cl_2_C_6_H_3_	**3h**	53	**1h**	86	**2h**	59
2-Me-3-ClC_6_H_3_	**3i**	73	**1i**	75	**2i**	67
3-MeOC_6_H_4_	**3j**	74	**1j**	80	**2j**	84
4-MeOC_6_H_4_	**3k**	61	**1k**	86	**2k**	82
3-CF_3_C_6_H_4_	**3l**	89	**1l**	96	**2l**	61

**Table 3:** Degree of fungal growth inhibition for compounds **1a**–**l** and **2a**–**l** at a concentration of 100 μg/mL (bold font indicates medium degrees of inhibition) *.

Comp.	Degree of Inhibition of Mycelial Growth (I ± SD%)								
	A.b.	A.s.	B.c.	C.c.	F.s.	P.i.	P.l.	R.s.	S.s.
**1a**	27.20 ± 0.50	19.19 ± 1.44	16.99 ± 1.38	11.61 ± 0.80	5.56 ± 0.63	7.44 ± 1.58	39.86 ± 0.20	20.62 ± 0.78	13.15 ± 1.32
**1b**	13.01 ± 0.21	8.41 ± 0.41	41.51 ± 0.63	15.97 ± 0.49	21.50 ± 0.25	31.23 ± 0.26	25.86 ± 0.62	38.19 ± 0.78	54.22 ± 0.59
**1c**	20.16 ± 0.48	14.08 ± 0.60	33.78 ± 1.23	16.56 ± 1.40	2.10 ± 0.23	10.13 ± 0.94	12.85 ± 1.00	32.47 ± 2.62	15.13 ± 0.34
**1d**	12.05 ± 0.08	10.60 ± 0.70	33.03 ± 1.92	14.71 ± 0.41	3.22 ± 0.48	8.06 ± 0.50	3.83 ± 0.31	30.88 ± 0.63	15.42 ± 0.79
**1e**	13.40 ± 0.19	25.14 ± 0.79	19.97 ± 0.61	46.65 ± 0.77	14.92 ± 1.77	12.97 ± 0.32	22.24 ± 1.27	30.78 ± 0.16	6.31 ± 0.87
**1f**	22.69 ± 0.26	5.44 ± 0.47	35.03 ± 1.09	6.14 ± 0.46	7.52 ± 0.62	20.61 ± 1.31	26.94 ± 0.30	43.67 ± 0.28	34.46 ± 0.19
**1g**	28.89 ± 0.18	13.54 ± 1.07	50.60 ± 1.08	31.01 ± 0.57	6.17 ± 0.74	14.62 ± 1.30	29.31 ± 1.21	37.63 ± 0.28	22.01 ± 0.30
**1h**	40.54 ± 0.29	26.11 ± 0.76	25.12 ± 0.41	24.19 ± 0.36	30.68 ± 0.24	34.16 ± 0.49	34.77 ± 0.19	48.87 ± 0.21	**60.26 ± 1.07**
**1i**	15.03 ± 0.18	5.26 ± 1.70	27.36 ± 0.85	22.89 ± 0.80	9.43 ± 0.56	13.79 ± 0.67	6.49 ± 0.47	25.92 ± 0.54	23.37 ± 0.19
**1j**	8.50 ± 0.60	23.04 ± 0.33	17.41 ± 0.87	18.37 ± 0.17	19.18 ± 0.91	13.97 ± 0.18	17.60 ± 0.75	39.81 ± 0.38	19.94 ± 0.13
**1k**	24.27 ± 0.11	10.03 ± 1.51	21.90 ± 0.62	14.04 ± 0.55	29.82 ± 1.17	9.14 ± 0.50	24.35 ± 0.55	42.85 ± 0.70	51.02 ± 0.14
**1l**	24.49 ± 0.32	23.11 ± 0.24	4.40 ± 0.60	37.80 ± 0.21	13.39 ± 0.44	26.31 ± 0.28	6.00 ± 0.44	23.65 ± 0.34	49.37 ± 0.11
**2a**	28.27 ± 0.26	37.07 ± 0.23	**69.11 ± 0.36**	33.06 ± 0.97	18.59 ± 0.90	33.44 ± 0.74	10.84 ± 0.24	**68.43 ± 0.82**	**61.60 ± 0.25**
**2b**	36.15 ± 0.55	27.68 ± 0.24	1.24 ± 0.27	21.98 ± 1.47	19.36 ± 0.54	27.92 ± 0.26	31.72 ± 0.21	40.13 ± 0.22	51.99 ± 0.41
**2c**	34.29 ± 0.18	33.22 ± 0.25	55.20 ± 0.49	33.16 ± 0.95	17.54 ± 0.21	34.15 ± 0.32	19.70 ± 0.75	59.38 ± 0.78	58.04 ± 0.93
**2d**	10.64 ± 0.59	17.95 ± 0.59	35.80 ± 2.58	7.77 ± 1.41	12.82 ± 0.25	13.79 ± 0.80	6.98 ± 0.46	29.66 ± 1.13	23.38 ± 0.64
**2e**	12.78 ± 0.65	26.78 ± 0.62	5.53 ± 0.77	18.07 ± 0.10	17.54 ± 0.47	16.39 ± 0.56	7.86 ± 0.55	14.83 ± 0.56	51.76 ± 1.00
**2f**	32.88 ± 0.41	13.53 ± 0.46	22.70 ± 1.17	9.08 ± 0.27	20.72 ± 0.77	27.14 ± 0.56	18.02 ± 0.81	25.30 ± 1.48	47.66 ± 0.88
**2g**	27.36 ± 0.89	38.41 ± 1.13	**65.62 ± 0.55**	29.24 ± 0.60	20.15 ± 1.19	35.64 ± 0.57	14.85 ± 0.92	**67.44 ± 1.46**	**60.01 ± 0.30**
**2h**	45.61 ± 0.32	41.66 ± 0.84	39.57 ± 1.09	38.17 ± 0.45	31.02 ± 0.50	33.38 ± 1.52	38.67 ± 0.35	59.19 ± 0.05	**62.89 ± 0.30**
**2i**	22.89 ± 0.63	34.76 ± 0.47	3.22 ± 0.43	14.22 ± 0.32	17.82 ± 0.30	28.51 ± 0.54	22.62 ± 0.53	28.56 ± 0.77	33.49 ± 0.36
**2j**	34.12 ± 0.16	24.18 ± 0.67	20.90 ± 0.49	21.82 ± 0.25	21.29 ± 0.13	24.83 ± 0.88	22.57 ± 0.13	22.14 ± 1.45	56.33 ± 0.32
**2k**	13.85 ± 0.39	38.85 ± 0.71	22.55 ± 0.19	18.87 ± 0.11	24.00 ± 0.16	34.32 ± 0.38	10.79 ± 0.82	39.05 ± 0.49	49.44 ± 0.11
**2l**	33.78 ± 0.27	14.38 ± 0.21	30.40 ± 1.18	34.09 ± 2.16	29.87 ± 1.11	9.92 ± 0.36	17.32 ± 0.17	37.76 ± 0.30	27.26 ± 0.35
**CZ**	16.33 ± 0.66	16.99 ± 0.56	100 ± 0.00	0	100 ± 0.00	0	95.92 ± 0.70	37.08 ± 1.61	98.28 ± 0.65

* CZ—carbendazim, SD—standard deviation, I = 100—active compound, I = 0—not active compound.

**Table 4:** In vivo efficacy of compounds on rape leaves infected by *A. brassicicola*.

Compound	Protective Efficacy, %	Disease Spot Diameter, mm
1d	92.16	1.33 ± 0.48
2d	19.86	13.60 ± 2.04
Tiadinil	91.57	1.43 ± 1.90
Isotianil	40.78	10.05 ± 3.31
Negative control	–	16.97 ± 4.84

**Table 5:** Experimental details of the XRD study.

Parameter	Value
Crystal data	
Chemical formula	C_14_H_14_ClN_3_O_3_S
Mr, g/mol	339.79
Crystal system, space group	Orthorhombic, *Pbca*
Temperature, K	295
*a*, *b*, *c* (Å)	9.5344(12), 13.538(2), 23.985(3)
*V* (Å^3^)	3095.9(8)
*Z*	8
Radiation type	Mo *K*α
µ, mm^−1^	0.40
Crystal size (mm)	0.49 × 0.31 × 0.18
Data collection	
Diffractometer	Xcalibur, Sapphire3
Absorption correction	Multi-scan *CrysAlis PRO* 1.171.41.123a (Rigaku Oxford Diffraction, 2022) Empirical absorption correction using spherical harmonics, implemented in SCALE3 ABSPACK scaling algorithm.
*T*_min_, *T*_max_	0.846, 1.000
No. of measured, independent and observed [*I* > 2σ(*I*)] reflections	11,607, 4160, 1957
*R*_int_	0.066
(sin θ/λ)_max_ (Å^−1^)	0.714
Refinement	
*R*[*F*^2^ > 2σ(*F*^2^)], *wR*(*F*^2^), *S*	0.077, 0.266, 1.05
No. of reflections	4160
No. of parameters	207
H-atom treatment	H atoms treated by a mixture of independent and constrained refinement
Δρ_max_, Δρ_min_ (e.Å^−3^)	0.30–0.35

## Data Availability

Data are contained within the article and Supplementary Materials.

## Supplementary Materials

The following supporting information can be downloaded at: https://www.mdpi.com/article/10.3390/molecules28010419/s1, ^1^H, ^13^C NMR spectra for compounds **1a**–**l** and **2a**–**l,** 2D HMBS and HSQC spectra for compounds **1a**, **2b**, **2e** and **2j** (Figure S1–S57).

## Sample Availability

Samples of the compounds **1a**–**l**, **2a**–**l**, **3a**–**l** are available from the authors.
