# Supplementary material for: Synthesis, Fungicidal Activity and Plant Protective Properties of 1,2,3-Thiadiazole and Isothiazole-Based N-acyl-N-arylalaninates
Source: Molecules. 2023 Jan 3;28(1):419. doi: 10.3390/molecules28010419 (PMC9822468; doi:10.3390/molecules28010419)
Supplement: Supplementary file 1 [file molecules-28-00419-s001.zip › Supporting Information.pdf]

Supporting Information

Synthesis, Fungicidal Activity and Plant Protective Properties of 1,2,3-Thiadiazole and Isothiazole-Based *N*-acyl-*N*-arylalaninates

Tatiana A. Kalinina [1,*], Valeriya I. Balandina [1], Konstantin L. Obydennov [1], Pavel A. Slepukhin [2], Zhijin Fan [3], Vasiliy A. Bakulev [1] and Tatiana V. Glukhareva [1]

[1] Ural Federal University named after the first President of Russia B.N. Yeltsin, 19 Mira Street, 620002 Ekaterinburg, Russia

[2] Postovsky Institute of Organic Synthesis, Ural Branch of Russian Academy of Sciences, 22/20 S. Kovalevskaya Street, 620108 Ekaterinburg, Russia

[3] State Key Laboratory of Elemento-Organic Chemistry, College of Chemistry, Nankai University, No. 94, Weijin Road, 300071 Tianjin, China

***** Correspondence: t.a.kalinina@urfu.ru; v.a.bakulev@urfu.ru; Tel.: +7-3433754818

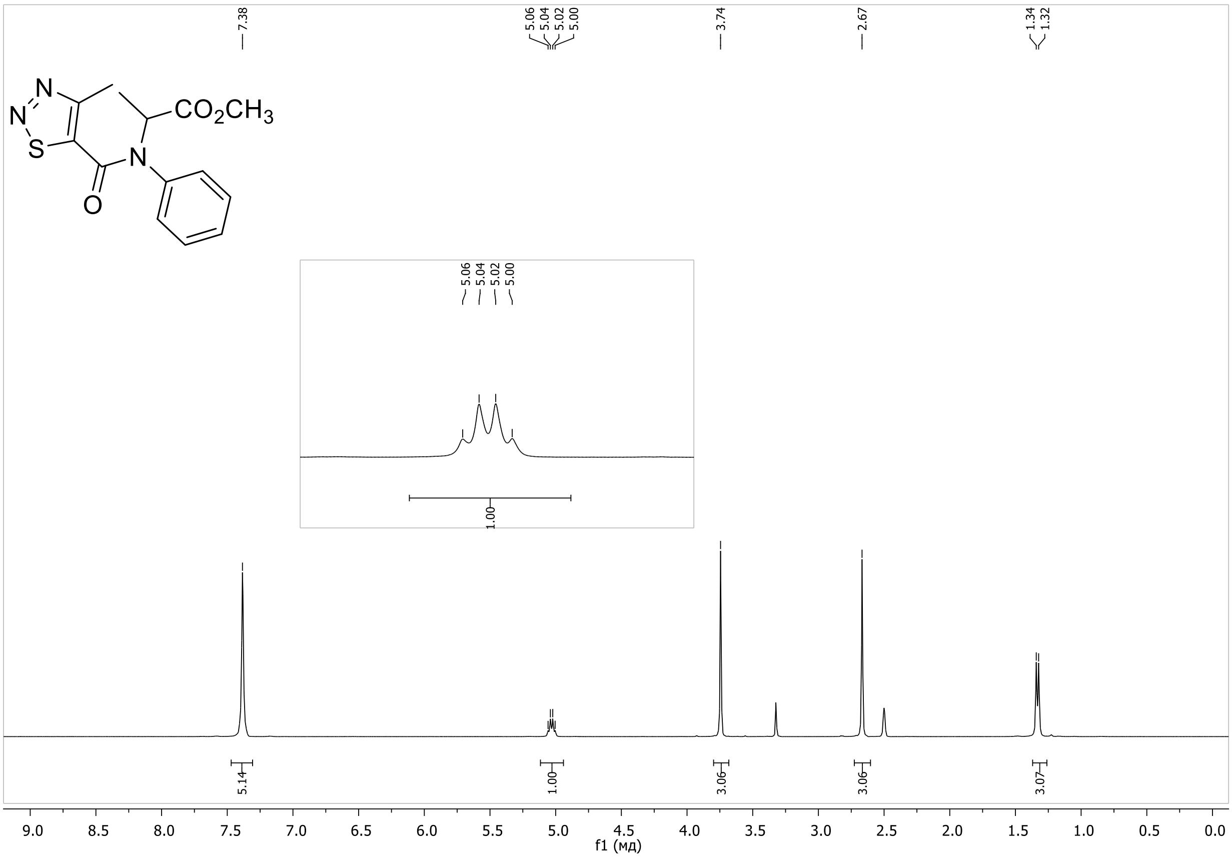

Figure S1. ^{1}H NMR of compound **1a**

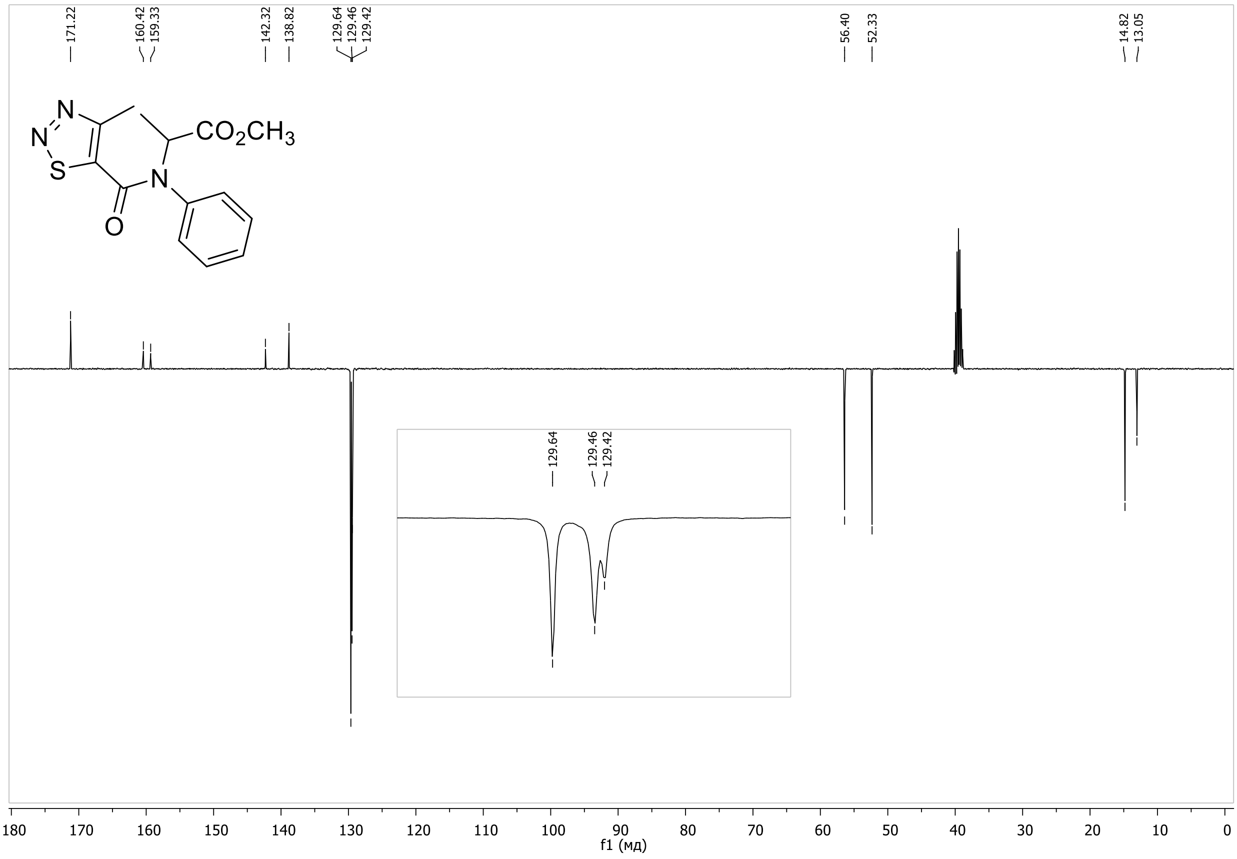

Figure S2. ^{13}C NMR of compound **1a**

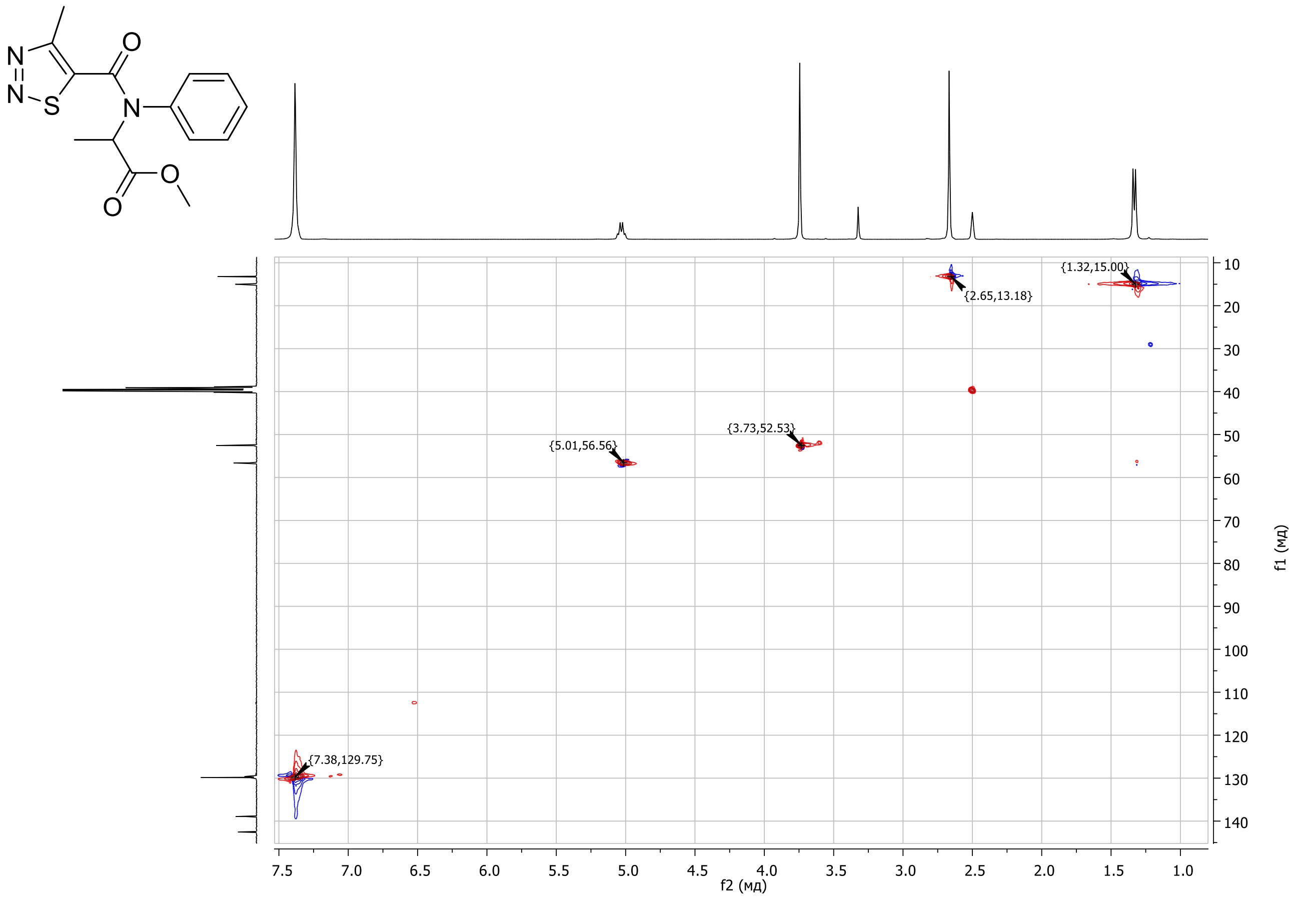

Figure S3. Part of the HSQC ^{1}H–^{13}C NMR spectrum of **1a**

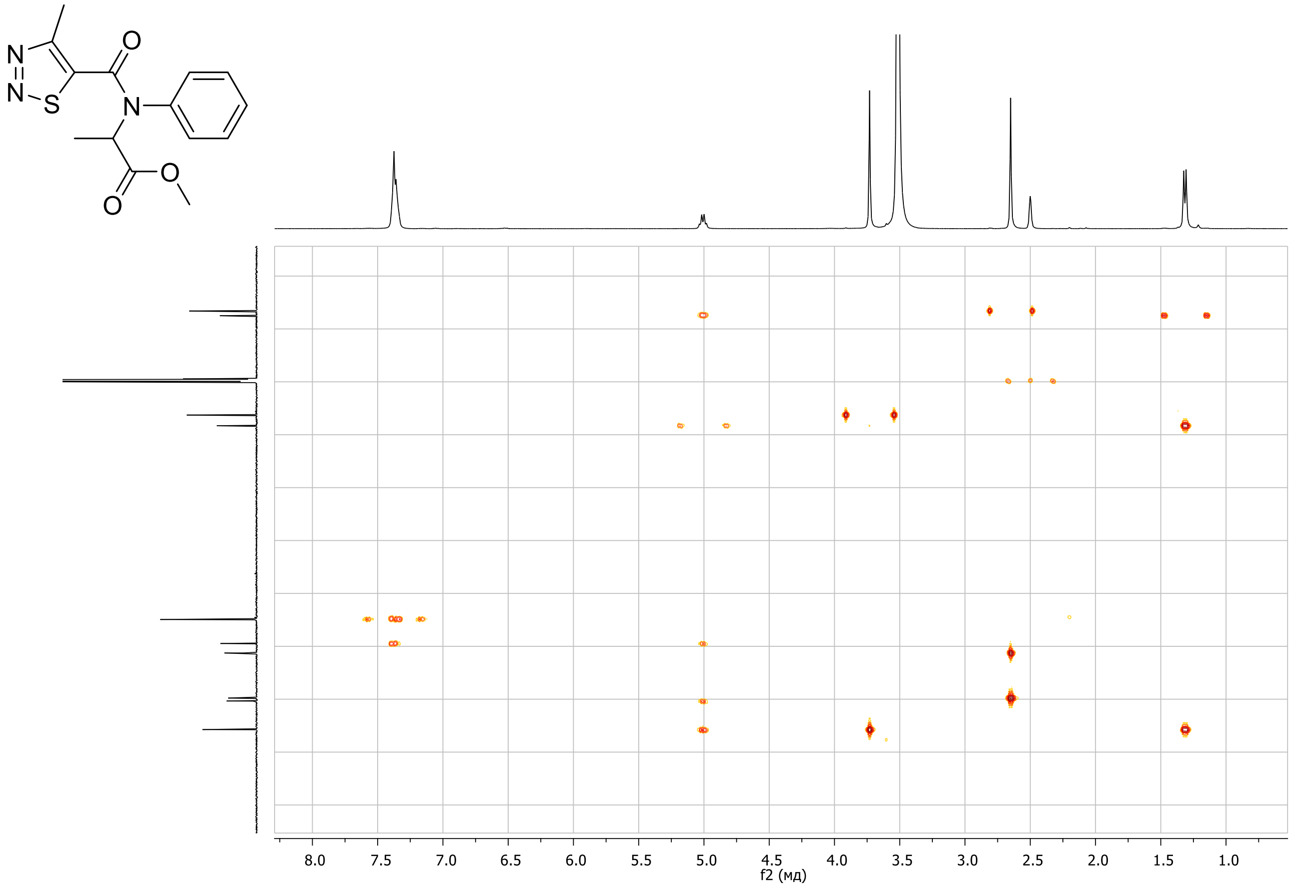

Figure S4. The HMBC ^{1}H–^{13}C NMR spectrum of **1a**

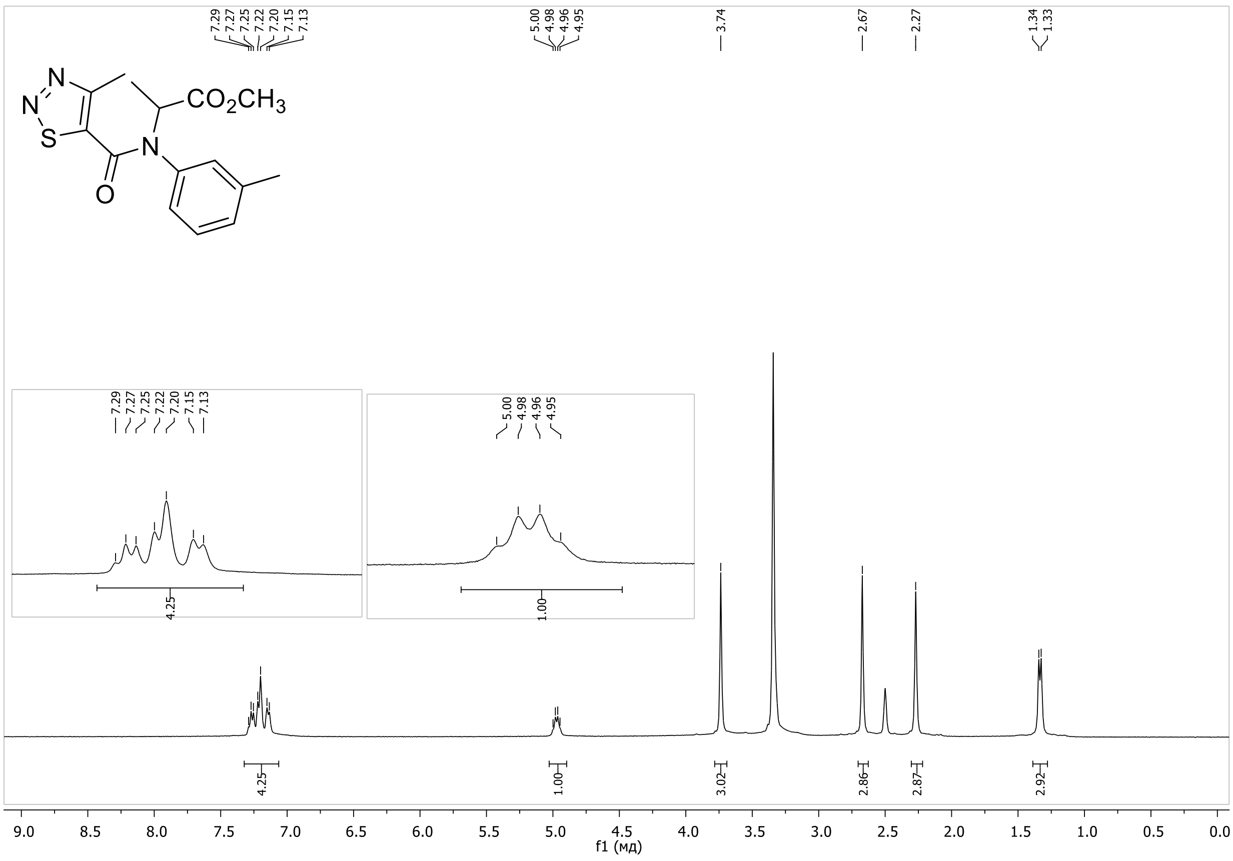

Figure S5. ^{1}H NMR of compound **1b**

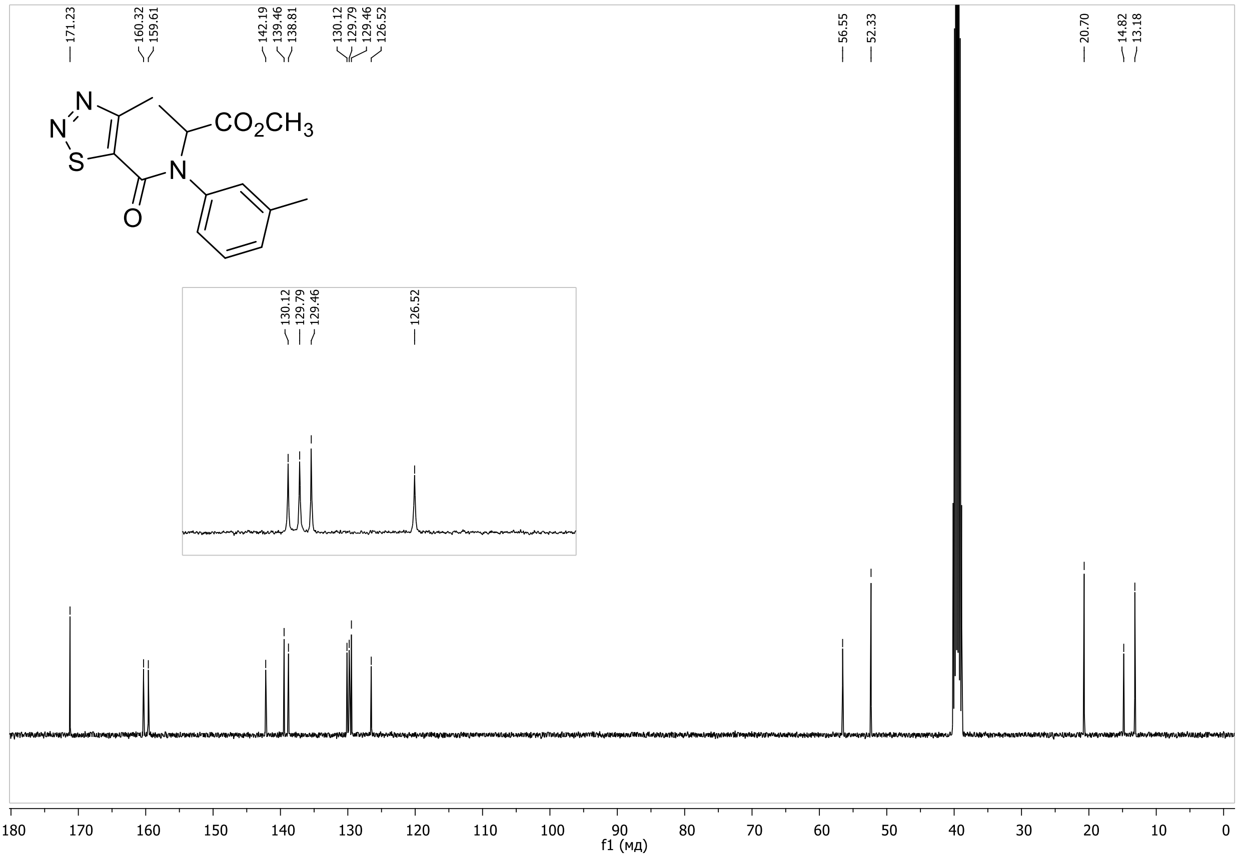

Figure S6. ^{13}C NMR of compound **1b**

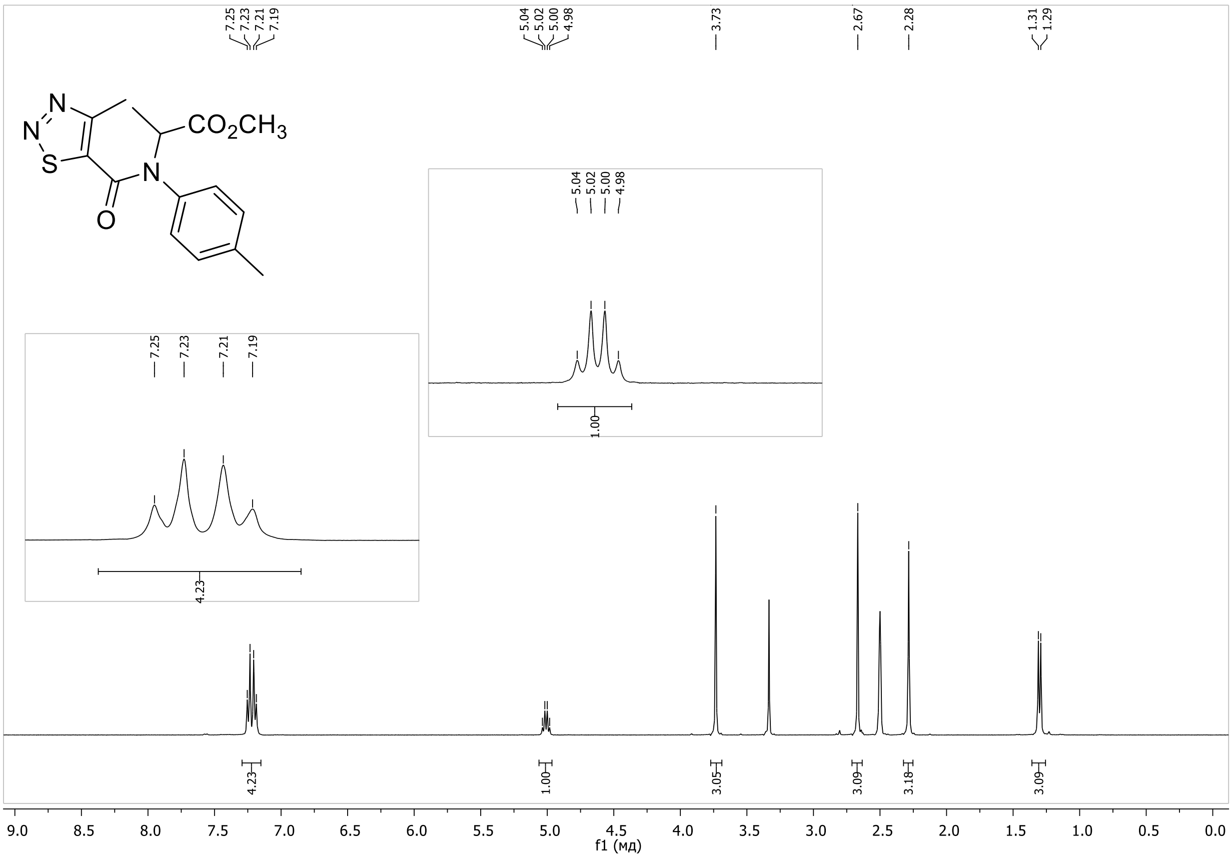

Figure S7. ^{1}H NMR of compound **1c**

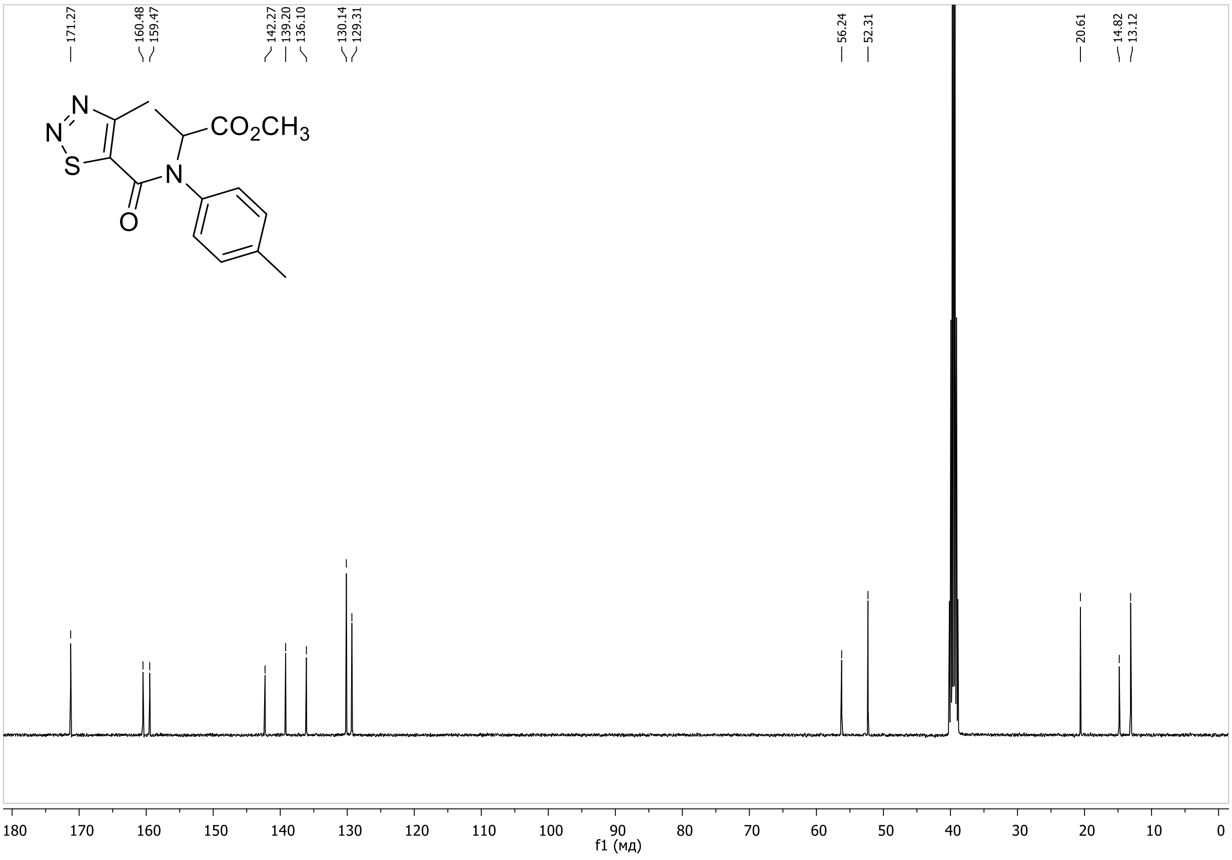

Figure S8. ^{13}C NMR of compound **1c**

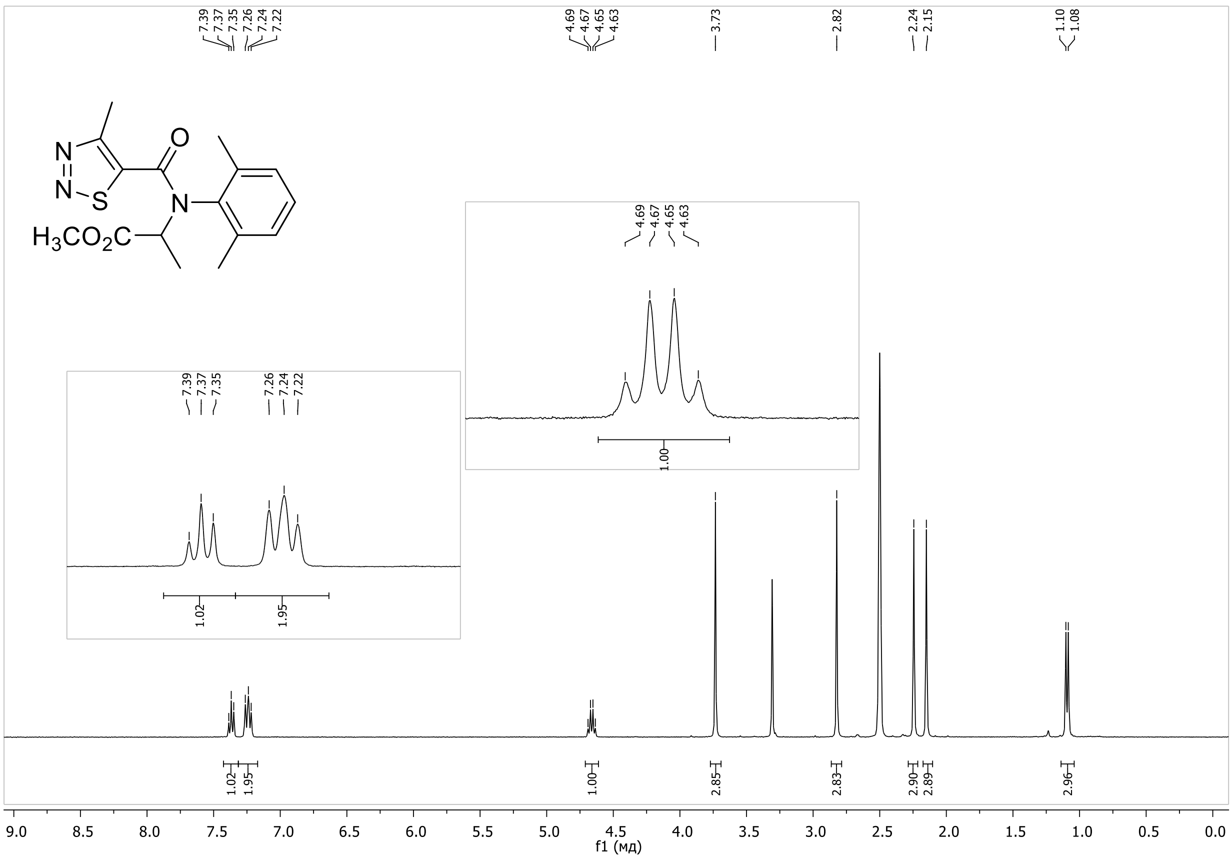

Figure S9. ^{1}H NMR of compound **1d**

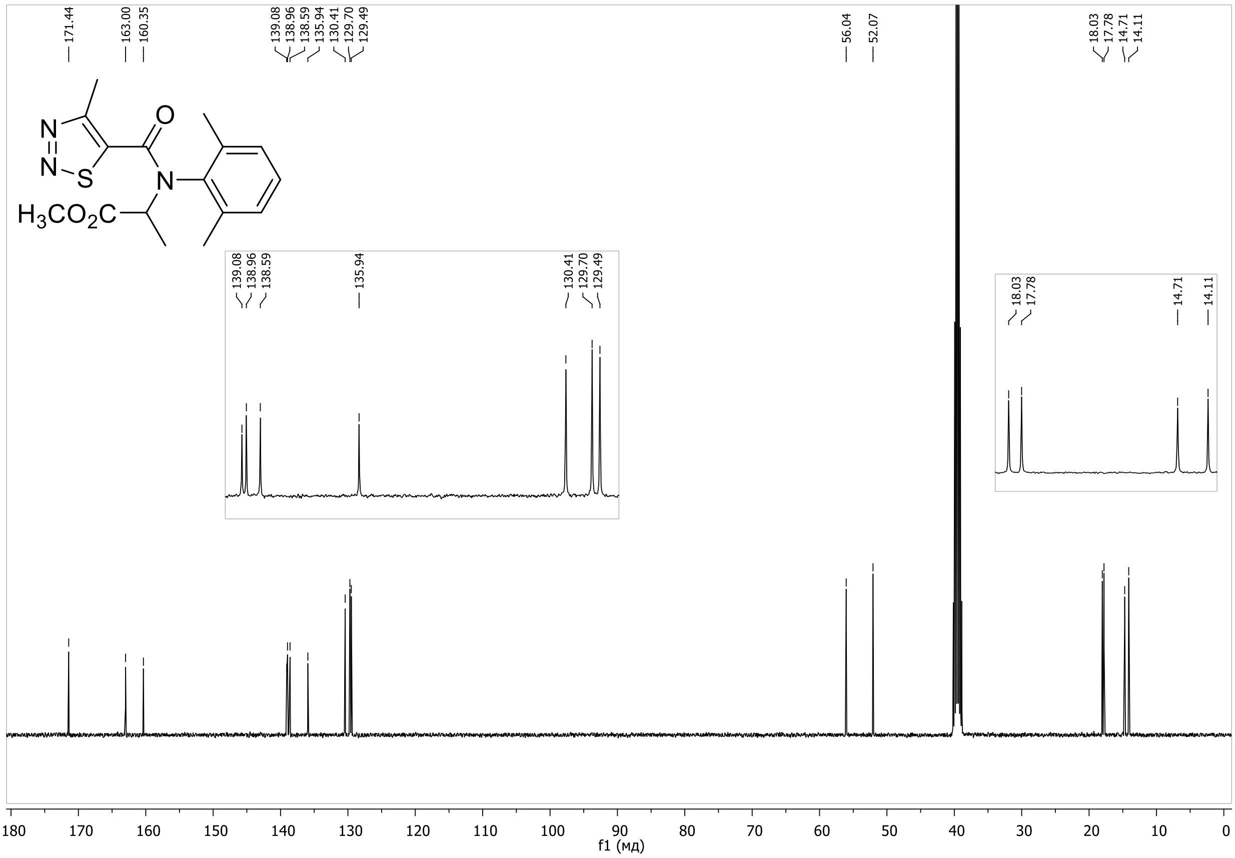

Figure S10. ^{13}C NMR of compound **1d**

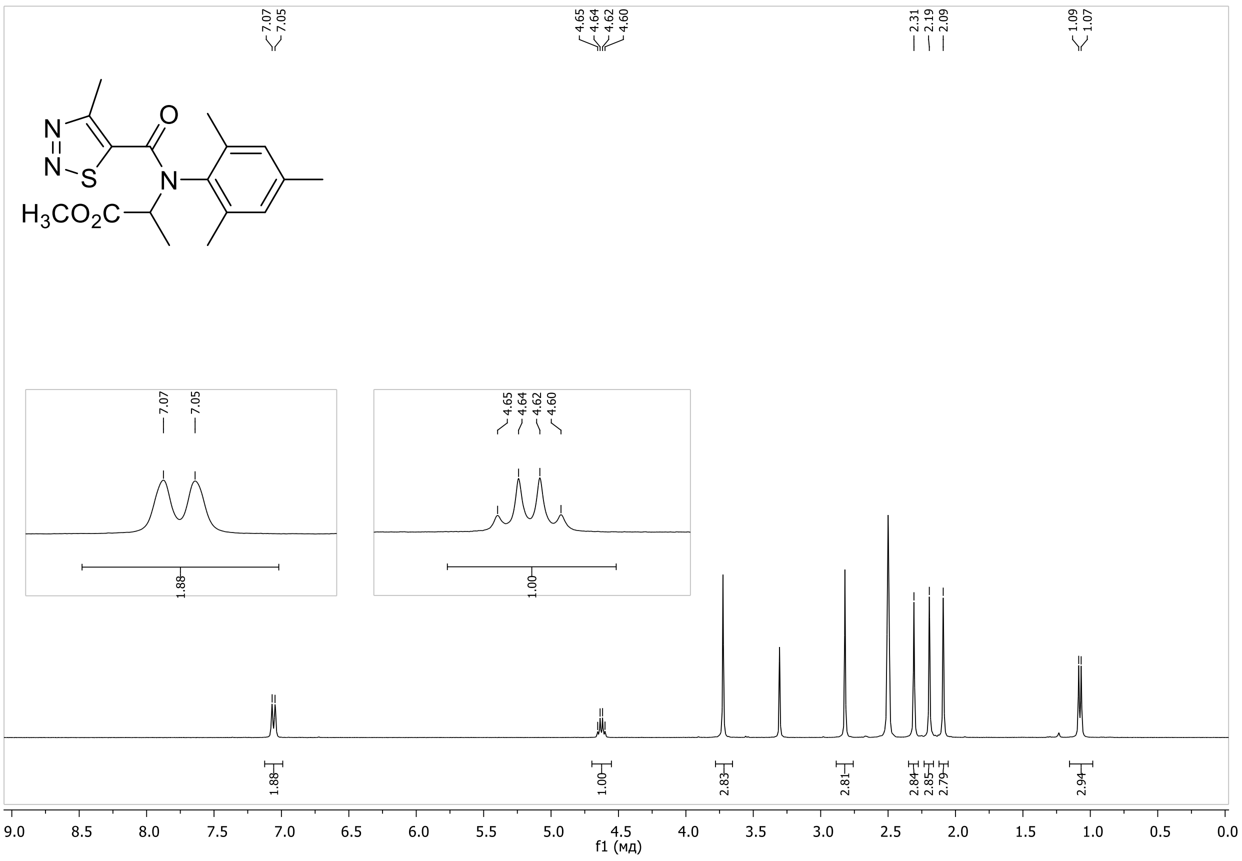

Figure S11. ^{1}H NMR of compound **1e**

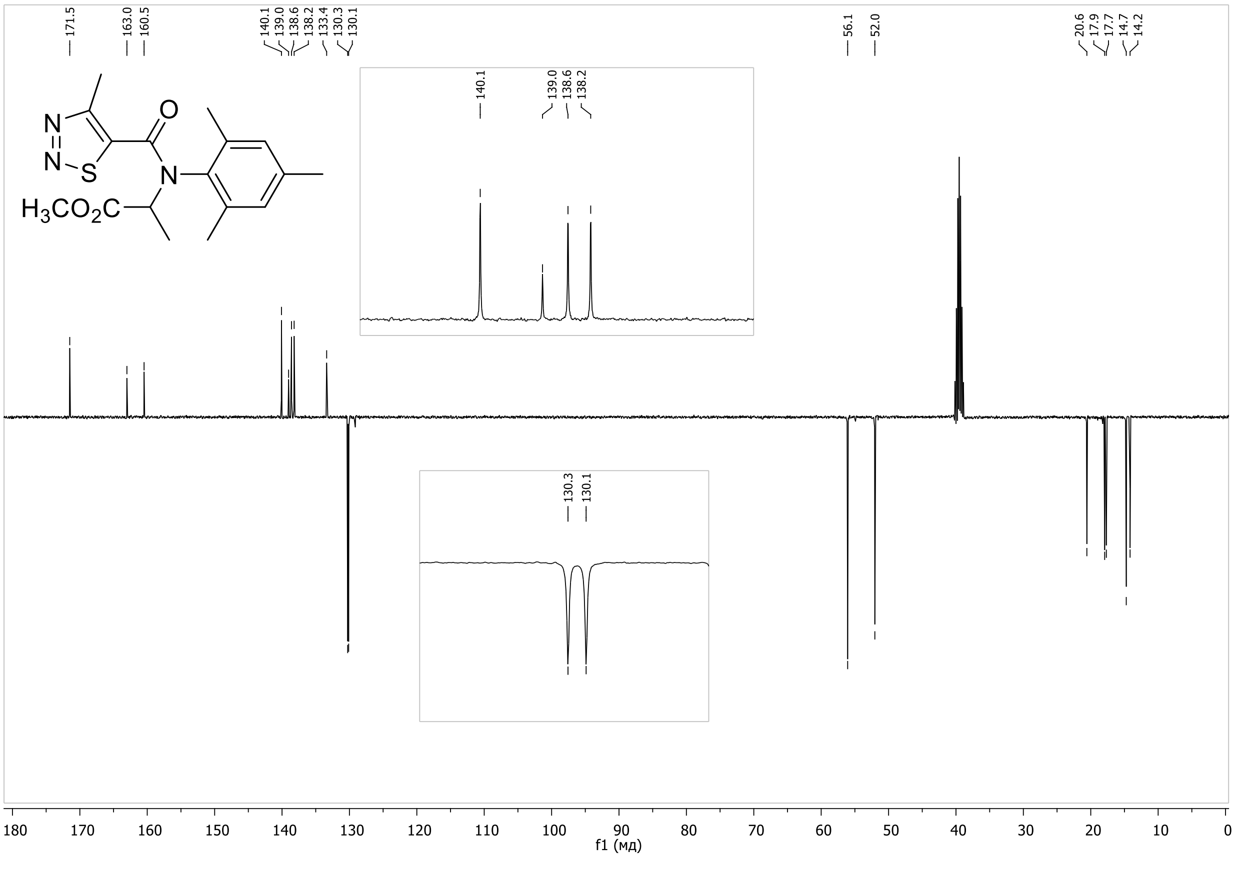

Figure S12. ^{13}C NMR of compound **1e**

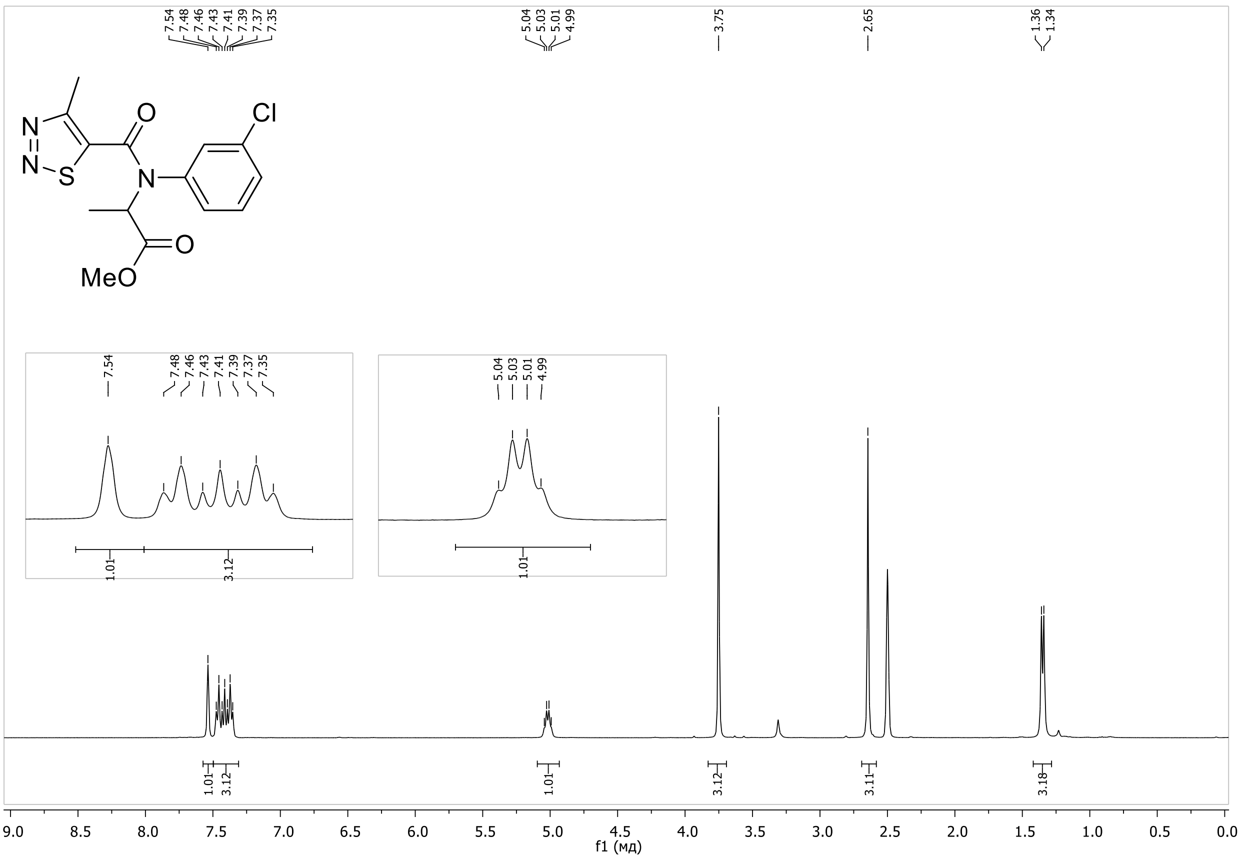

Figure S13. ^{1}H NMR of compound **1f**

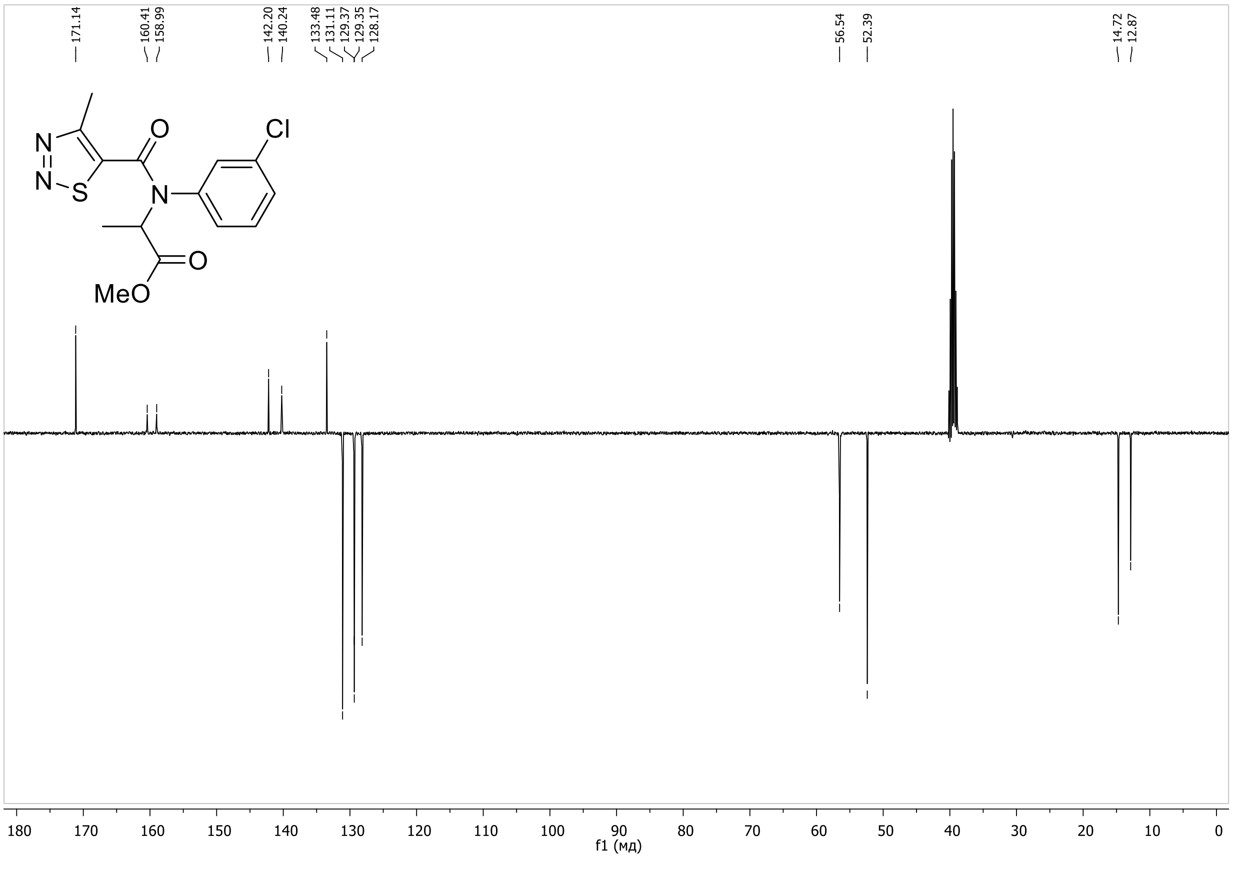

Figure S14. ^{13}C NMR of compound **1f**

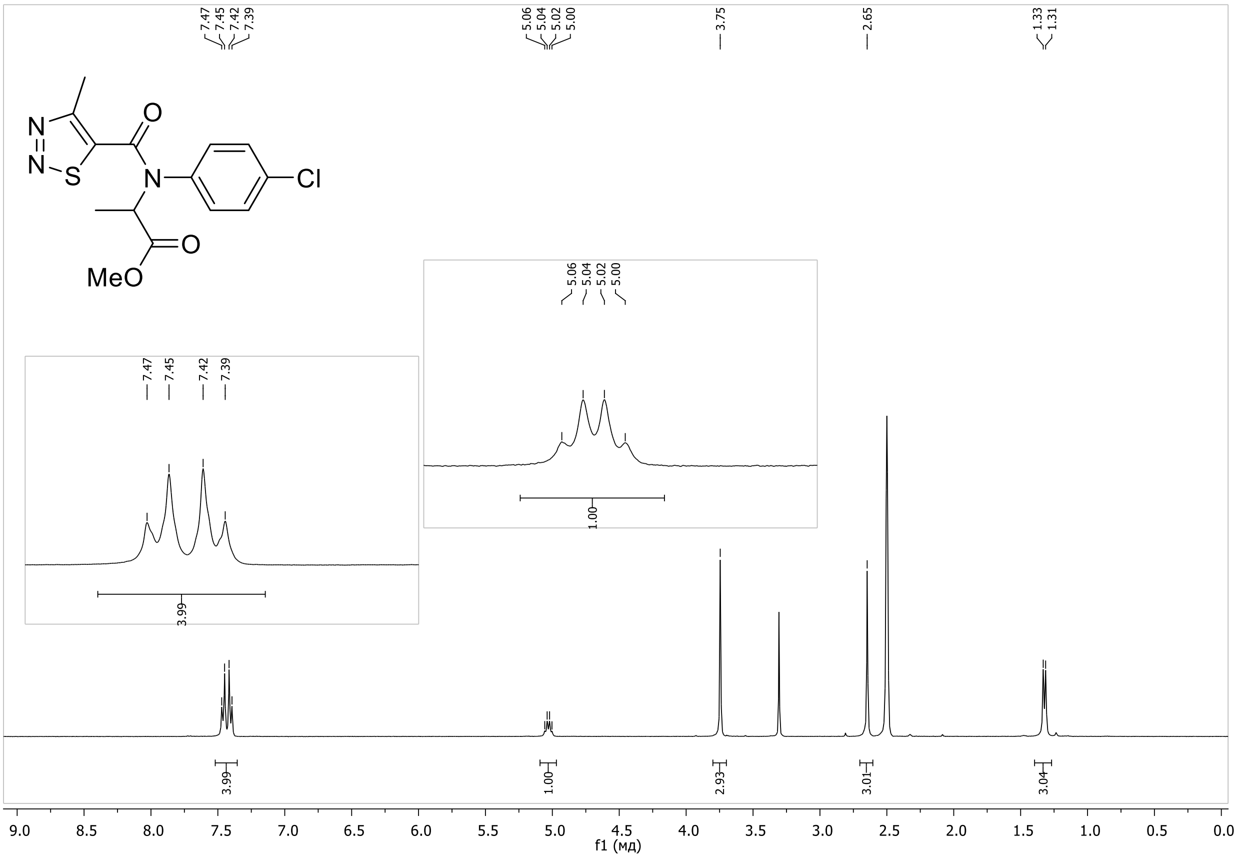

Figure S15. ^{1}H NMR of compound **1g**

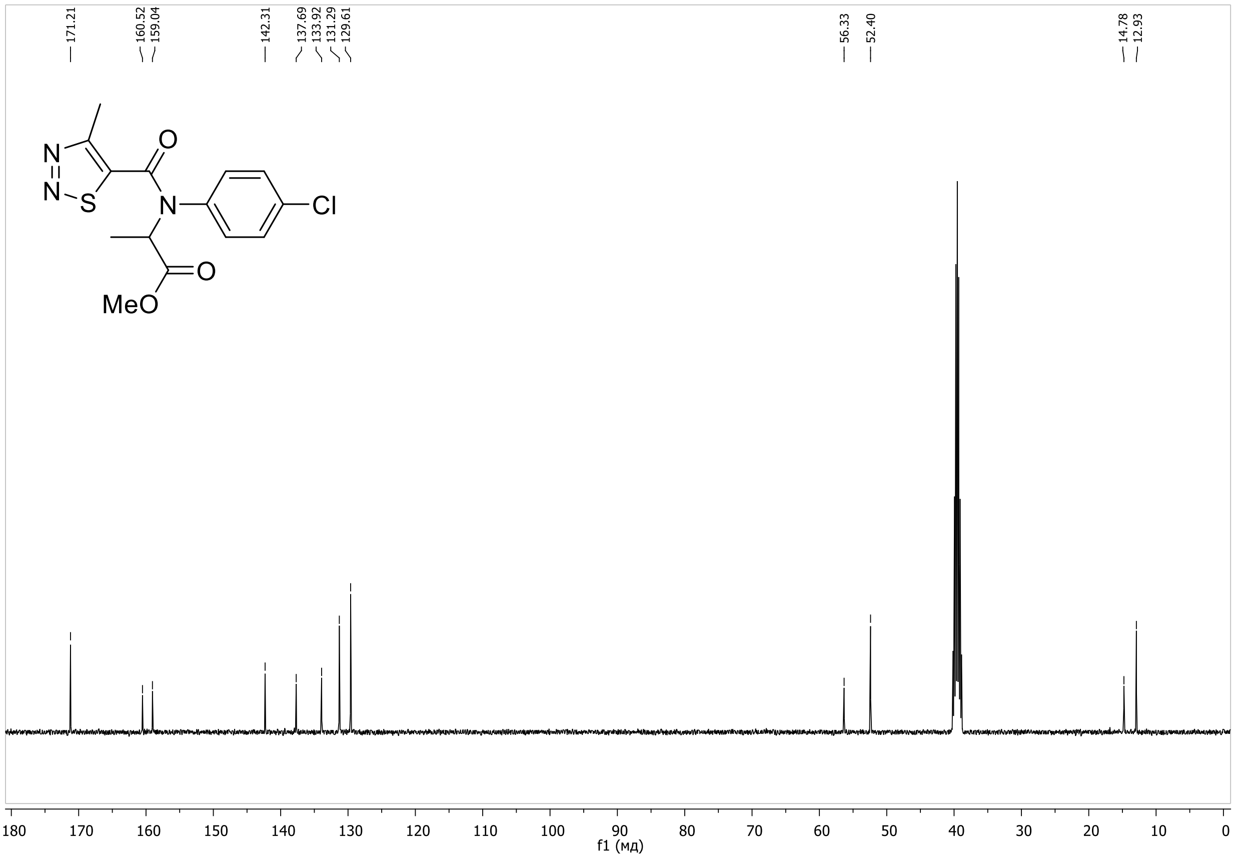

Figure S16. ^{13}C NMR of compound **1f**

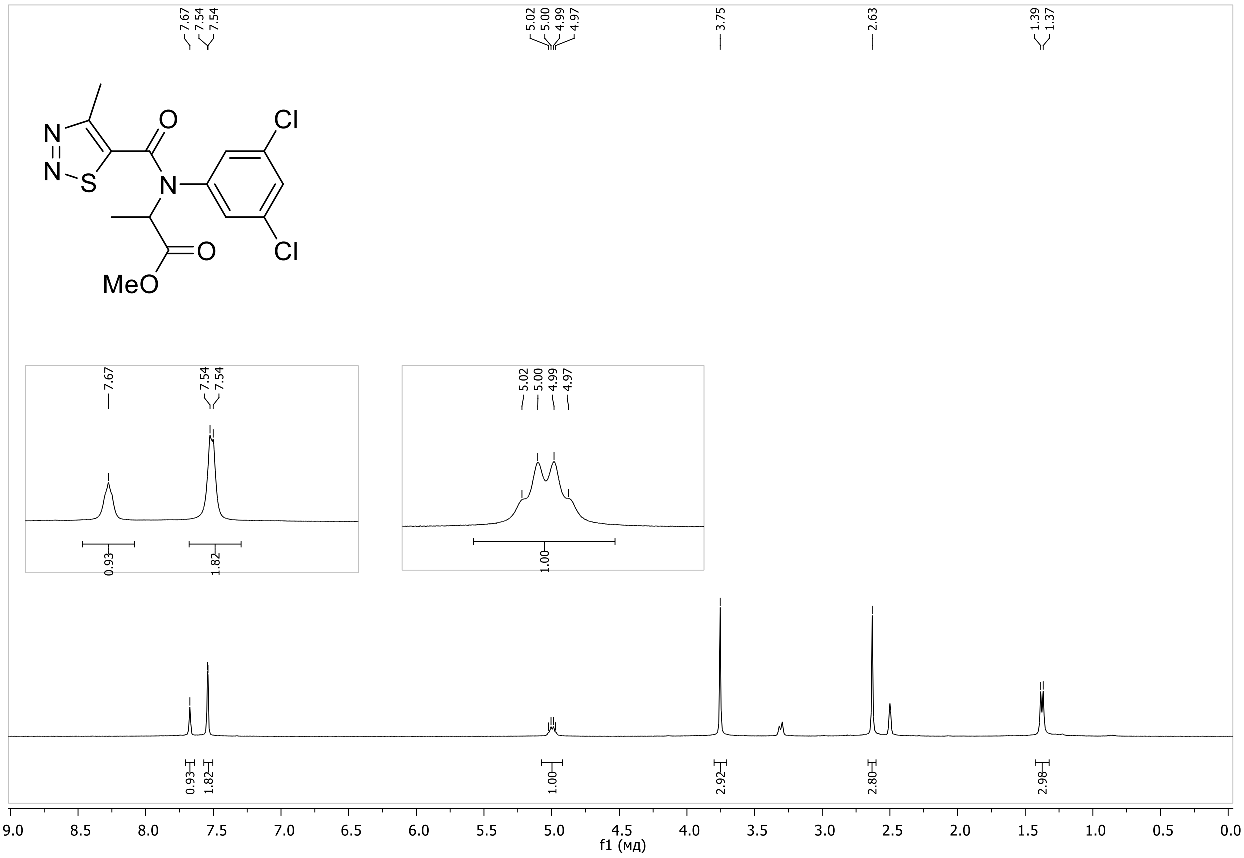

Figure S17. ^{1}H NMR of compound **1h**

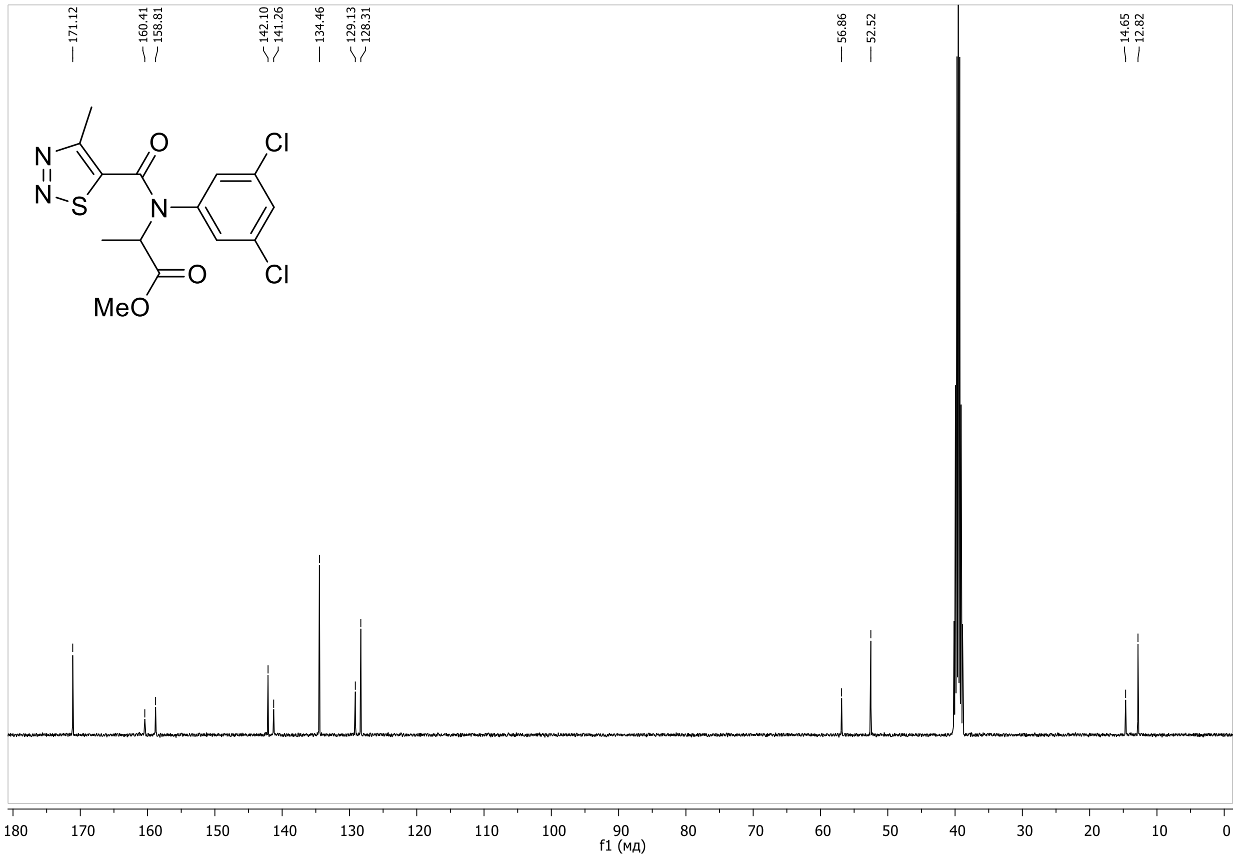

Figure S18. ^{13}C NMR of compound **1h**

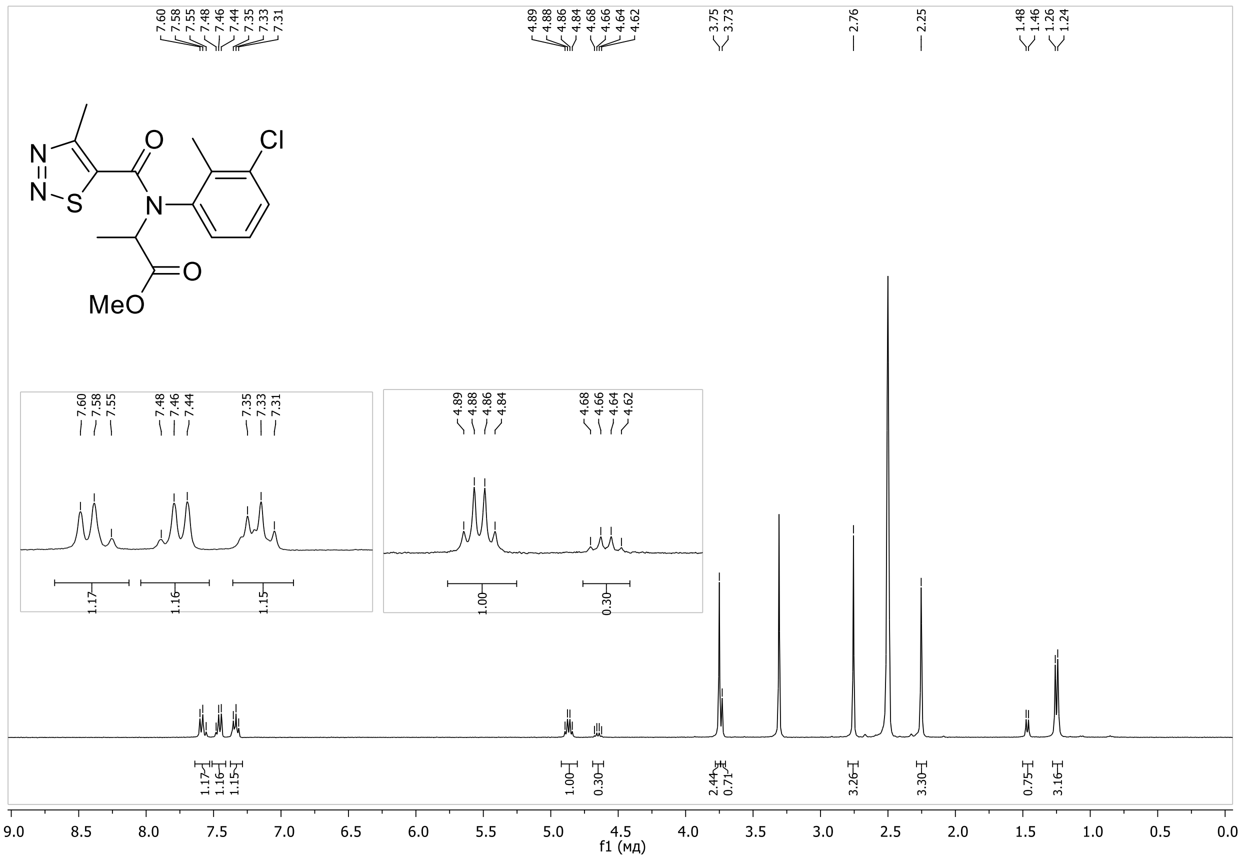

Figure S19. ^{1}H NMR of compound **1i**

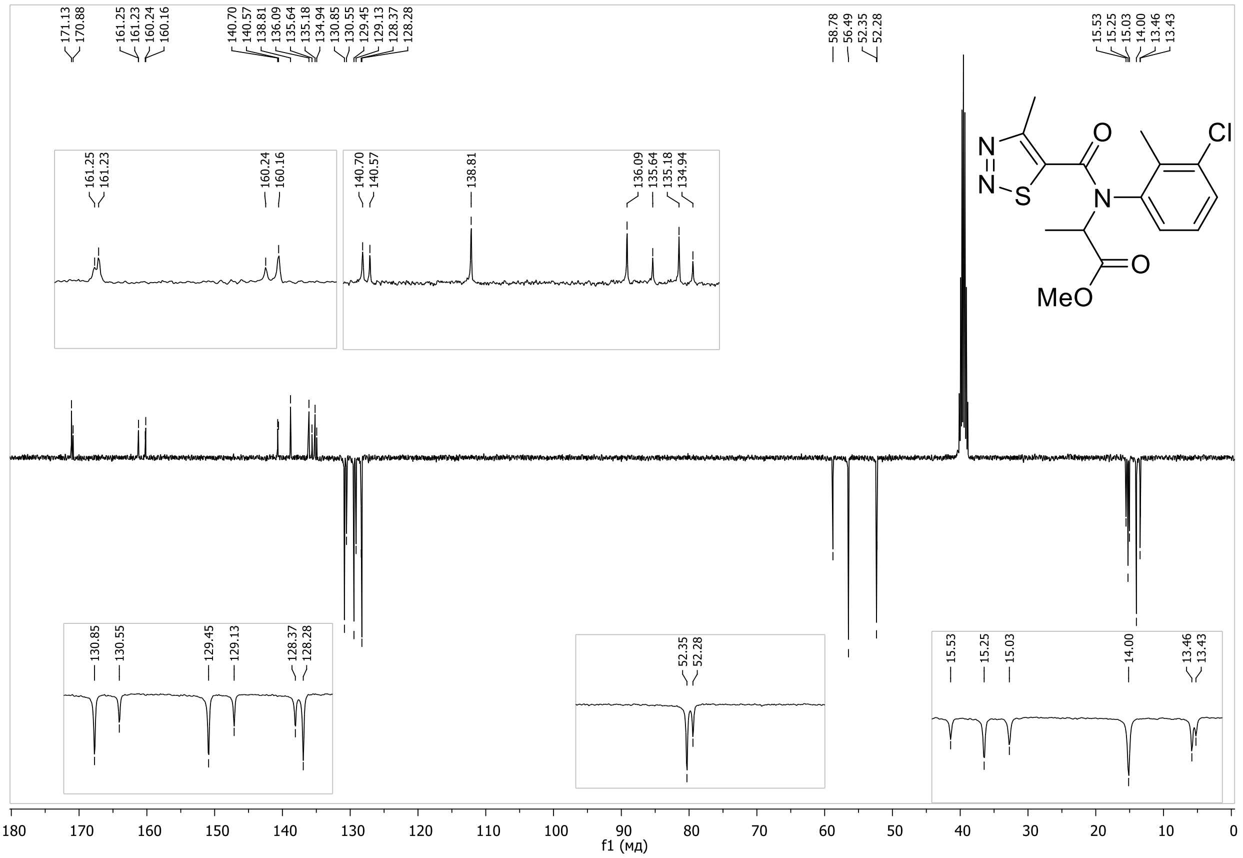

Figure S20. ^{13}C NMR of compound **1i**

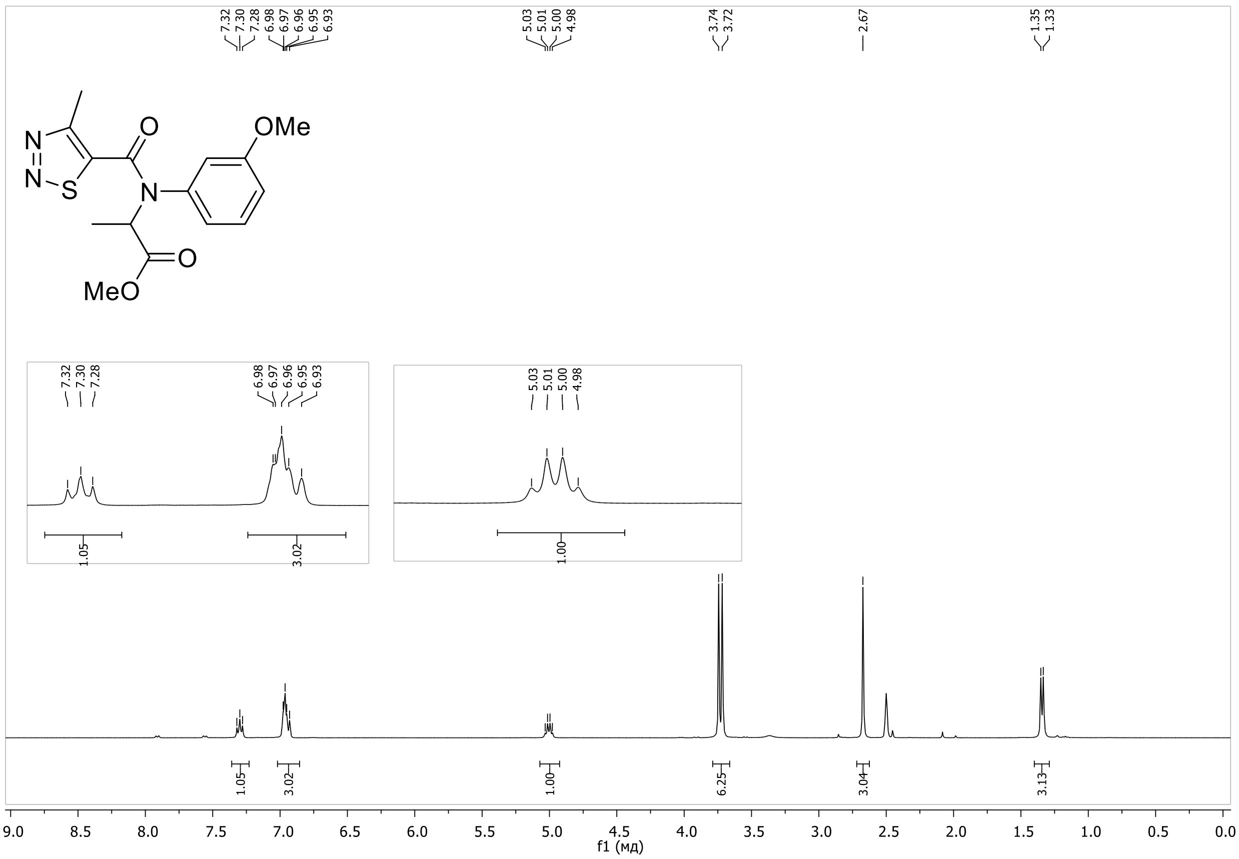

Figure S21. ^{1}H NMR of compound **1j**

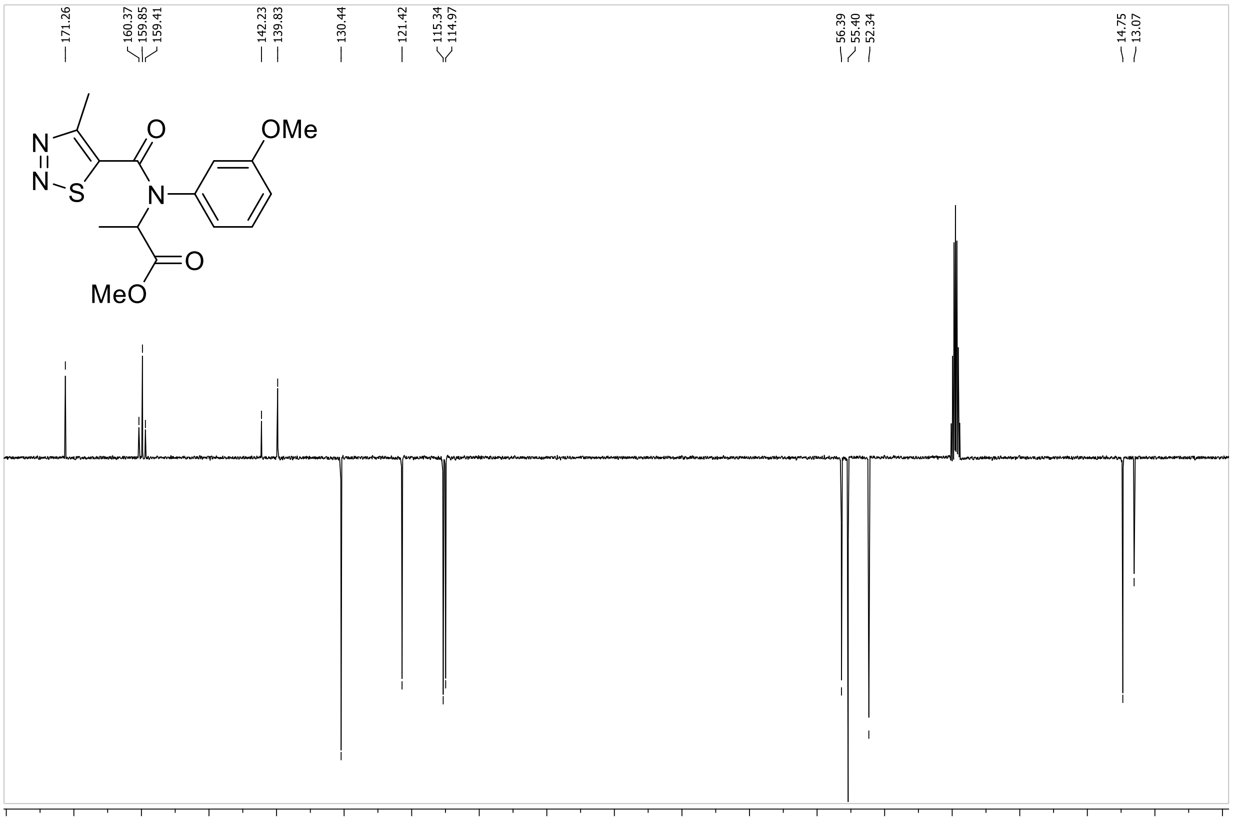

Figure S22. ^{13}C NMR of compound **1j**

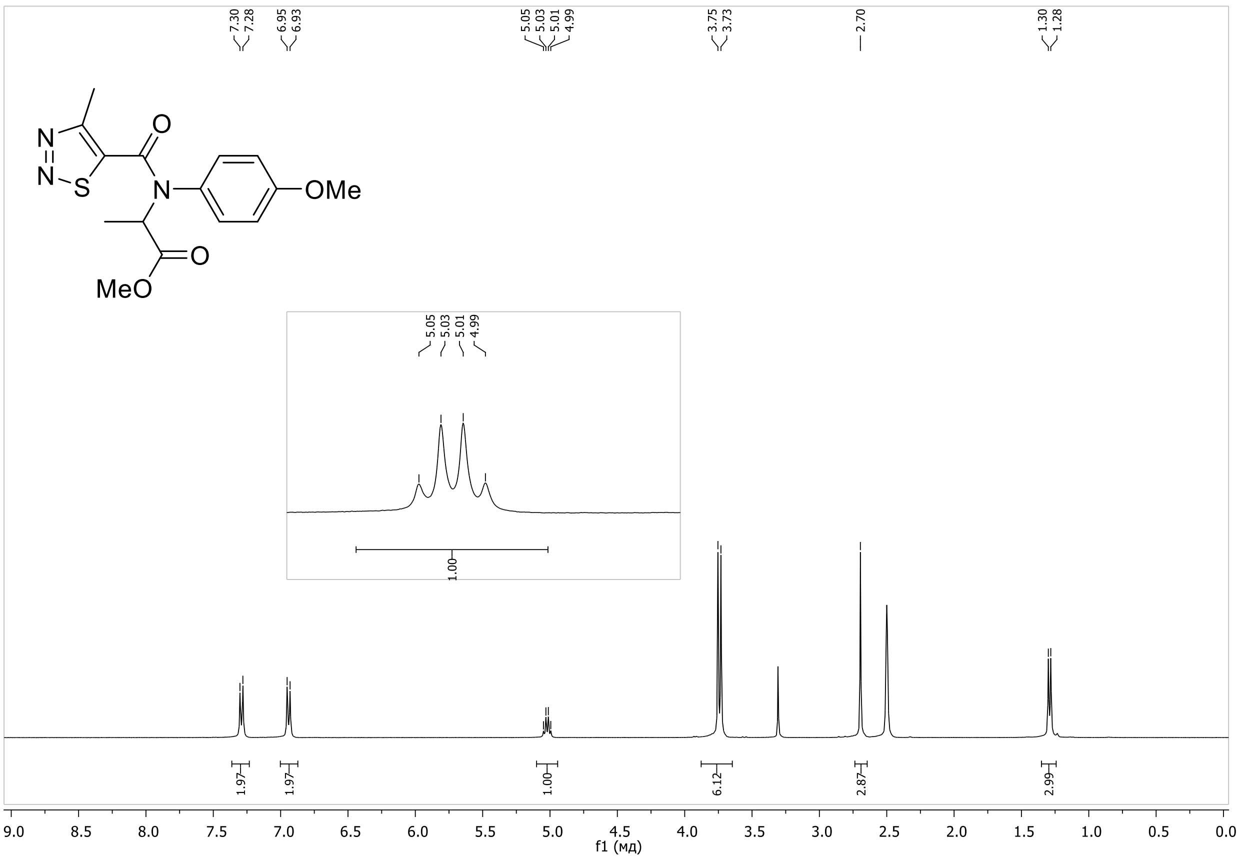

Figure S23. ^{1}H NMR of compound **1k**

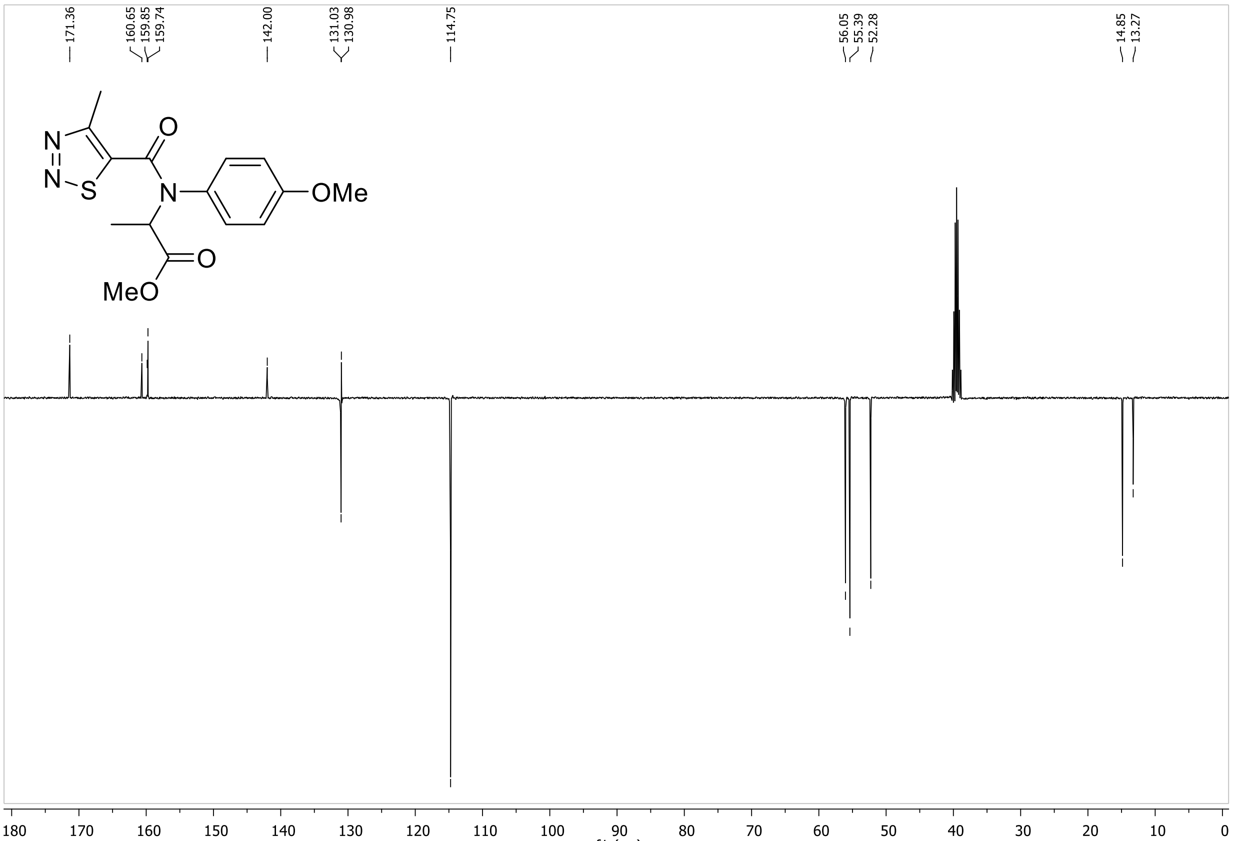

Figure S24. ^{13}C NMR of compound **1k**

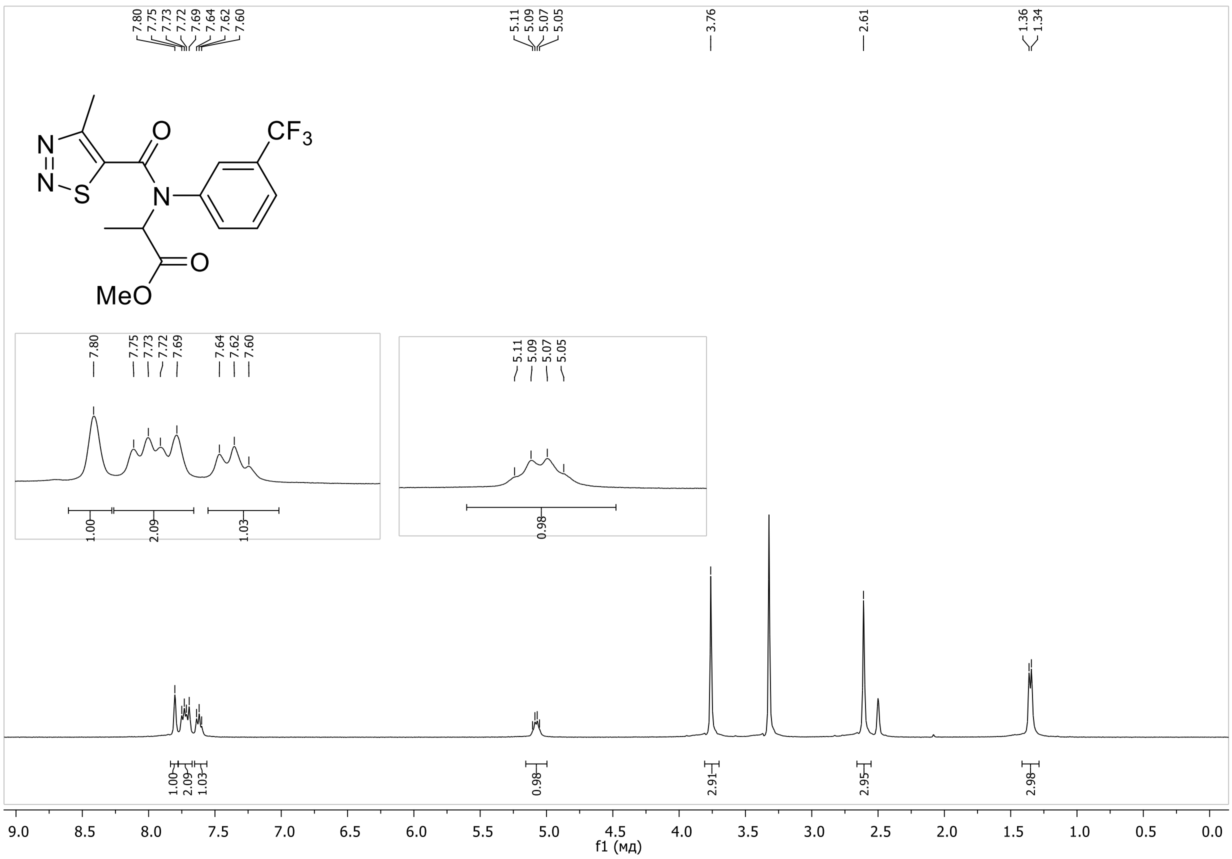

Figure S25. ^{1}H NMR of compound **1l**

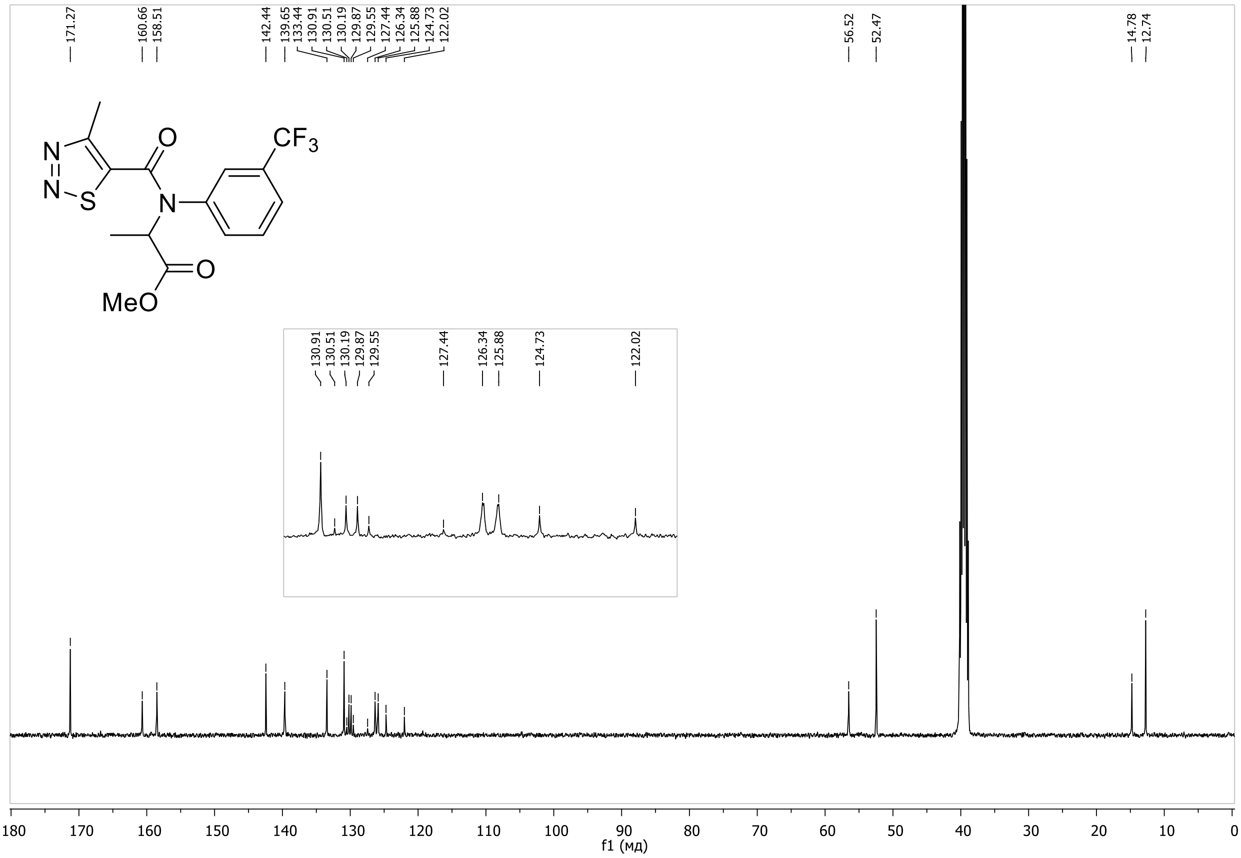

Figure S26. ^{13}C NMR of compound **1l**

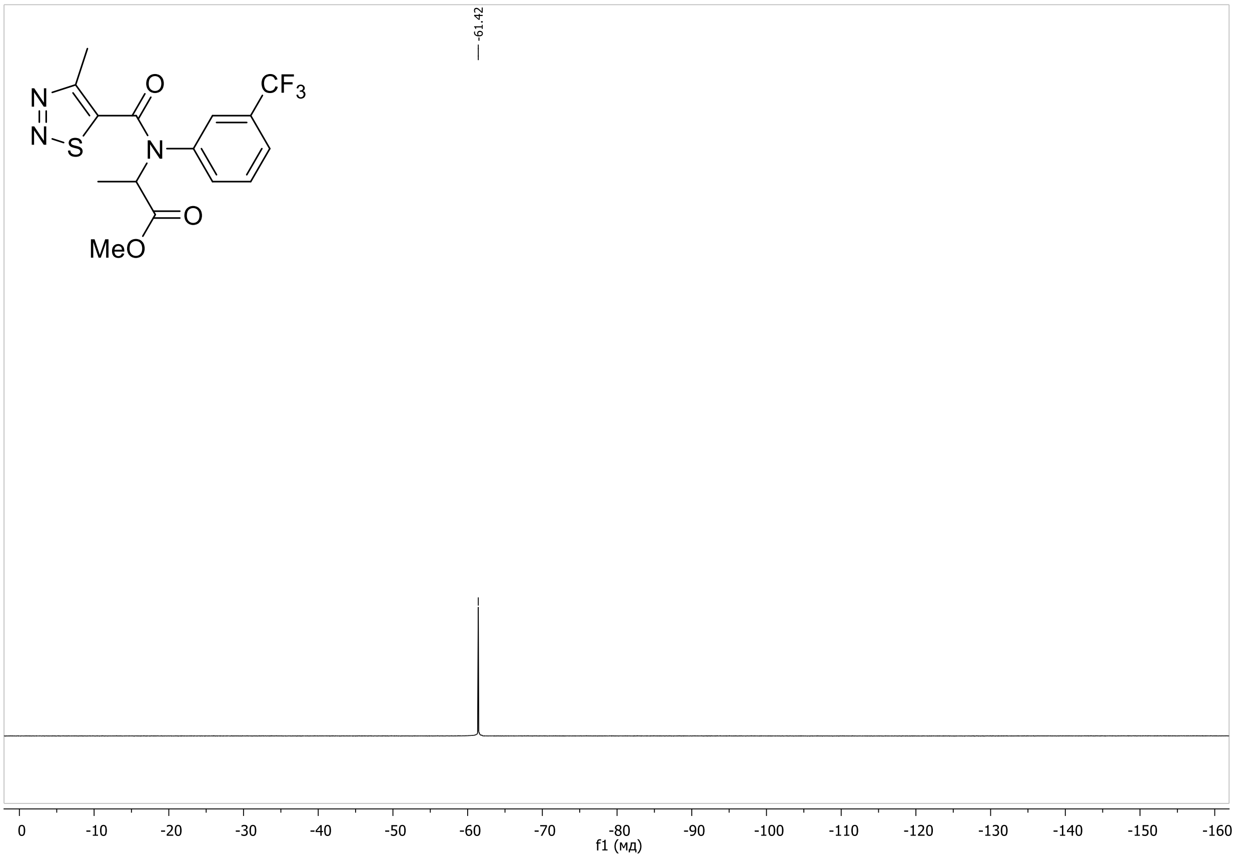

Figure S27. ^{19}F NMR of compound **1l**

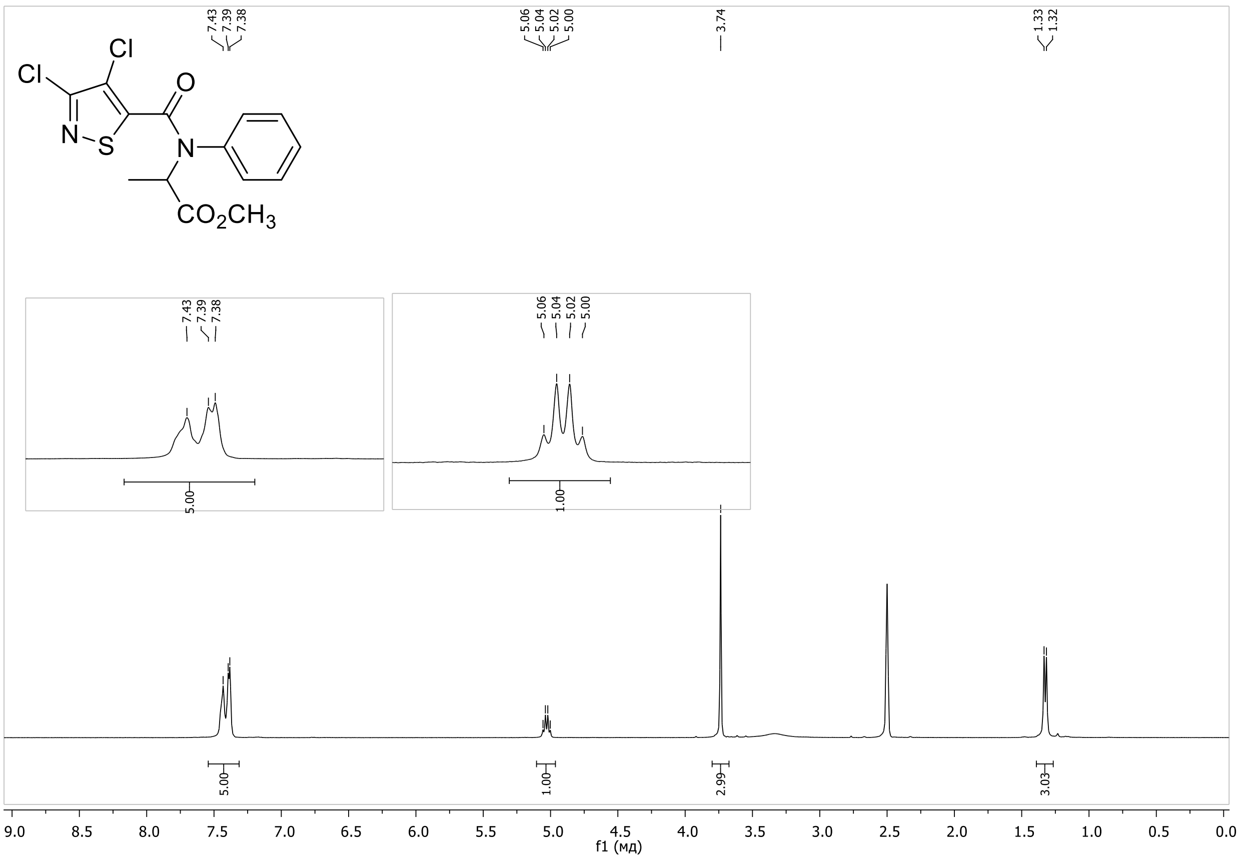

Figure S28. ^{1}H NMR of compound **2a**

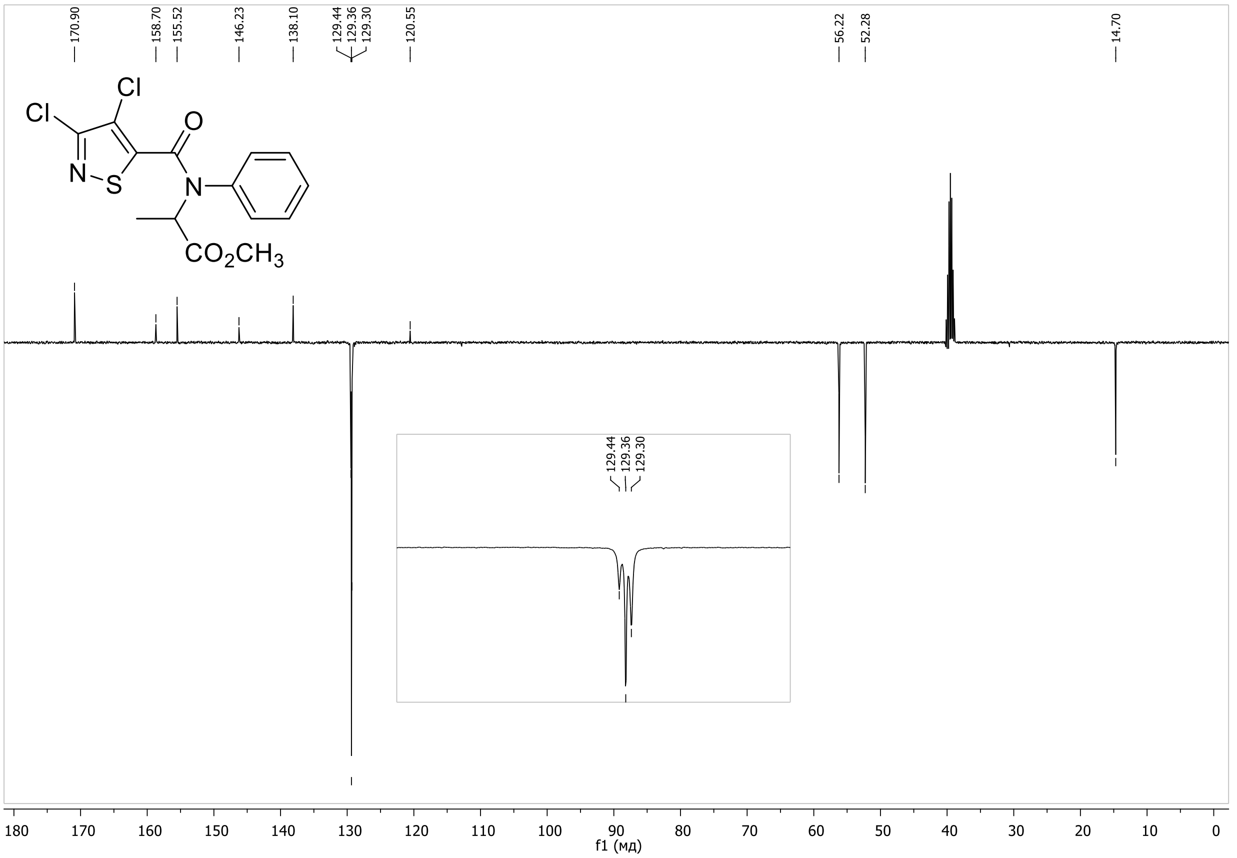

Figure S29. ^{13}C NMR of compound **2a**

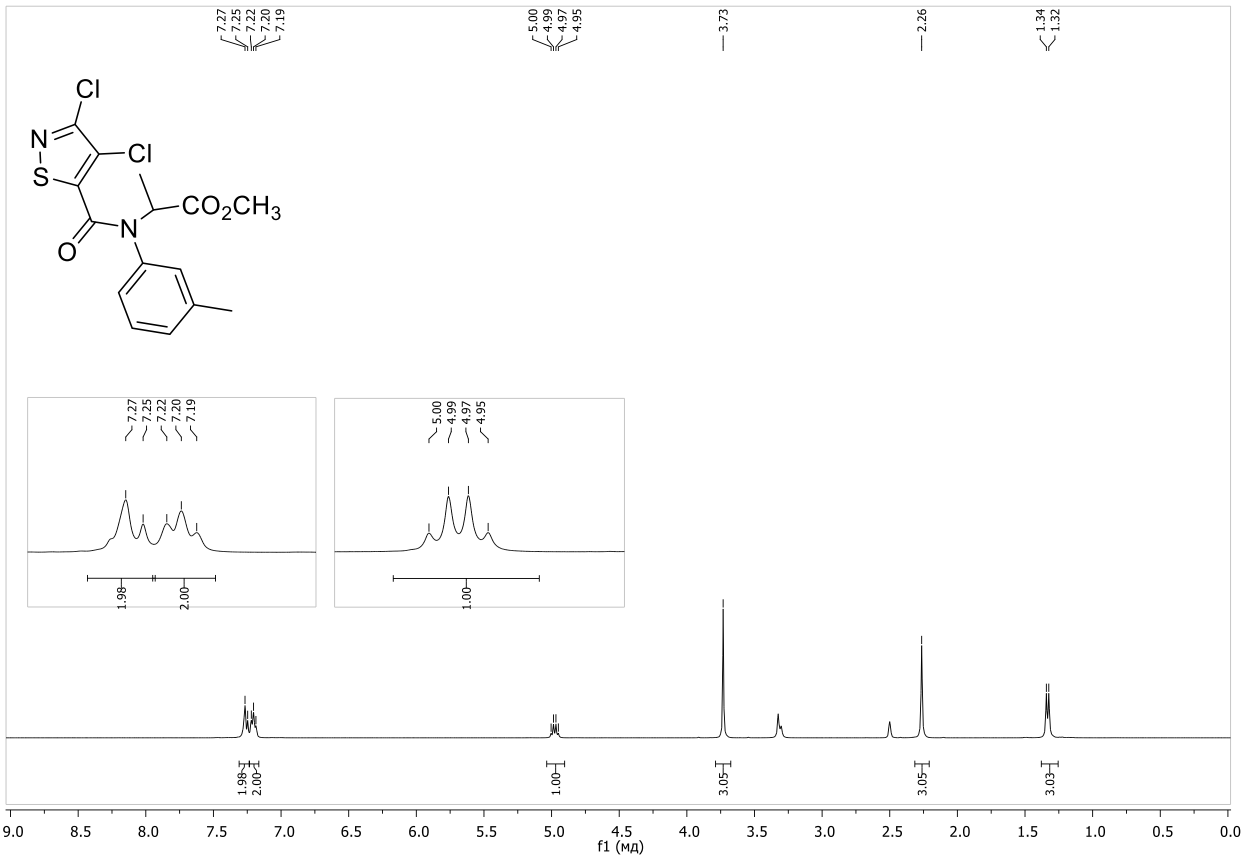

Figure S30. ^{1}H NMR of compound **2b**

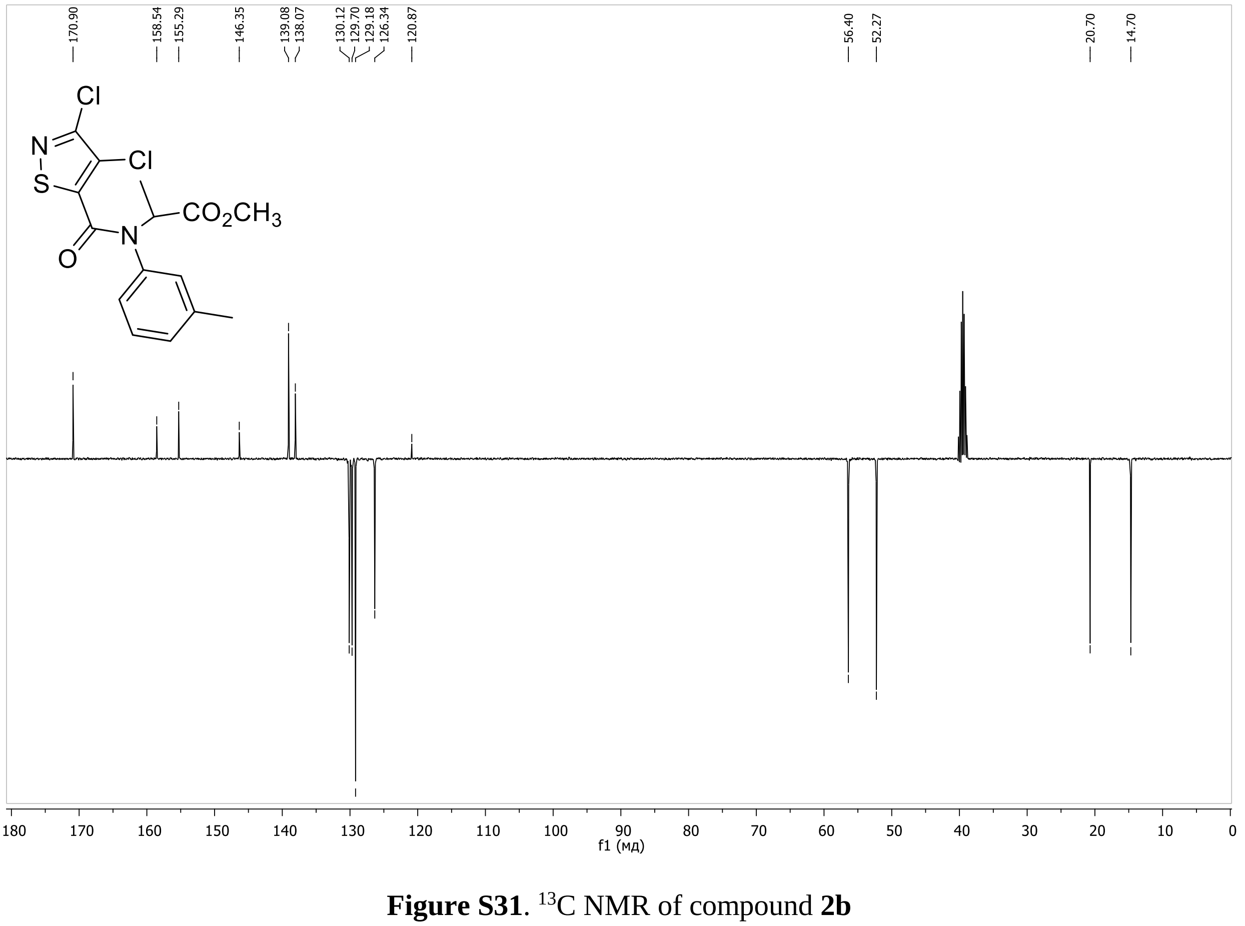

Figure S31. ^{13}C NMR of compound **2b**

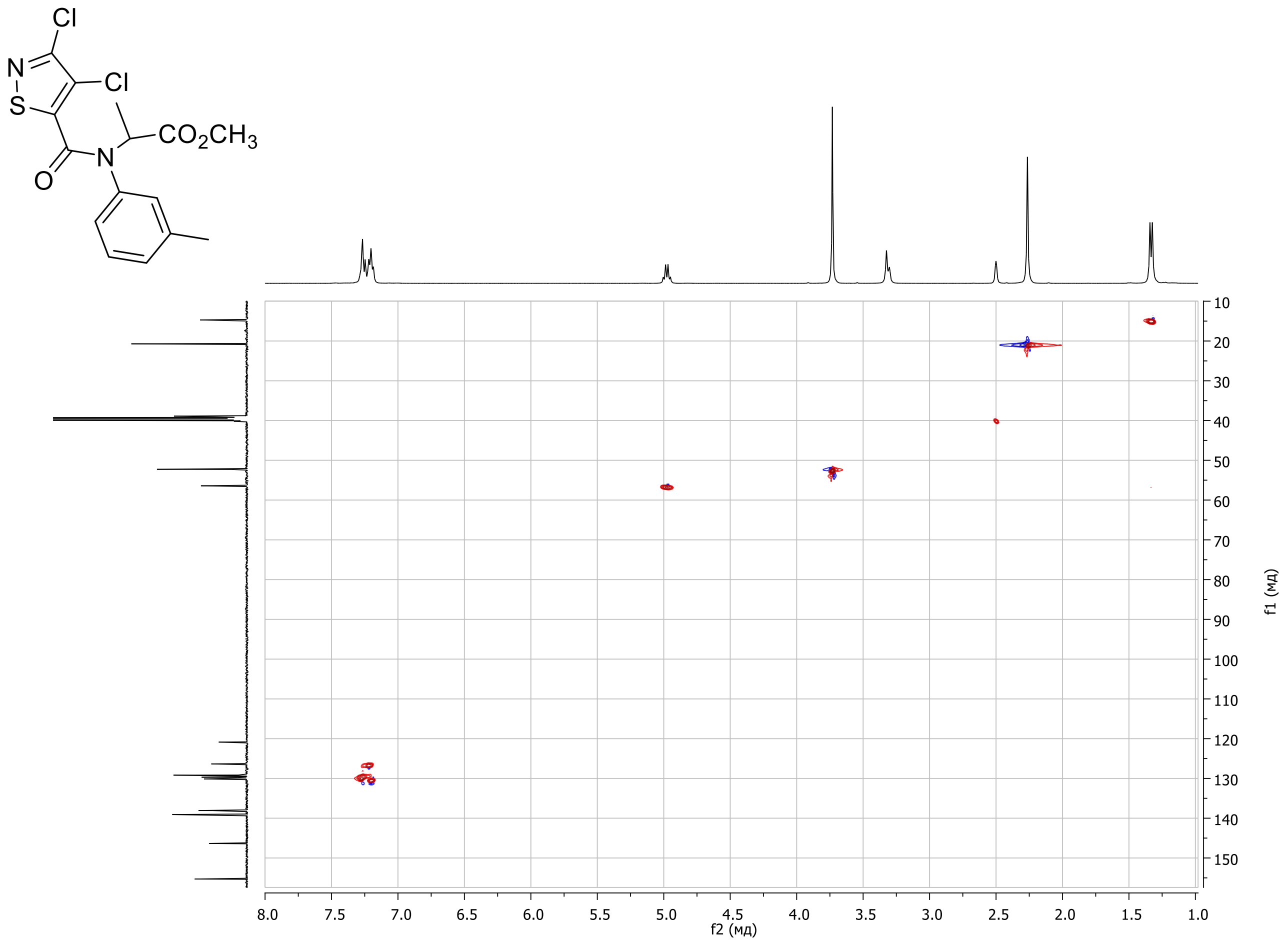

Figure S32. The HSQC ^{1}H–^{13}C NMR spectrum of **2b**

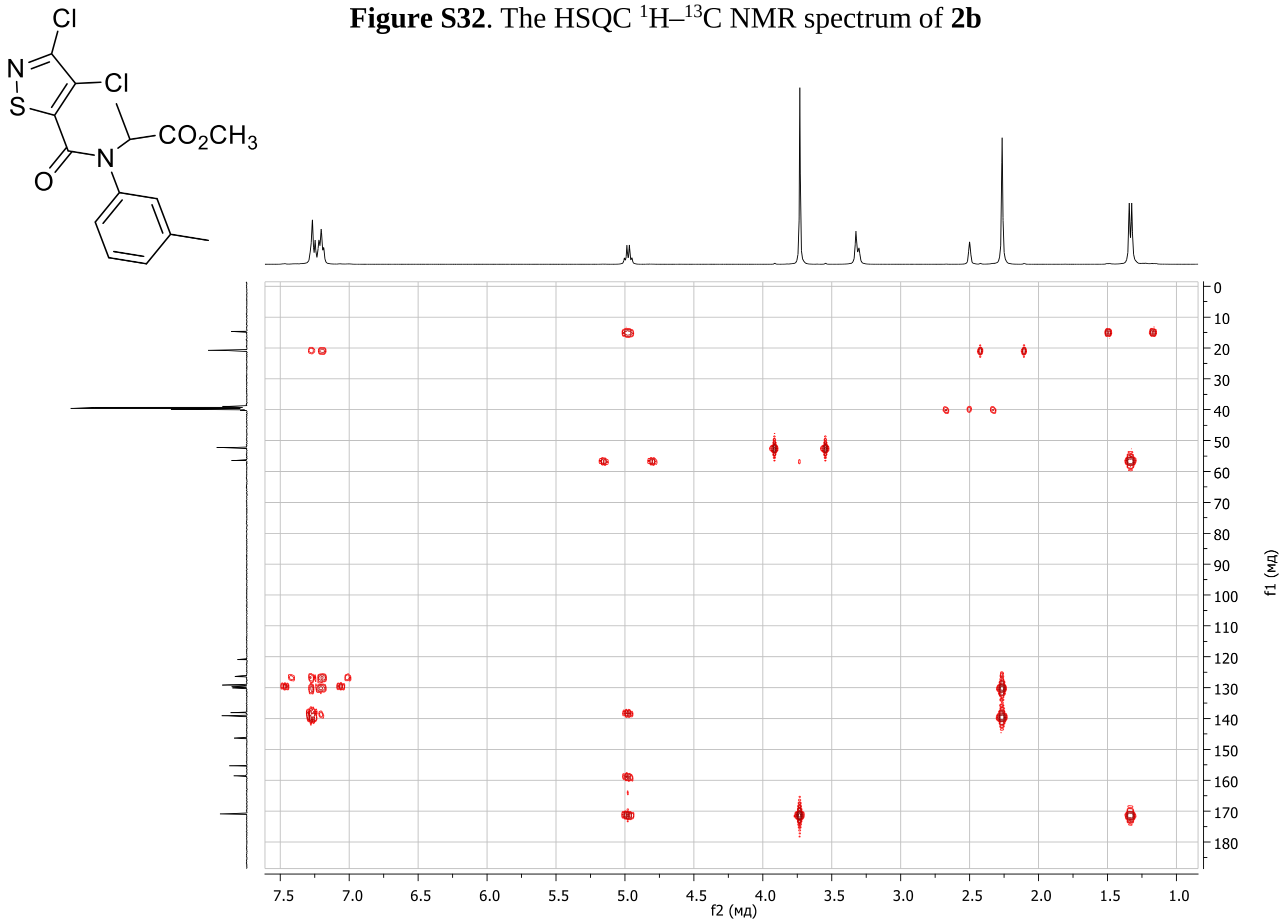

Figure S33. The HMBC ^{1}H–^{13}C NMR spectrum of **2b**

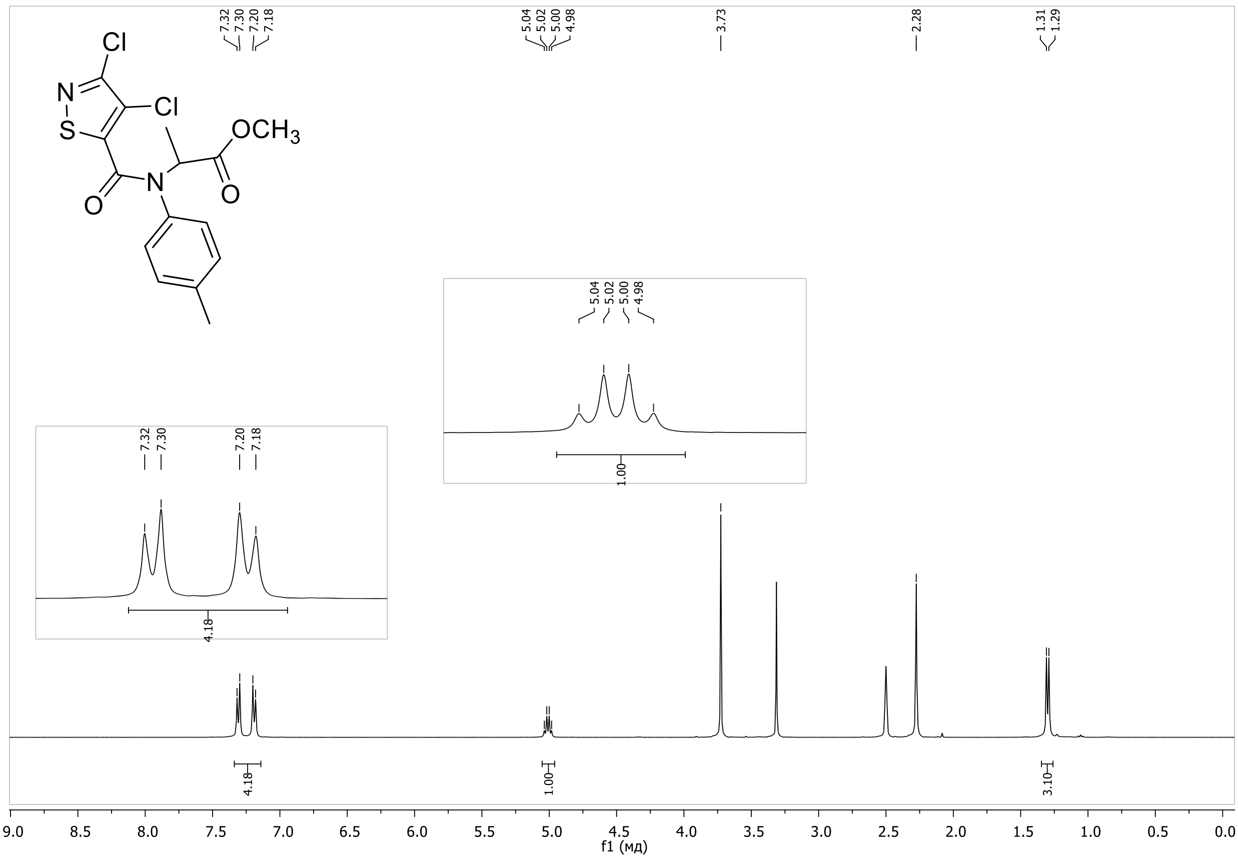

Figure S34. ^{1}H NMR of compound **2c**

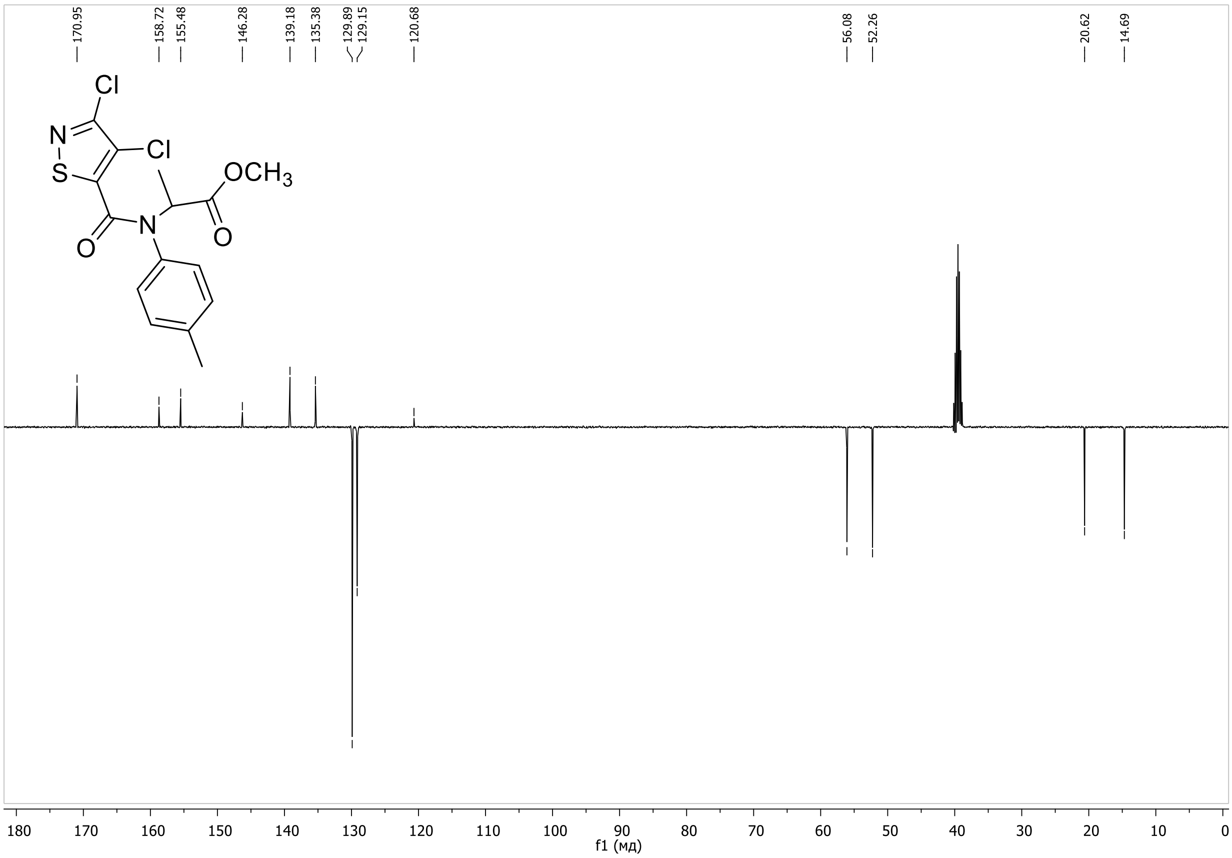

Figure S35. ^{13}C NMR of compound **2c**

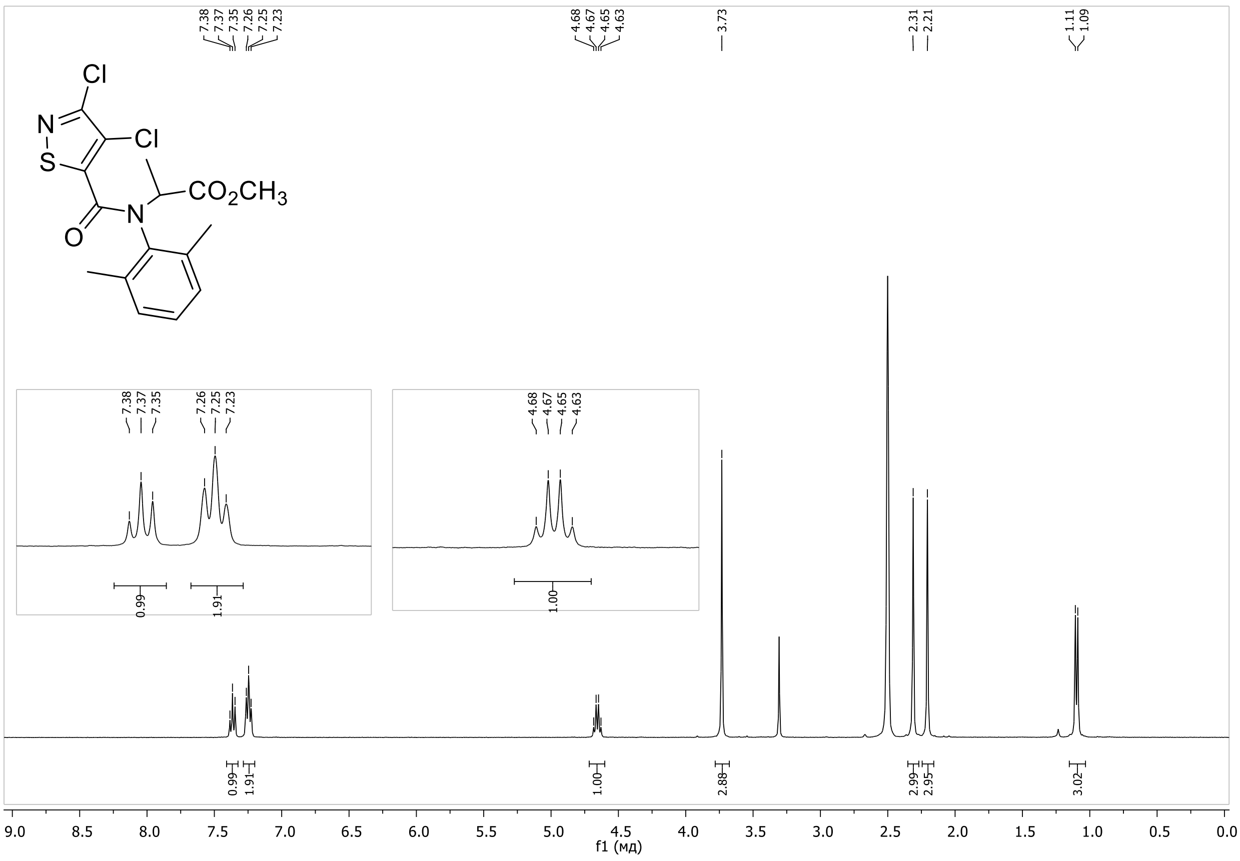

Figure S36. ^{1}H NMR of compound **2d**

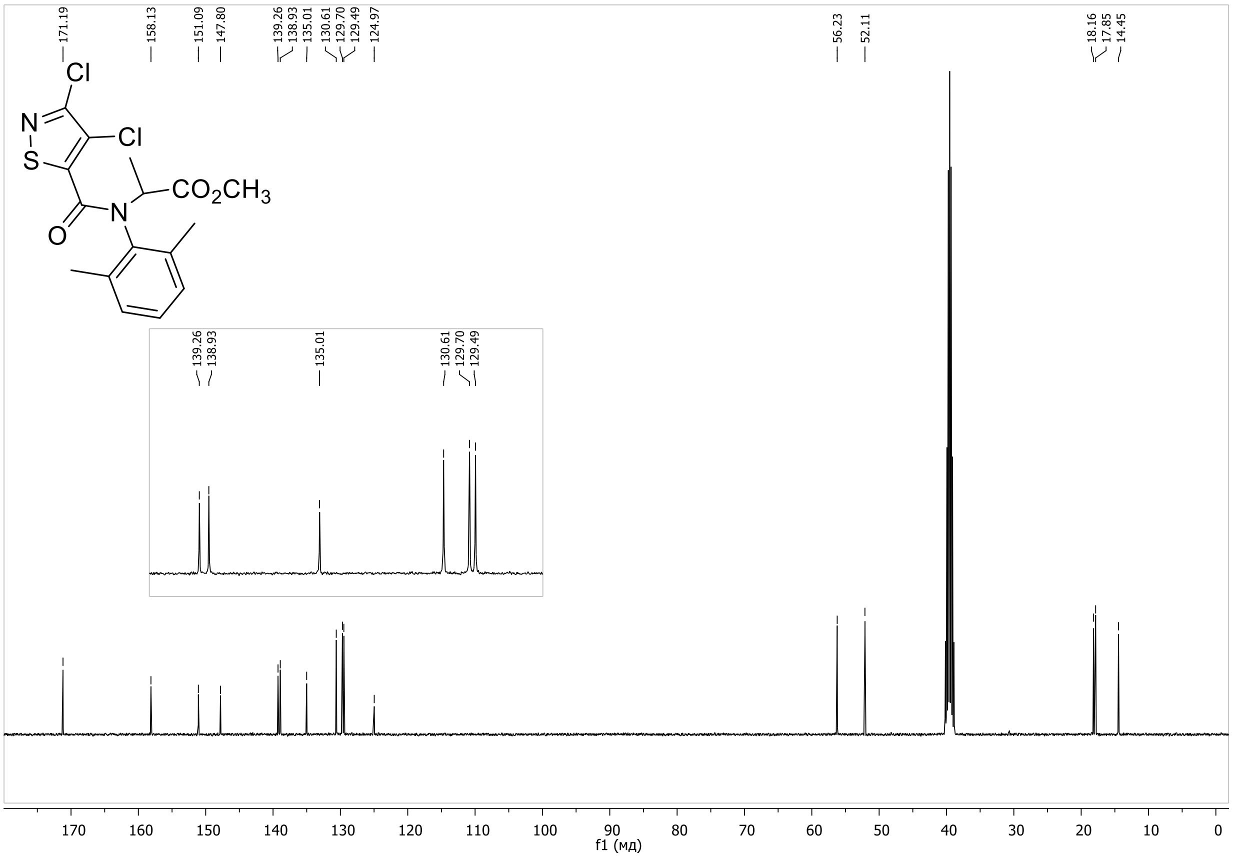

Figure S37. ^{13}C NMR of compound **2d**

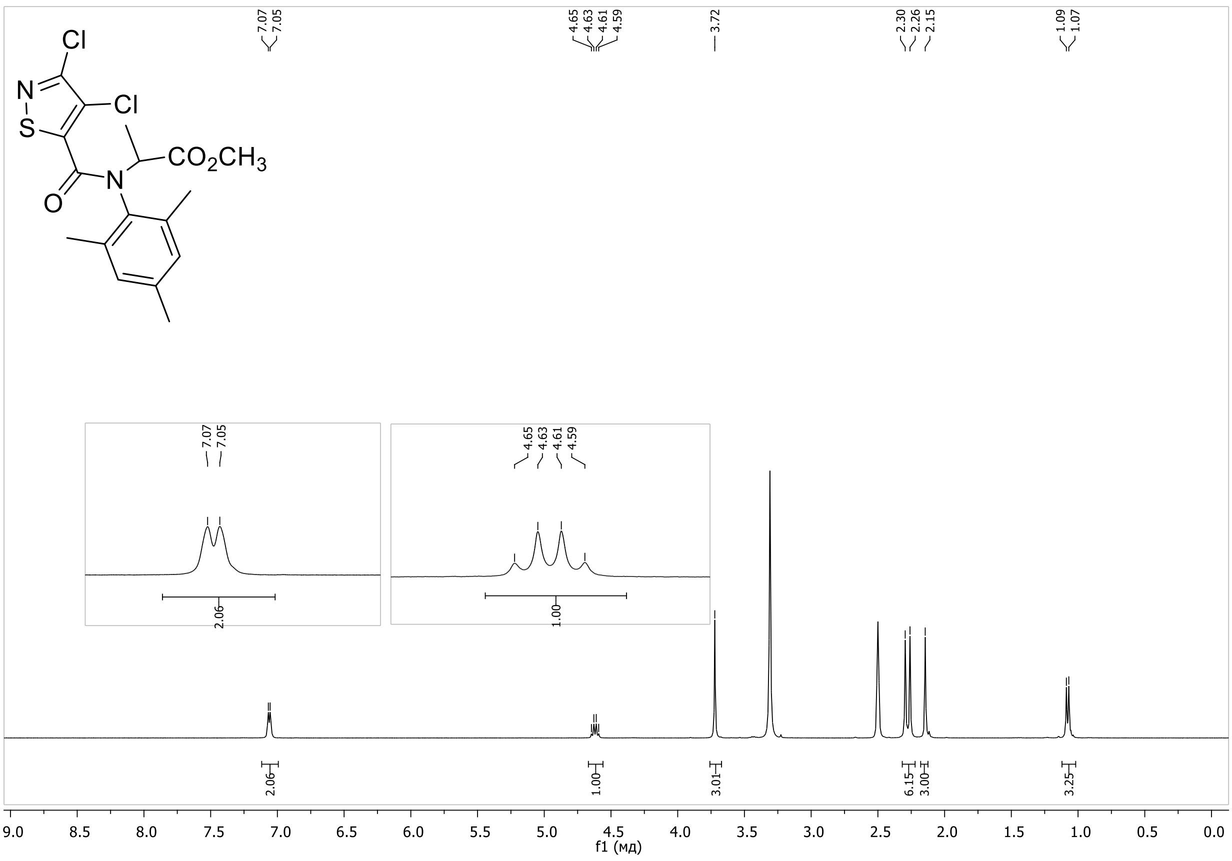

Figure S38. ^{1}H NMR of compound **2e**

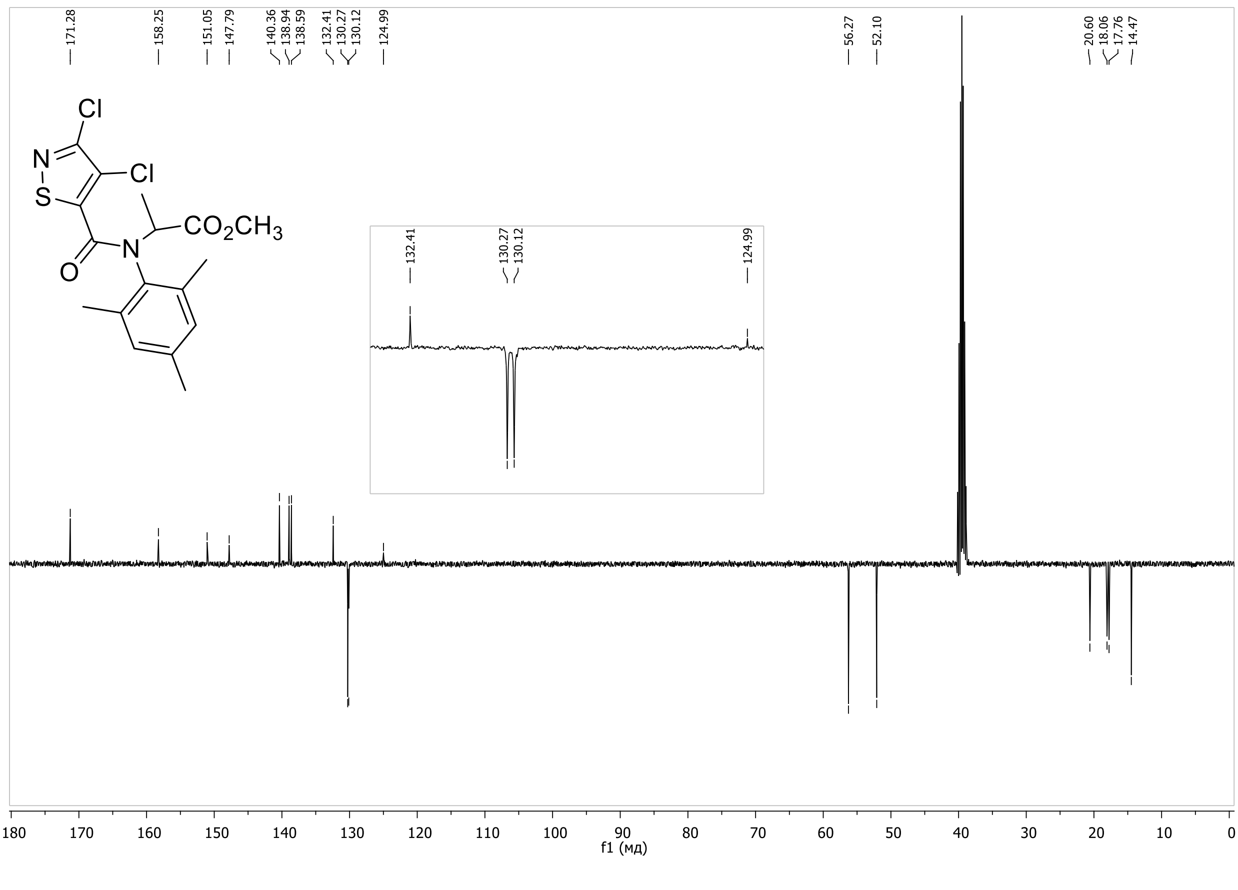

Figure S39. ^{13}C NMR of compound **2e**

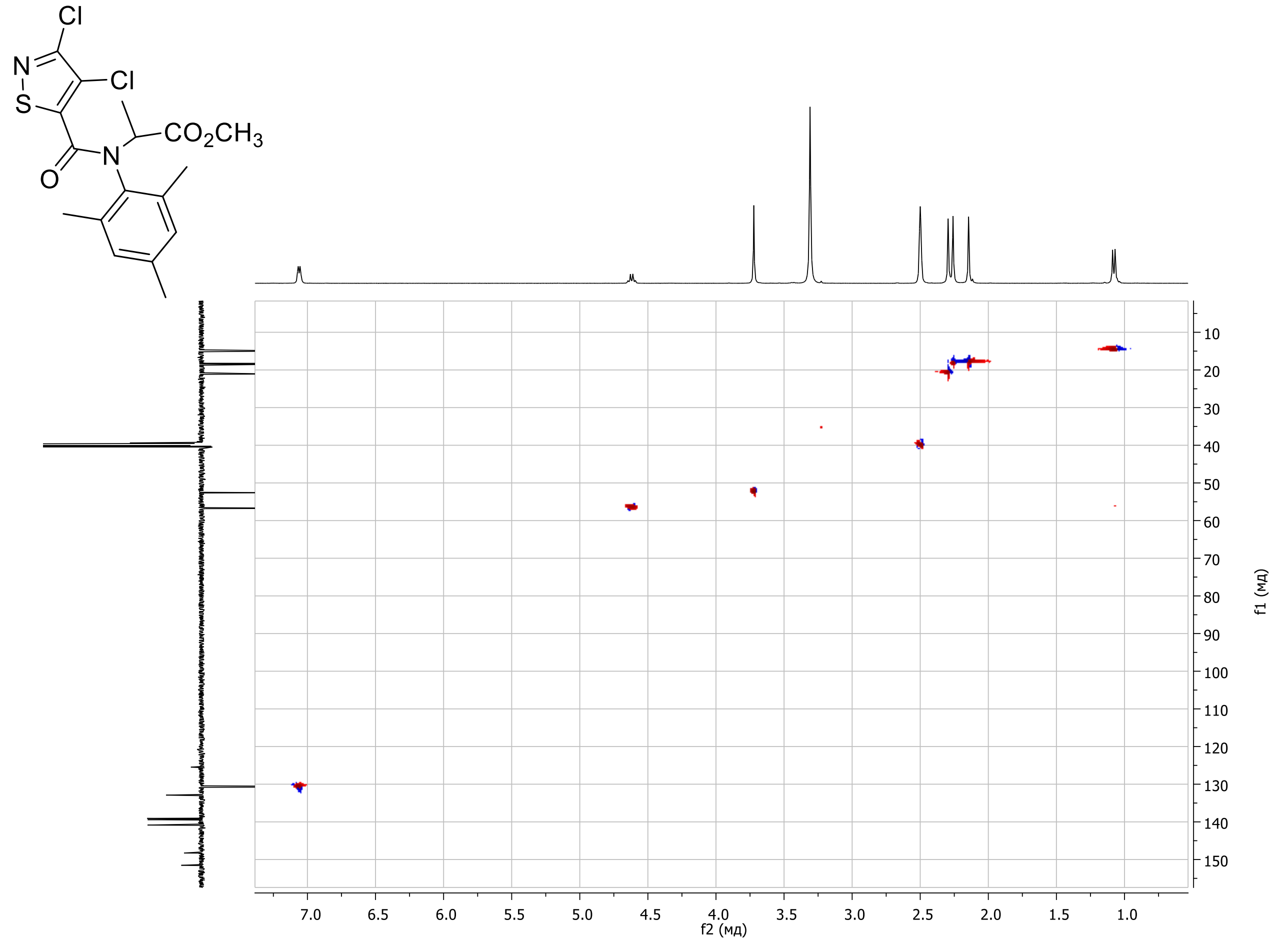

Figure S40. The HSQC ^{1}H–^{13}C NMR spectrum of **2e**

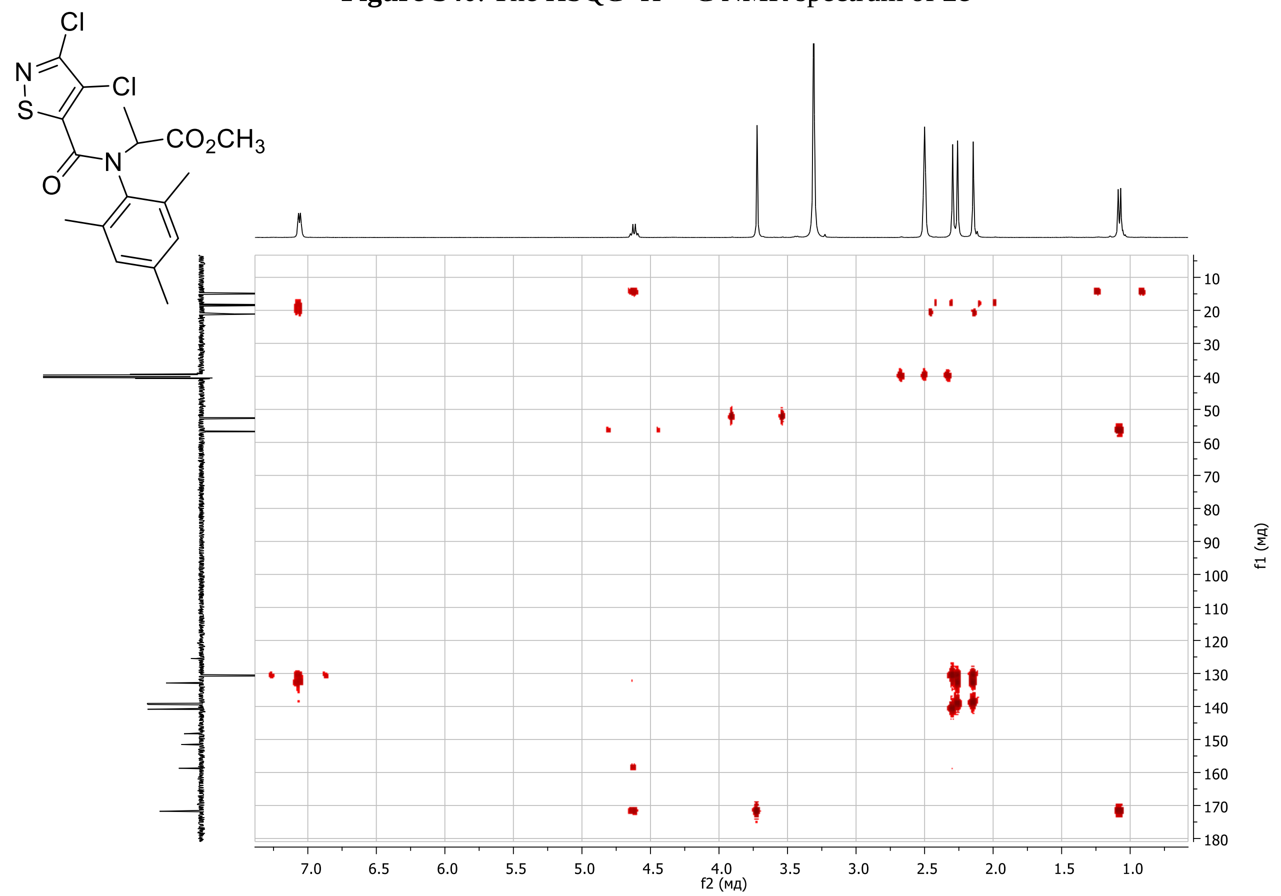

Figure S41. The HMBC ^{1}H–^{13}C NMR spectrum of **2e**

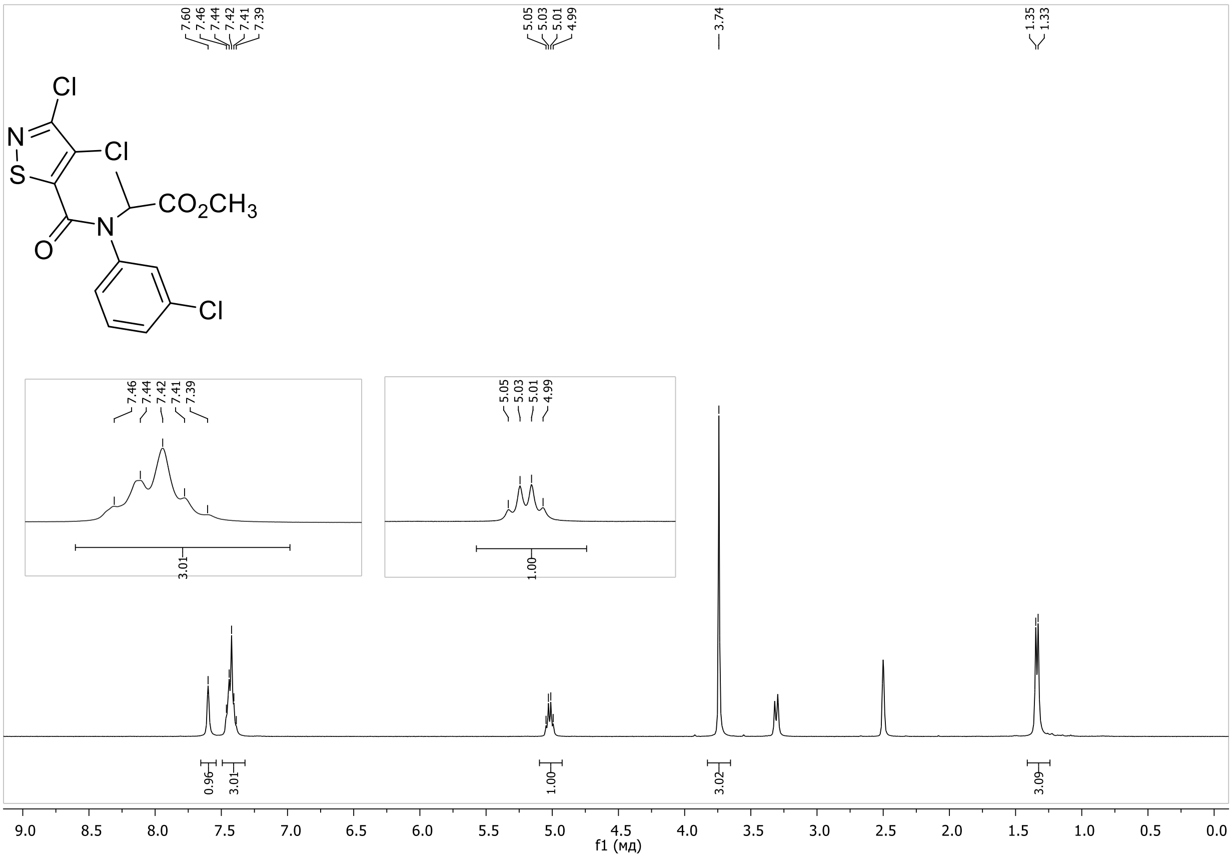

Figure S42. ^{1}H NMR of compound **2f**

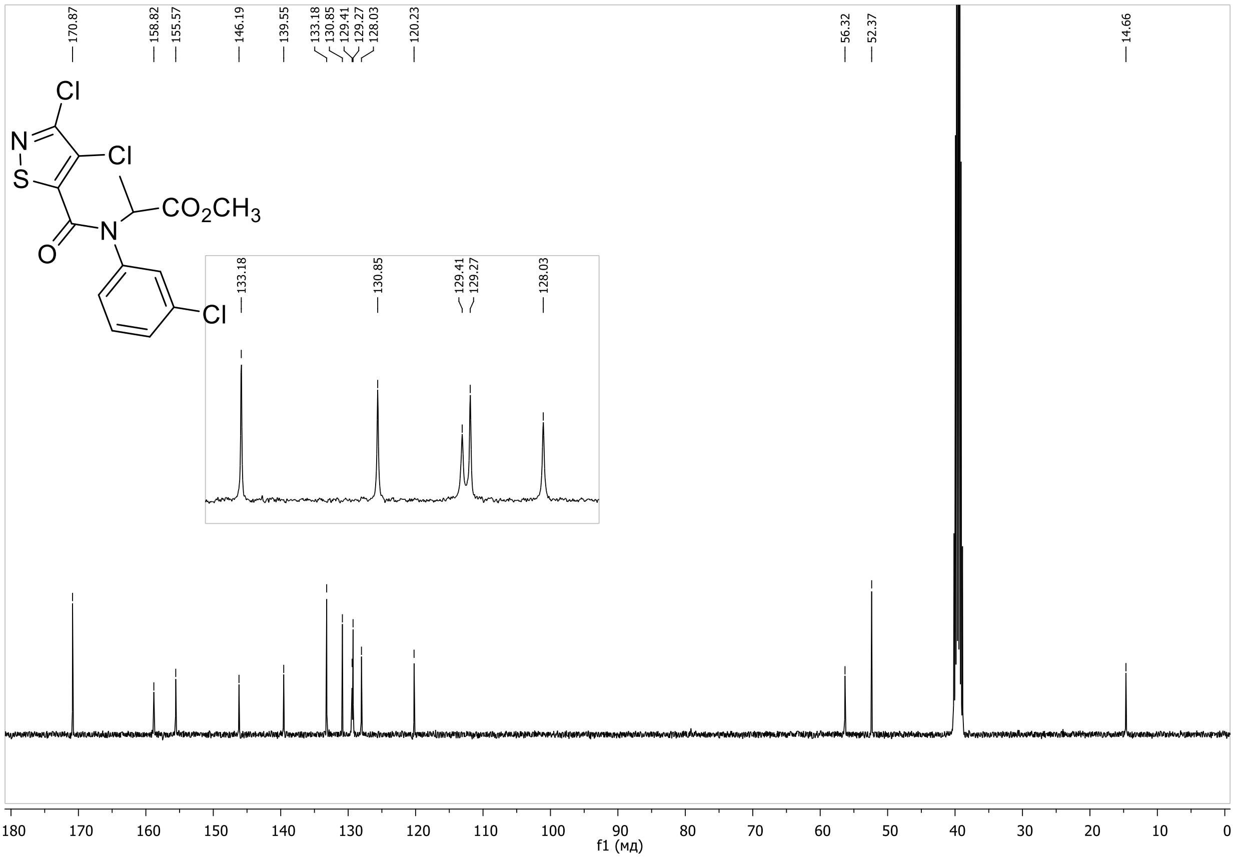

Figure S43. ^{13}C NMR of compound **2f**

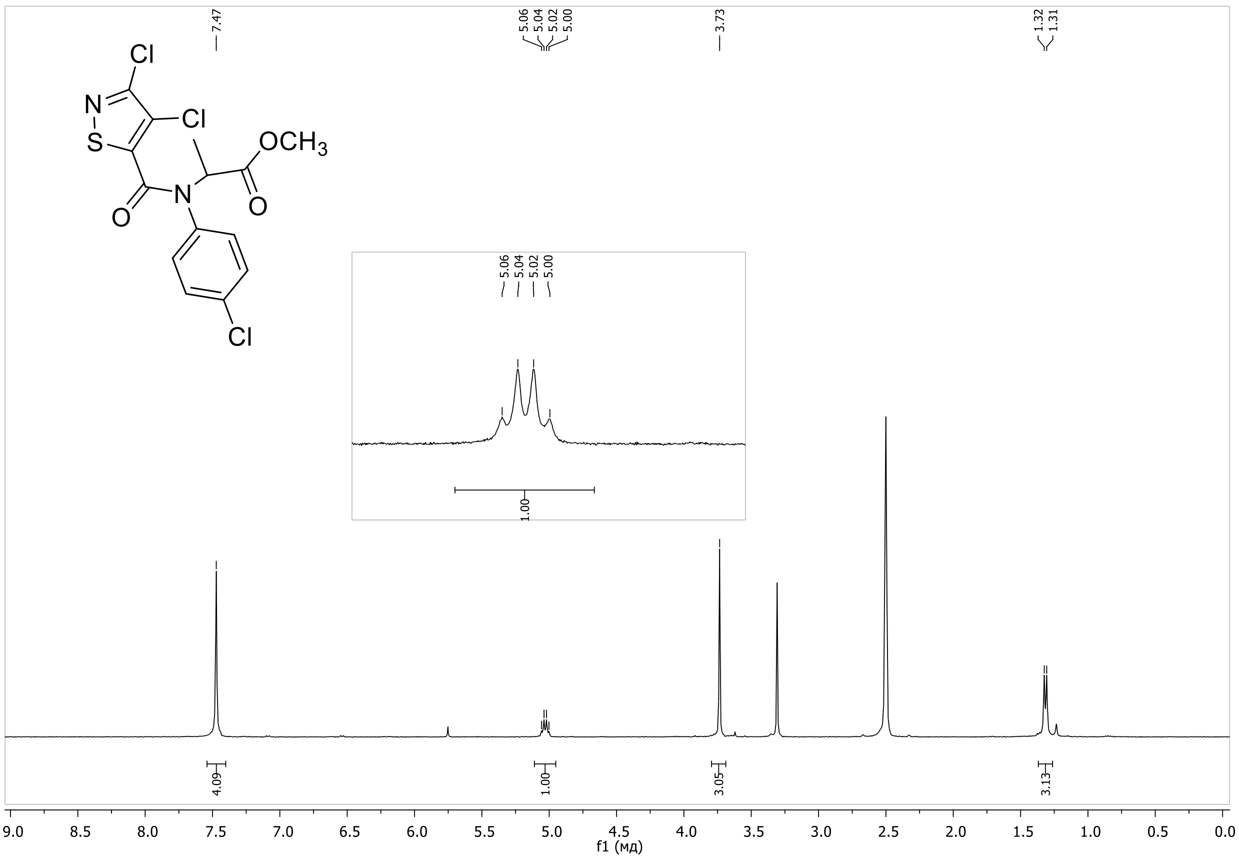

Figure S44. ^{1}H NMR of compound **2g**

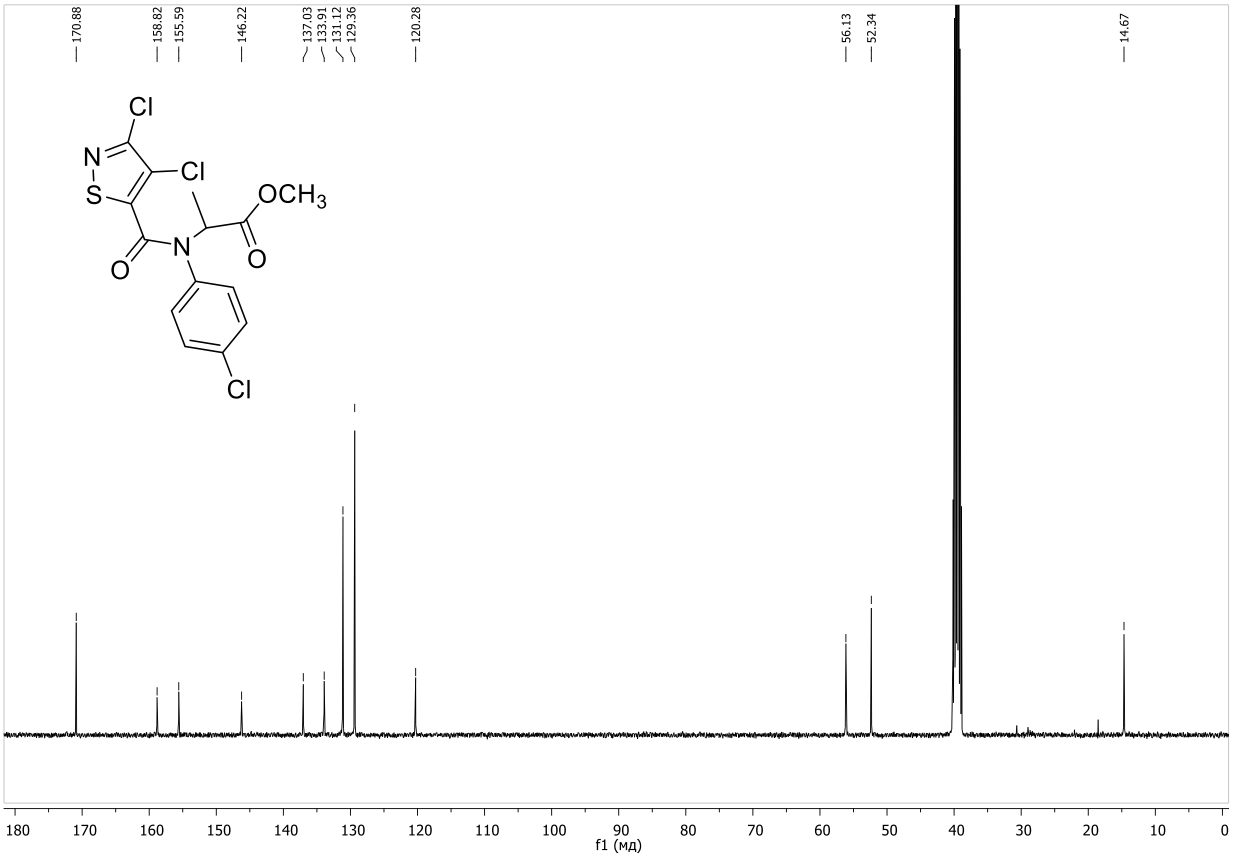

Figure S45. ^{13}C NMR of compound **2g**

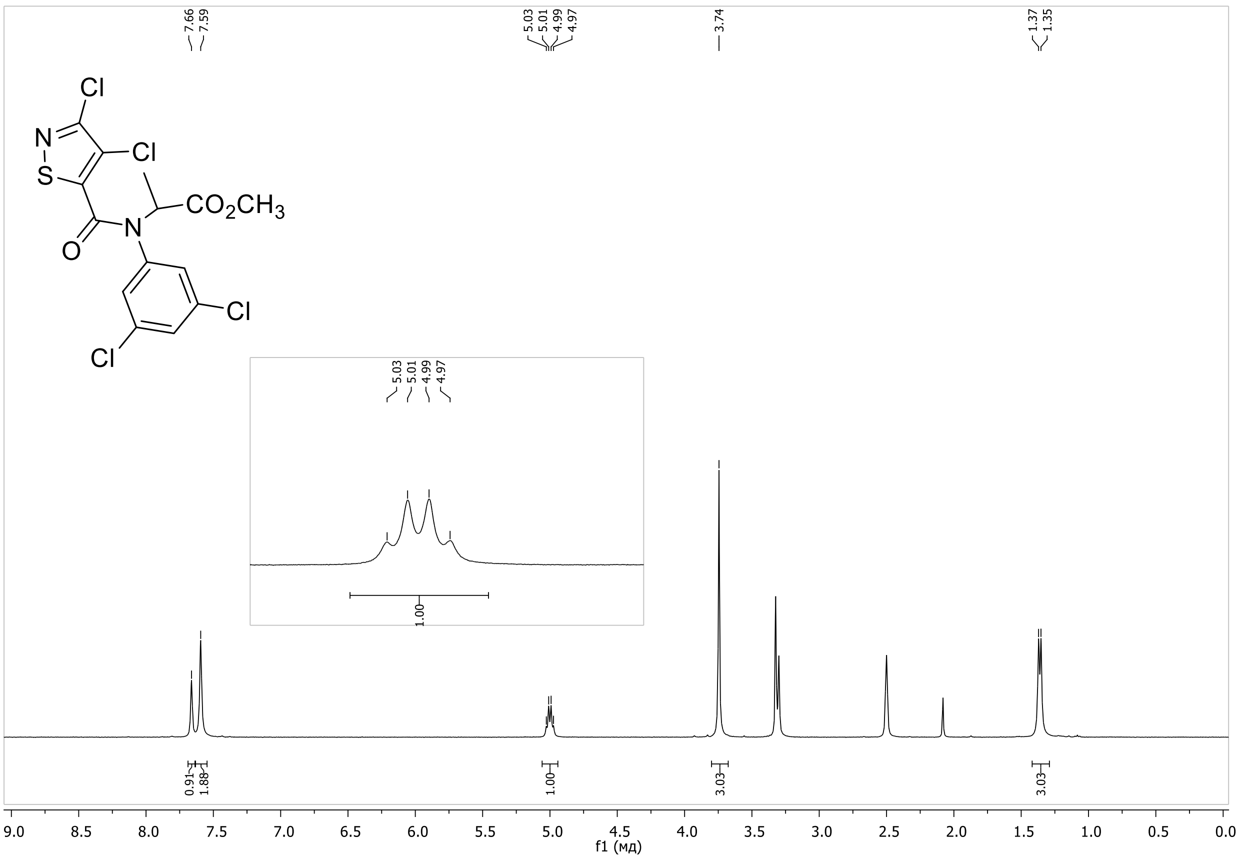

Figure S46. ^{1}H NMR of compound **2h**

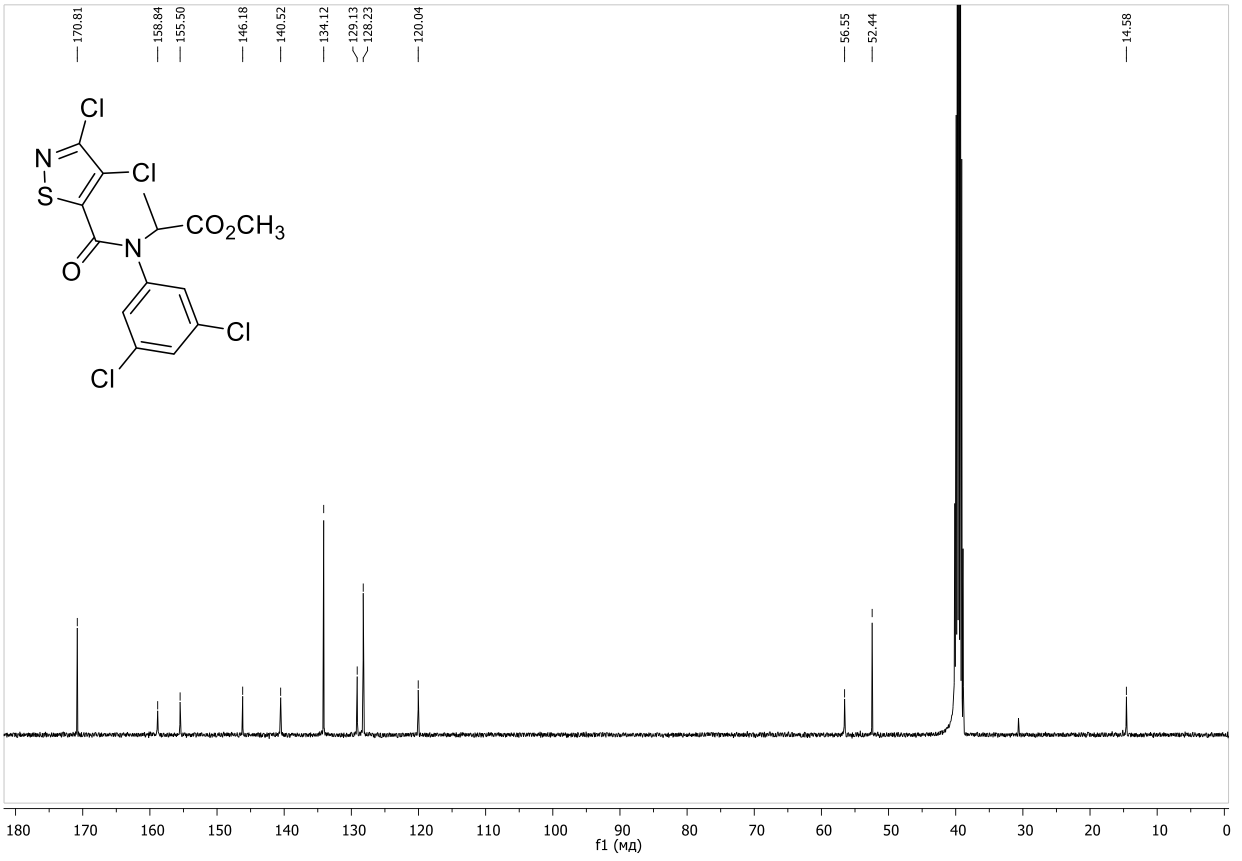

Figure S47. ^{13}C NMR of compound **2h**

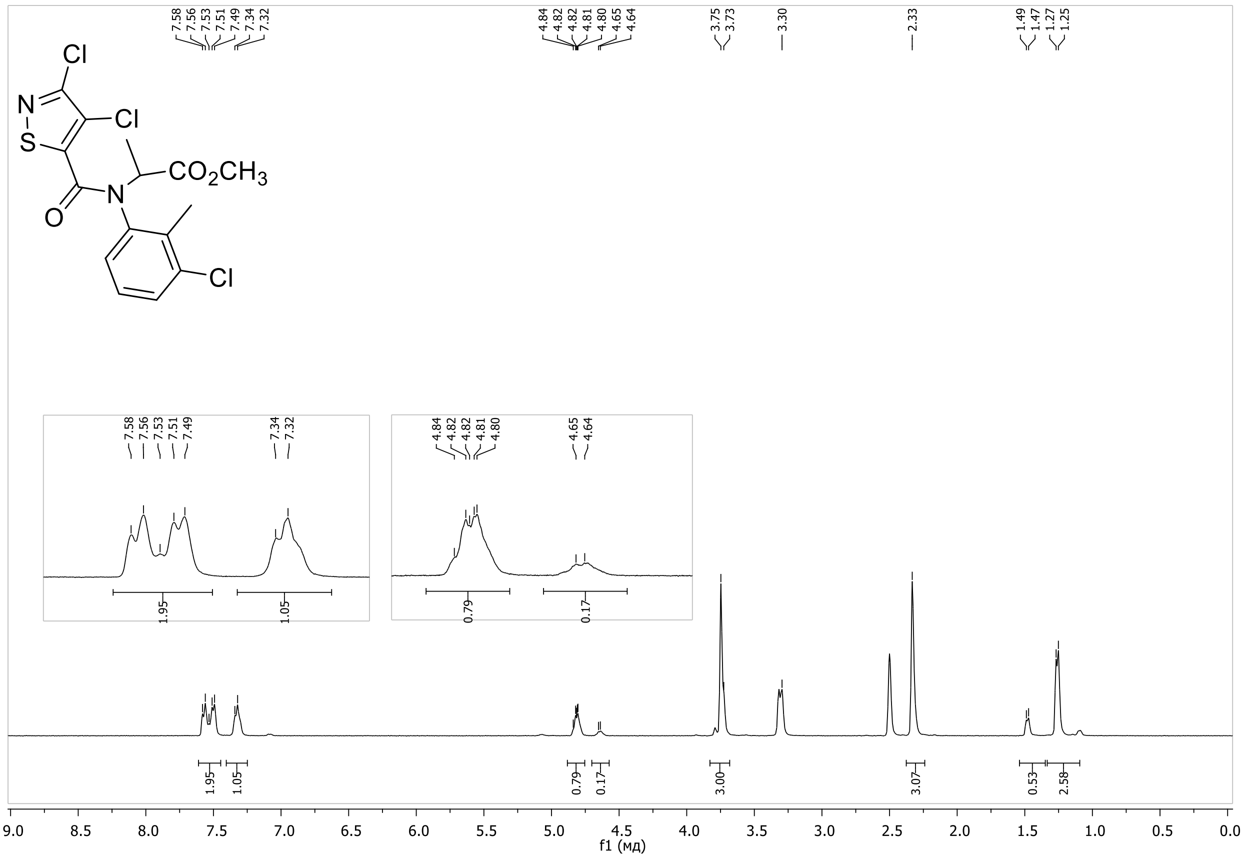

Figure S48. ^{1}H NMR of compound **2i**

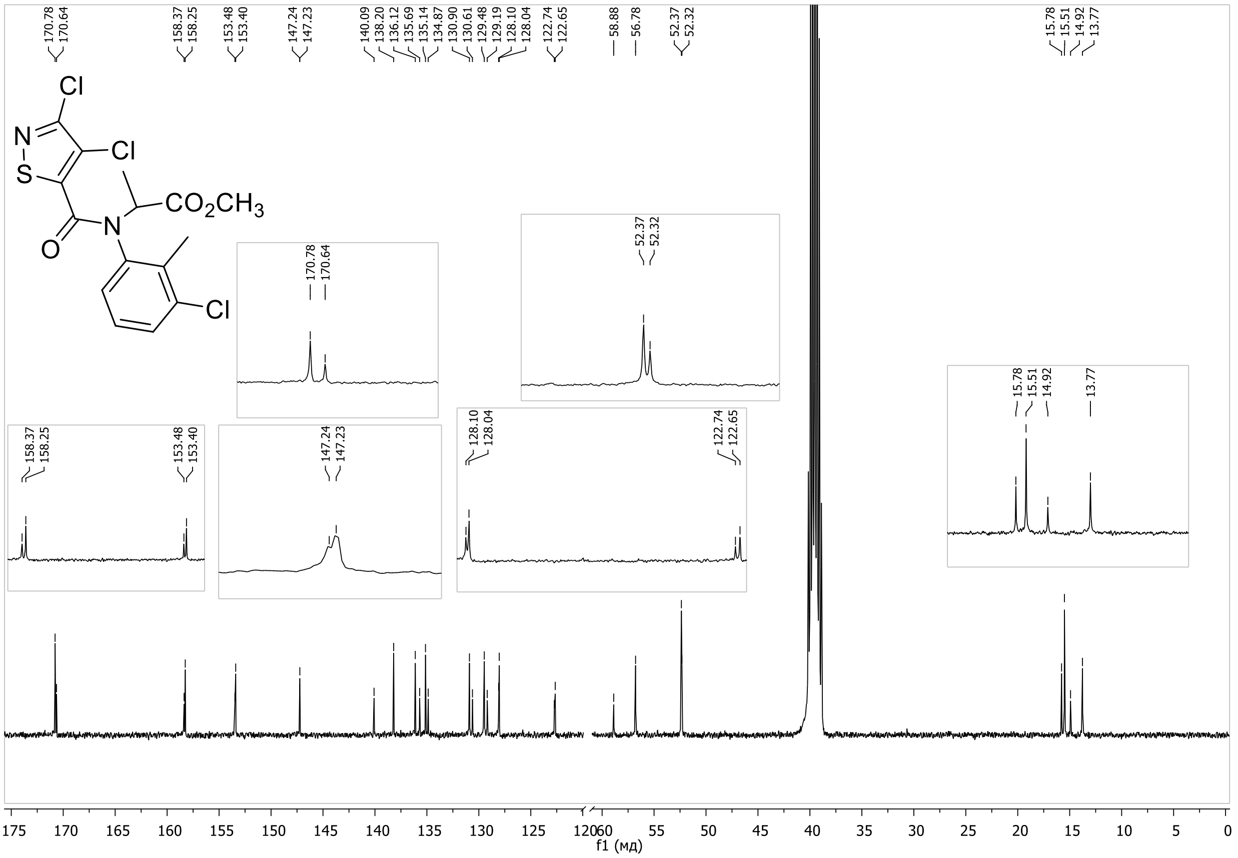

Figure S49. ^{13}C NMR of compound **2i**

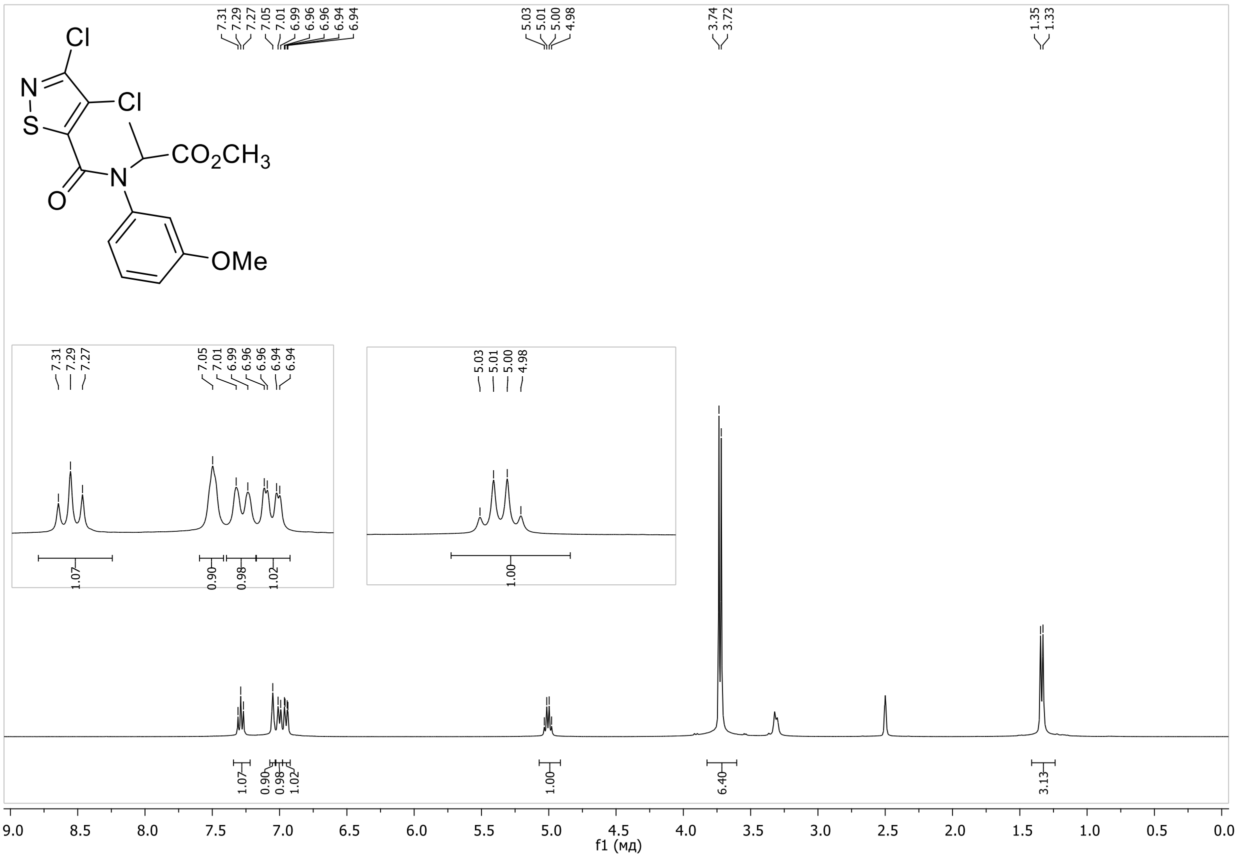

Figure S50. ^{1}H NMR of compound **2j**

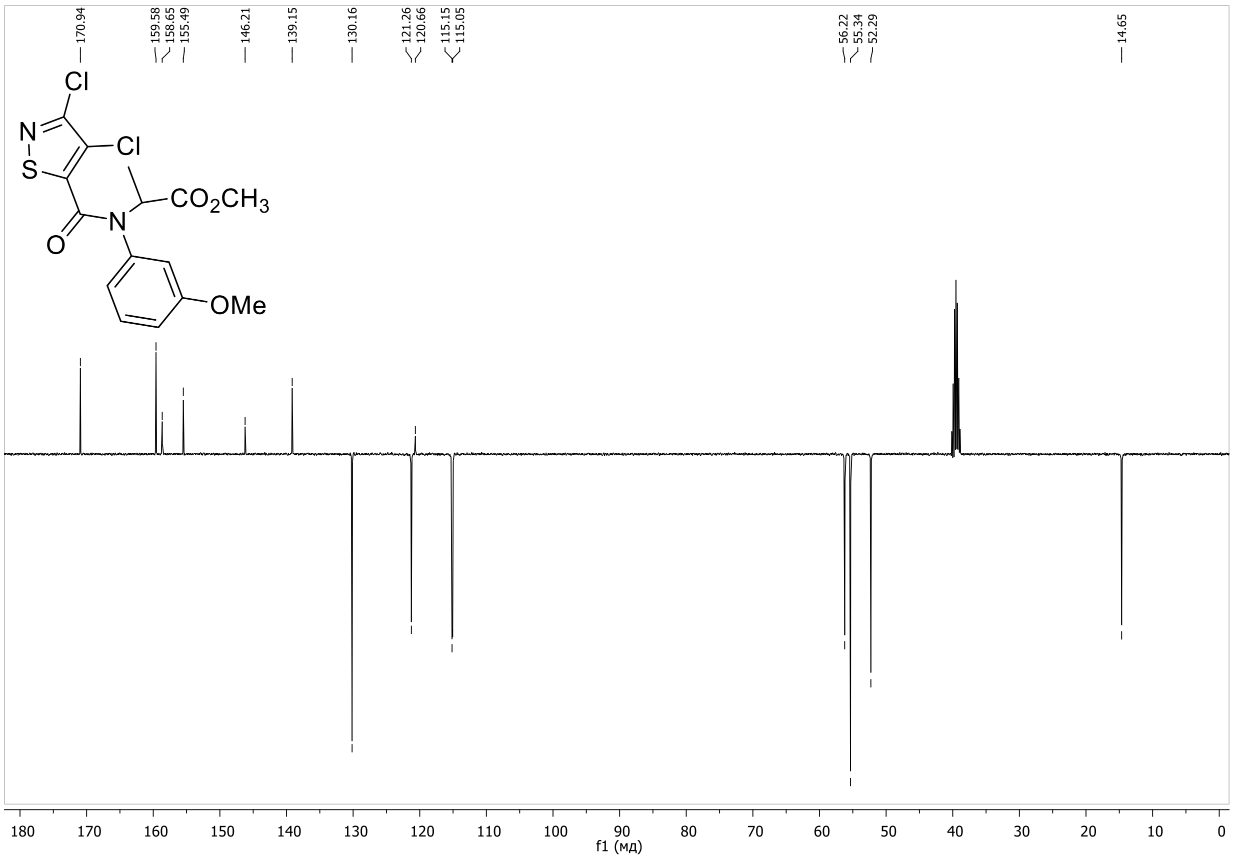

Figure S51. ^{13}C NMR of compound **2j**

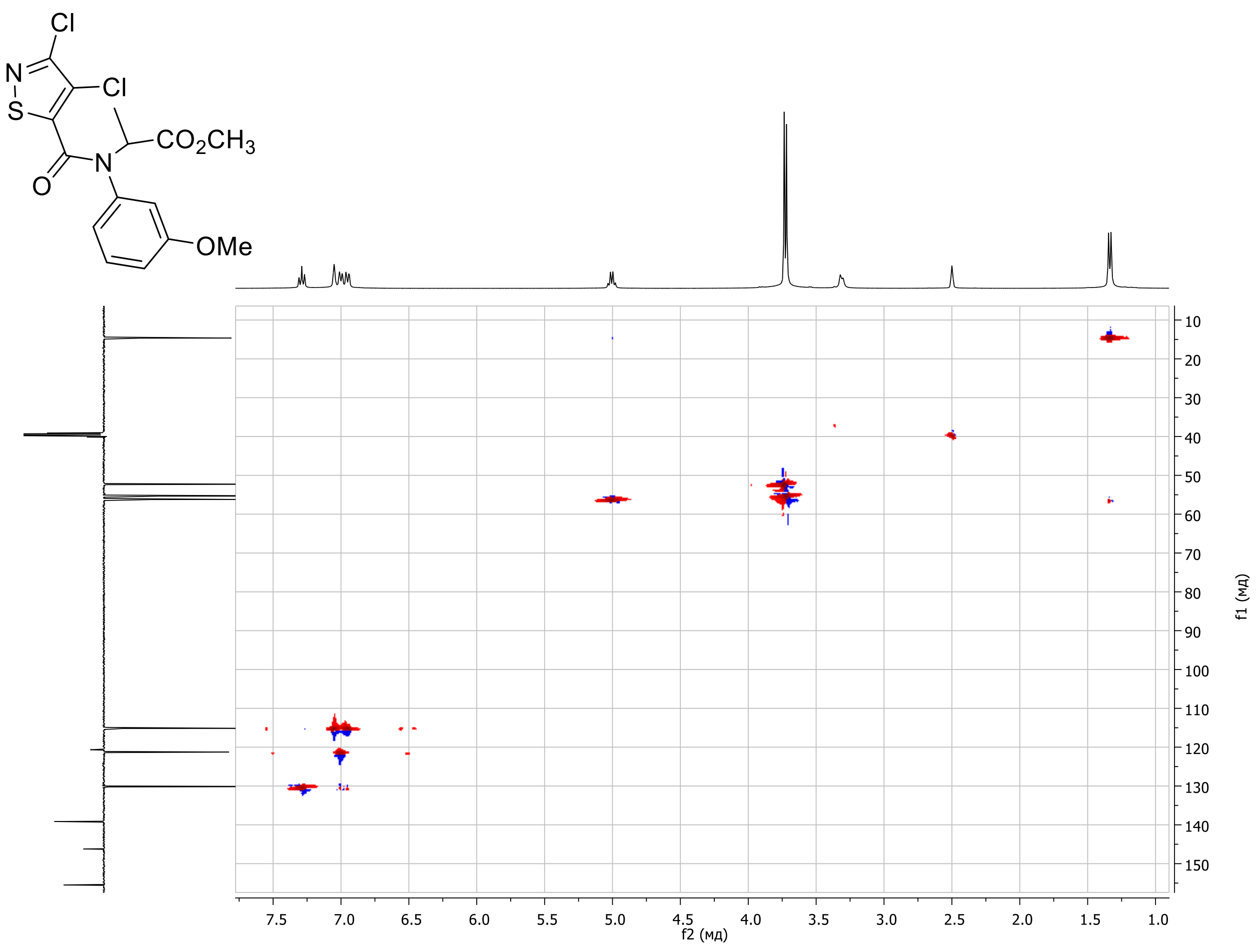

Figure S52. The HSQC ^{1}H–^{13}C NMR spectrum of **2j**

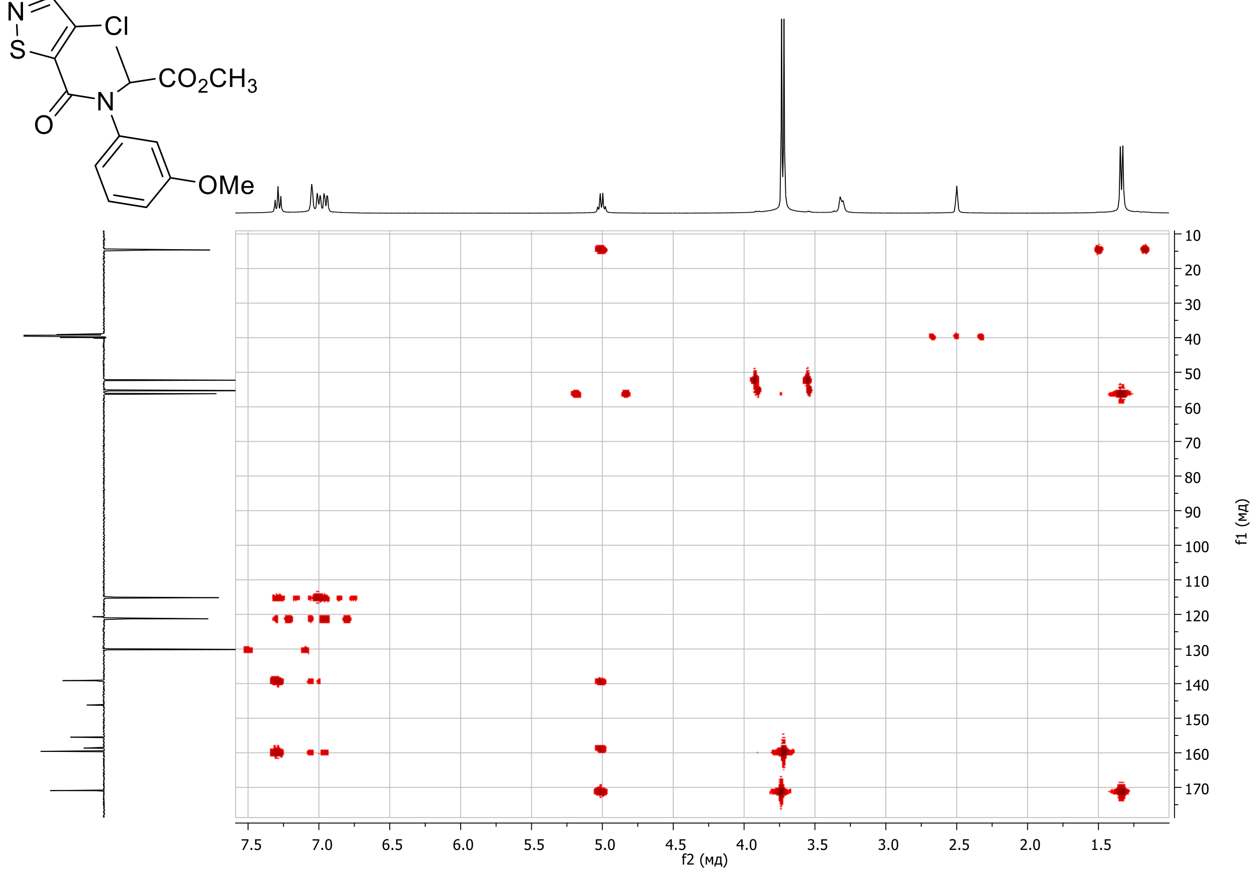

Figure S53. The HMBC ^{1}H–^{13}C NMR spectrum of **2j**

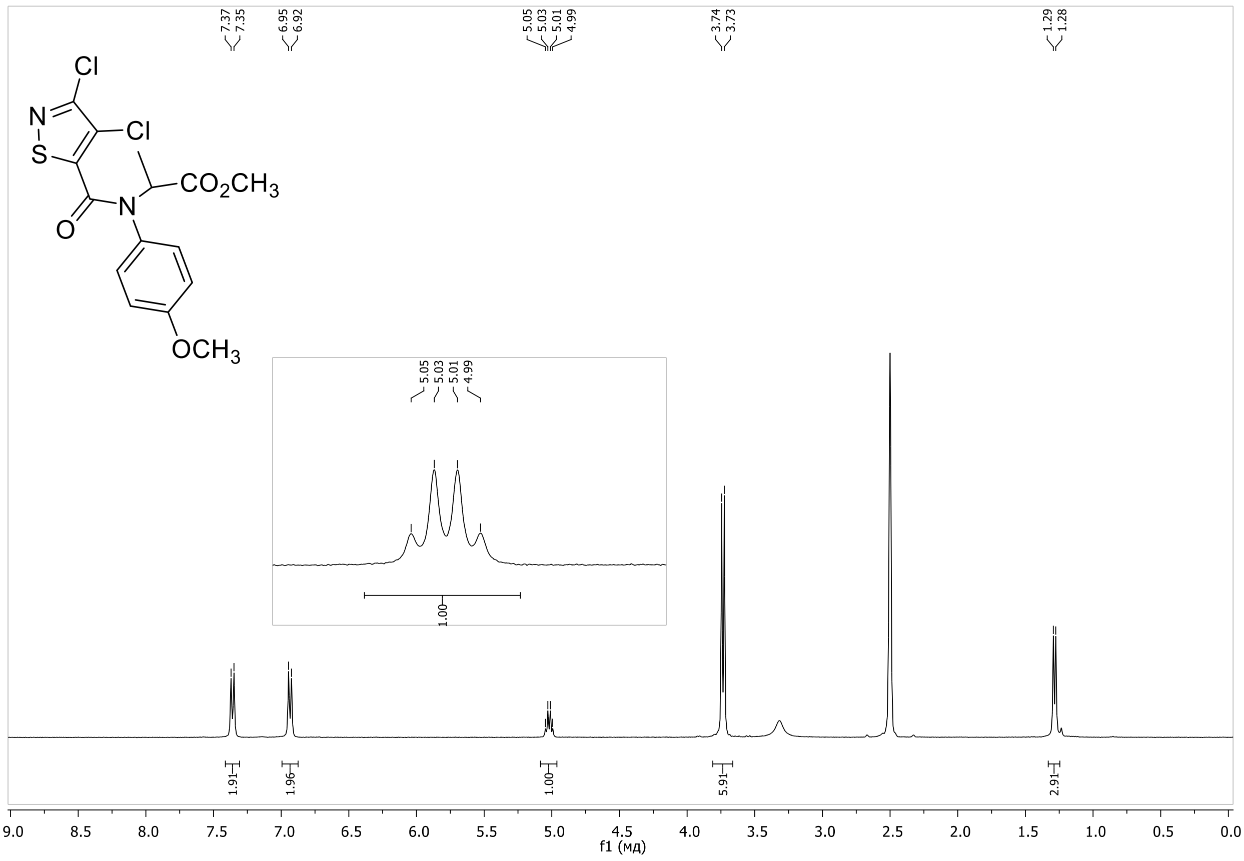

Figure S54. ^{1}H NMR of compound **2k**

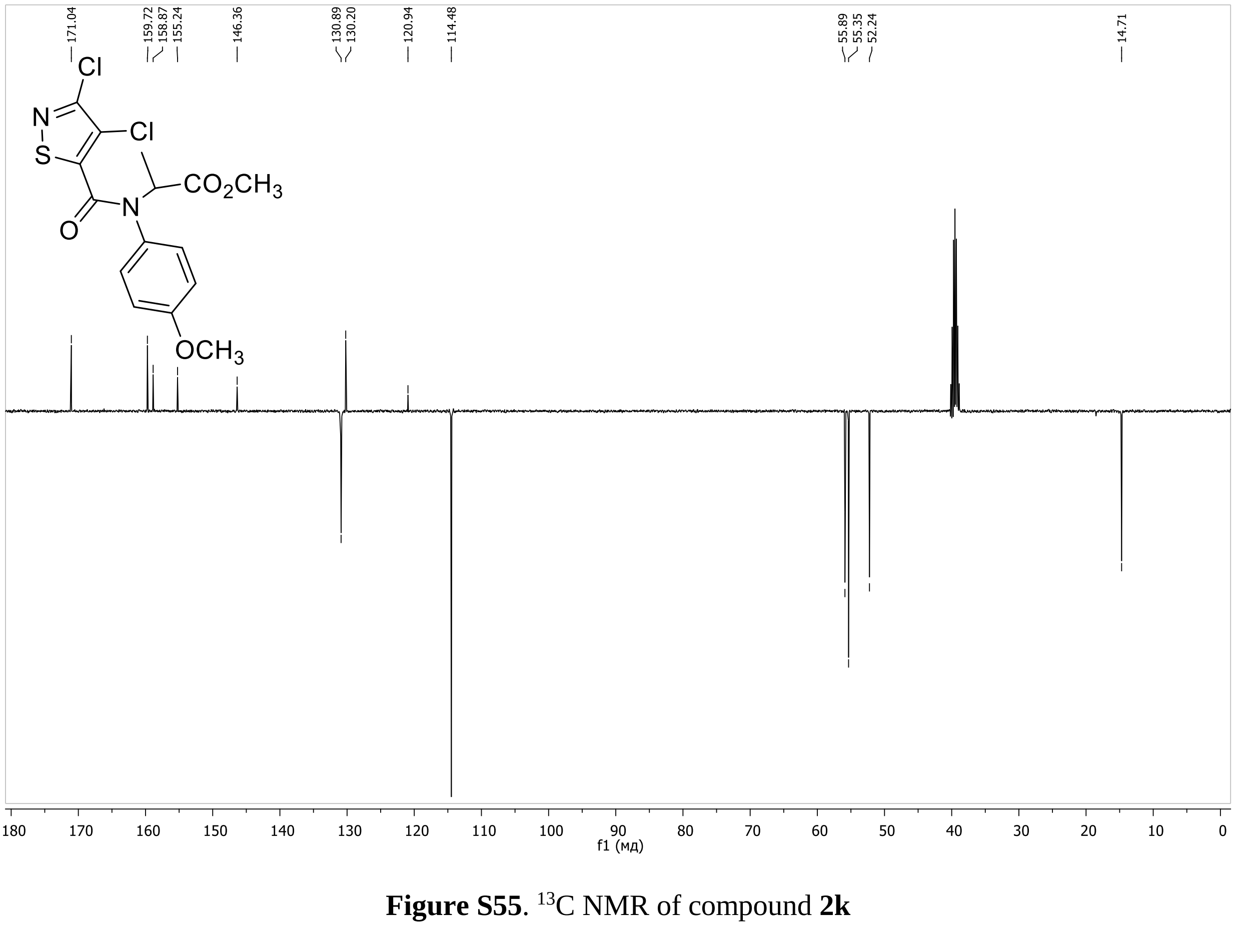

Figure S55. ^{13}C NMR of compound **2k**

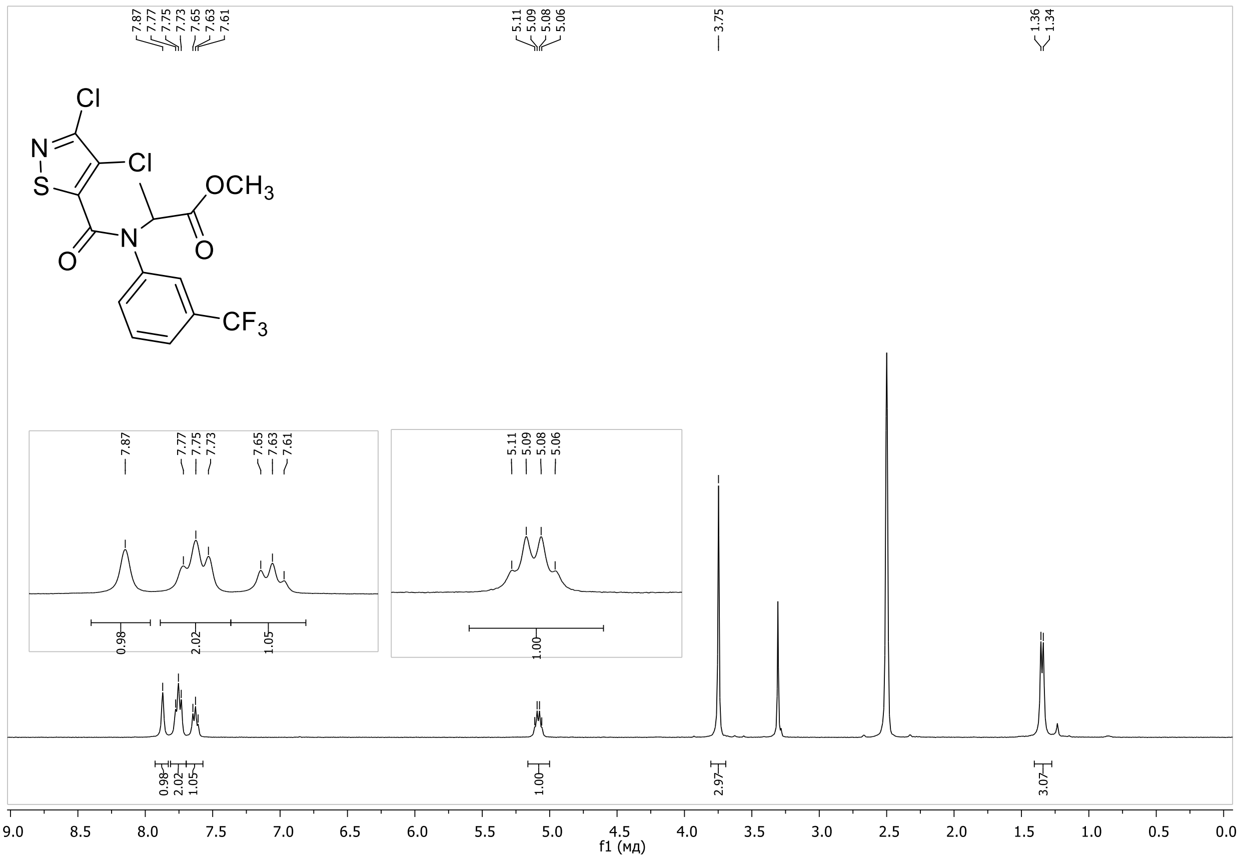

Figure S56. ^{1}H NMR of compound **2l**

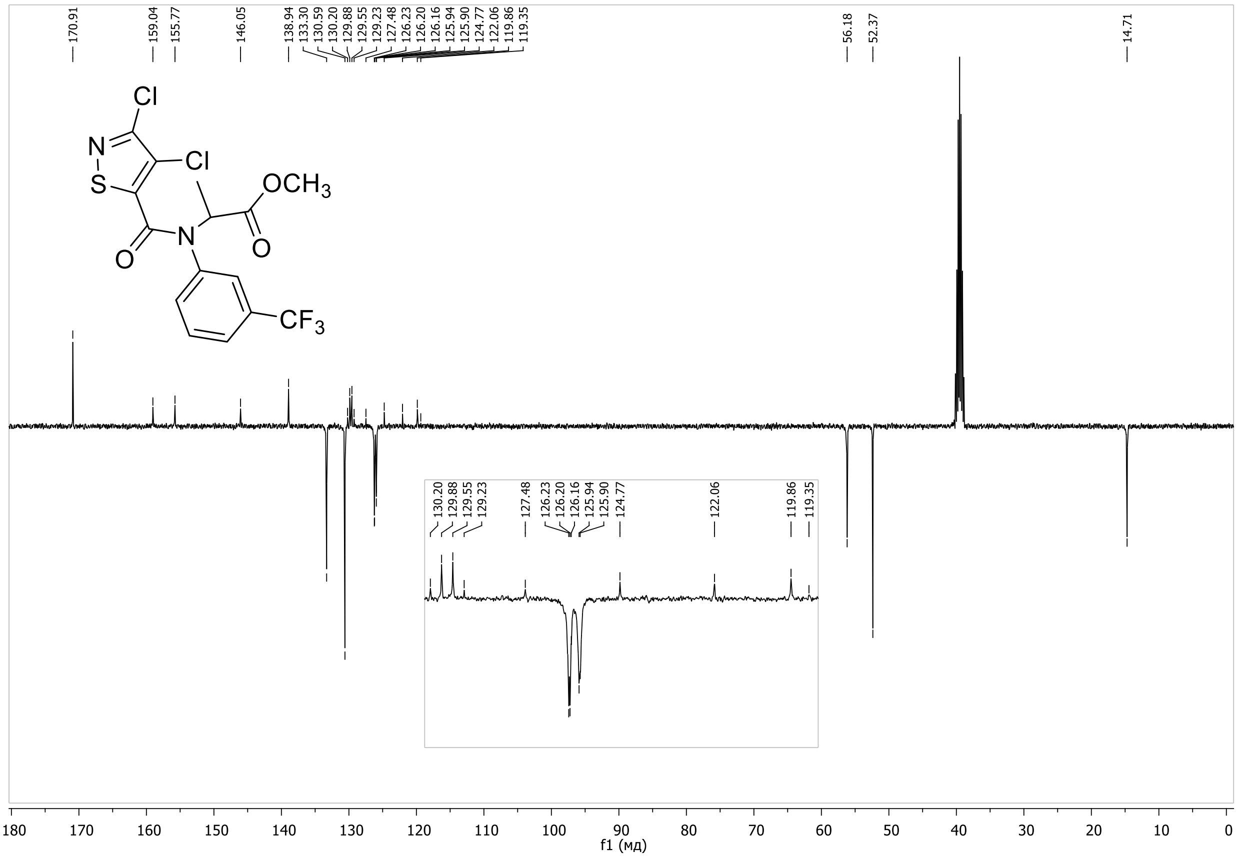

Figure S57. ^{13}C NMR of compound **2l**
